# Abstracts from the 20th European symposium on radiopharmacy and radiopharmaceuticals

**DOI:** 10.1186/s41181-023-00193-4

**Published:** 2023-06-15

## OP02

### Combined radionuclide and hyperthermia cancer therapy with superparamagnetic iron oxide nanoparticles coupled to ^131^I-labeled antibodies

#### Aljoša Stanković^1^, Marija Mirković^2^, Magdalena Radović^2^, Zorana Milanović^2^, Aleksandar Vukadinović^2^, Drina Janković^2^, Sanja Vranješ-Đurić^2^

##### ^1^University Clinical Centre of the Republic of Srpska, Clinical Department for Nuclear Medicine and Thyroid Gland Disease, Bosnia and Herzegovina. ^2^Vinča Institute of Nuclear Sciences, University of Belgrade, Belgrade, Serbia

*EJNMMI Radiopharmacy and Chemistry* 2023; 8(Suppl1): OP02

**Aim:** Nanotechnology showed great potential in selective delivery of therapeutic agents and reducing their side effects. The incorporation of radionuclide in superparamagnetic iron oxide nanoparticles (SPIONs) could lead to the development of multifunctional agents with greater potential in cancer therapy due to the combined effect of therapeutic radiation and hyperthermia. This study aimed to investigate the possibility of ^131^I-radiolabeled SPIONs with excellent heating efficiency for nano-brachytherapy of tumors.

**Materials and methods:** SPIONs were synthesized using the polyol method. The biocompatibility of SPIONs was improved by coating with 3-aminopropyltriethoxysilane (APTES). ^131^I radionuclide was linked to CC49 antibody specific for tumor-associated glycoprotein (TAG-72) before being covalently attached to coated SPIONs. Following intravenous and intratumor injection of the SPIONs complex, biodistribution, retention, and therapeutic effect were investigated on LS174T human colon adenocarcinoma xenografts on NOD-SCID mice.

**Results:** SPIONs were successfully coated with APTES and characterized, by strong heating ability under an oscillating magnetic field. A significant level of radiation and hyperthermia over 45 °C was achieved locally for up to 14 days, demonstrating the stability of the radiolabeling and efficient nanoparticles retention only after intratumor application. Combined radionuclide-hyperthermia therapy showed significant (*P* < 0.01) suppression of the tumor growth when compared to the control groups and was better than radionuclide or hyperthermia treatments alone. Histopathology analysis proved the necrosis and apoptosis present in treated tumors but neither in non-treated ones nor normal organs. By judging the animals’ body mass no general toxicity was observed.

**Conclusion:** Combined therapy by applying coated SPIONs conjugated with ^131^I-radiolabeled antibody was shown as a promising modality for tumor therapy. Obtained results justify further investigation in this encouraging nano-brachytherapy approach.

**References**

1. Żuk M, Podgórski R, Ruszczyńska A, Ciach T, Majkowska-Pilip A, Bilewicz A, et al. Multifunctional nanoparticles based on iron oxide and gold-198 designed for magnetic hyperthermia and radionuclide therapy as a potential tool for combined HER2-positive cancer treatment. Pharmaceutics. 2022;14(8):1680.
2. Zhang Y, Sheng J, Zhai F, Wang X, Chen L, Shi C, et al. Pioneering iodine-125-labeled nanoscale covalent organic frameworks for brachytherapy. Bioconjug Chem. 2021;32(4):755–62.
3. Kadkhoda J, Akrami-Hasan-Kohal M, Tohidkia MR, Khaledi S, Davaran S, Aghanejad A. Advances in antibody nanoconjugates for diagnosis and therapy: A review of recent studies and trends. Int J Biol Macromol. 2021;185(June):664–78.

## OP03

### Conjugates of cerium dioxide nanoparticles with macrocyclic ligands for radiopharmaceuticals: in vitro and in vivo stability

#### G.Yu. Aleshin^1^, S.Yu. Khabirova^1^, M.A. Menshikov^1^, A.A. Shchukina^2^, A.D. Zubenko^2^, O.A. Fedorova^2^, A.A. Averin^3^, S.N. Kalmykov^1^

##### ^1^Lomonosov Moscow State University, Moscow, Russia. ^2^A. N. Nesmeyanov Institute of Organoelement Compounds of Russian Academy of Sciences, Moscow, Russia. ^3^Frumkin Institute of Physical chemistry and Electrochemistry Russian Academy of Sciences, Leninskiy ave. 31b4, Moscow, Russia

*EJNMMI Radiopharmacy and Chemistry* 2023; 8(Suppl1): OP03

**Aim:** A relatively new and promising area of research in nuclear medicine is the development of biocompatible nanomaterials labelled with radionuclides for the early diagnosis and effective treatment of oncological diseases. Nanoparticles are a convenient platform for creating combined radiopharmaceuticals. At the same time, nanomaterials themselves often have interesting properties for biomedicine.

Nanosized cerium dioxide is of interest as a potential component of radiopharmaceuticals, since it has antioxidant properties, which allow it to be used as a radioprotector to minimize the damage of ionizing radiation to healthy organs and tissues. At the same time, in malignant neoplasms, cerium dioxide shows prooxidant properties, which will potentially enhance the therapeutic effect of the administered drug. At the same time, due to pH sensitivity, cerium oxide exhibits vector properties and is able to deliver the drug to the affected tissue.

Thus, the aim of this work is to obtain novel conjugates of cerium oxide nanoparticles with DOTA and azacrown ligand with 6 nitrogen heteroatoms in the macrocycle (ligand L that was previously studied with both bismuth [1] and zinc [2] radionuclides) and to analyze their labelling with Bi-207 and Zn-65, as well as in vitro and in vivo stability.

**Materials and methods:** Conjugation of nanoparticles with bifunctional derivatives of DOTA (SCN-derivative) was carried out after functionalization of the surface of nanoparticles with amino groups with (3-Aminopropyl)triethoxysilane (APTES). The ligand L was conjugated via an unprotected carboxyl group to an amino group on the surface of the nanoparticles by forming an amide bond using the HBTU reagent. The confirmation of surface modification of the nanoparticles at each stage was confirmed by a series of measurements of ζ-potentials at various pH values in the range from 2 to 11 and the determination of the isoelectric point by electrophoretic light scattering. The additionally modified nanoparticles were analyzed by IR and Raman spectrometry. Conjugation efficiency was also assessed using thermogravimetry.

For in vitro tests solutions complexes of conjugates with radionuclides were added to fetal bovine serum and incubated at 37 °C; then at the fixed time points proteins were precipitated by ethanol and the radioactivity of supernatant was measured and compared with the initial sample. For in vivo experiments normal male mice were used; complexes or blank solutions were injected intraperitoneally, and after 1 or 6 h after injection the mice were euthanized, and major organs and tissues were collected and measured by gamma-spectrometry.

**Results:** High labelling yields (more than 90%) for the studied complexes were at metal cations concentrations of 1·10-4–1·10-3 M. Conjugates were stable in serum both with zinc and bismuth radionuclides and didn’t show transchelation of Zn-65 and Bi-207 by proteins. The most stable complexes were studied in vivo and demonstrated high stability in organism. The excretion of these complexes was studied with a metabolic cage and compared to blank samples excretion.

**Conclusion:** Cerium oxide nanoparticles were successfully conjugated with DOTA and azacrown ligand L. Complexes of the conjugates with bismuth and zinc were stable in presence of serum proteins and also were stable in vivo. The results obtained indicate that conjugates of cerium dioxide nanoparticles and azacrown ethers are promising material for study with a view to using them in nuclear medicine.

This work was funded by Russian Science Foundation, project No 21-73-00101, https://rscf.ru/project/21-73-00101/.

**References**

1. Egorova BV, Matazova EV, Mitrofanov AA, Aleshin GYu, Trigub AL, Zubenko AD, Fedorova OA, Fedorov YuV, Kalmykov SN. Novel pyridine-containing azacrown-ethers for the chelation of therapeutic bismuth radioisotopes: complexation study, radiolabeling, serum stability and biodistribution. Nucl Med Biol. 2018;60:1–10.
2. Aleshin GYu, Egorova BV, Priselkova AB, Zamurueva LS, Khabirova SYu, Zubenko AD, Karnoukhova VA, Fedorova OA, Kalmykov SN. Zinc and copper complexes with azacrown ethers and their comparative stability in vitro and in vivo. Dalton Trans 2020;49(19):6249–58.

## OP04

### Dual labeling of [^67^Ga]Ga-DTPA-FITC-silk fibroin nanoparticles for biodistribution studies: optimization of the radiolabeling approach

#### M. Alejandra Asensio Ruiz^1,3^, Angela Alonso Garcia^1^, Luz Bravo Moreno^1^, A. A. Lozano Pérez^2,3^, M. T. Martínez Martínez^1,3^

##### ^1^Unidad de Radiofarmacia. Hospital Clínico Universitario Virgen de la Arrixaca, 30120 Murcia, Spain. ^2^Departamento de Biotecnología, Instituto Murciano de Investigación y Desarrollo Agrario y Alimentario (IMIDA), Murcia, Spain. ^3^Grupo de Radiofarmacia. Instituto Murciano de Investigación Biosanitaria IMIB-Arrixaca. 30120 Murcia, Spain

*EJNMMI Radiopharmacy and Chemistry* 2023; 8(Suppl1): OP04

**Aim:** In the last decades, application of Silk Fibroin Nanoparticles (SFNs) as a carrier of bioactive molecules has notably increased1. In the field of new drug development, image guided technique is playing an increasingly important role in the investigation of the biodistribution and pharmacokinetics. In this sense, the aim of this work is to develop an approach of dual labeled (fluorescent and radioactive) Silk Fibroin Nanoparticles (SFNs) for in vivo SPECT biodistribution studies and ex vivo optical imaging.

**Materials and methods:** 67 Ga chloride was obtained from 67 Ga citrate processing procedure previously described 2. Briefly, ~ 37 MBq of 67 Ga citrate was passed through the SEPPAK silica column at a flow rate of approximately 0.1 mL/min. Then, the column was washed with WFI and finally, 67 Ga was eluted from the column in HCl 0.1 M. The activity, pH and RCP (ITLC-SG in CH_3_COONa 0.4 M, Rf = 0.0–0.1) of 67 Ga chloride eluted were measured.

SFN were conjugated with DTPA and labeled with FITC as previously described3. The procedure for radiolabeling with 67 Ga chloride was optimized by varying either the temperature and time of incubation and the radioisotope/nanoparticle ratio. Radiolabeling was performed by incubation of 500 ul of DTPA-FITC-SFN (0.1, 0.5, 1, 2 and 3.5 mg) with 50 ul of sodium acetate 1.14 M and 500 ul of 67GaCl_3_ (4.5–16.5 MBq), at room temperature or 100 °C during 15 or 30 min. After incubation, suspensions were centrifuged at 14,100×*g* during 30 min, supernatant was discarded and pellet was washed twice with WFI. Radiolabeling efficiency was calculated as the ratio between the radioactivity in the pellet and supernatant.

The hydrodynamic characterization of the nanoparticles was performed by DLS technique and Zaverage (d.nm), PdI and Zpotential (ζ, mV) were determined before and after radiolabeling.

**Results:** The recovery in the form of 67 Ga chloride was of 87.37 ± 3.03% (n = 8) of the initial activity, with pH and RCP under specifications (pH~2 and RCP ≥ 99.9%)

Temperature and time of incubation were fixed at 20 °C and 15 min. Radiolabeling yield was SFN concentration dependent, ranging from 10.36 to 72.2% at 32.85 and 1.29 MBq/mg, respectively (n = 6).

The hydrodynamic characteristics of the nanoparticles remain stable after labeling, with no significative differences in their Zaverage, PdI and Zpotential values.

**Conclusion:** Radiolabeling of 67Ga-DTPA-FITC-SFNs is achieved in mild conditions with a yield of ~ 72% and no modification of their properties for cell penetration.

67Ga-DTPA-FITC-SFNs can be considered as an interesting dual imaging agent for biodistribution and pharmacokinetic applications.

**References**

1. Lozano-Pérez AA, Rodriguez-Nogales A, Ortiz-Cullera V, Algieri F, Garrido-Mesa J, Zorrilla P, et al. Int J Nanomed. 2014;9:4507–20.
2. Scasnár V, van Lier JE. The use of SEP-PAK SI cartridges for the preparation of gallium chloride from the citrate solution. Eur J Nucl Med. 1993;20(3):273.
3. Martínez Martínez T, García Aliaga Á, López-González I, Abella Tarazona A, Ibáñez Ibáñez MJ, Cenis JL, Meseguer-Olmo L, Lozano-Pérez AA. Fluorescent DTPA-silk fibroin nanoparticles radiolabeled with ^111^In: a dual tool for biodistribution and stability studies. ACS Biomater Sci Eng. 2020;6(6):3299–309.

## OP05

### New radiolabeled exendin analogues show reduced renal retention

#### Lieke Joosten^1^, Cathelijne Frielink^1^, Tom Jansen^1^, Daphne Lobeek^1^, Fritz Andreae^2^, Sandra Heskamp^1^, Martin Gotthardt^1^ and Maarten Brom^1^

##### ^1^Department of Medical Imaging, Radboud university medical center, Nijmegen, The Netherlands. ^2^Forschungs- und Entwicklungs GmbH, piCHEM, Parkring 3, 8074 Grambach, Austria

*EJNMMI Radiopharmacy and Chemistry* 2023; 8(Suppl1): OP05

**Aim:** A promising imaging method for detection of insulinomas is targeting the glucagon-like peptide-1 (GLP-1) receptor using PET/CT [1]. However, high accumulation of radiolabeled exendin in the kidneys can hamper the ability to detect small insulinomas in proximity to the kidneys and limits its use as a radiotherapeutic agent [2]. In this study, we developed two novel exendin-analogues for imaging and therapy to reduce renal retention by incorporating a cleavable methionine-isoleucine-linker. We examined the renal retention and insulinoma targeting properties of these new exendin analogues in a nude mouse model bearing subcutaneous GLP-1R-expressing insulinomas.

**Materials and methods:** NOTA or DOTA were conjugated via a methionine-isoleucine-linker to the C-terminus of exendin-4 (NOTA-MI-exendin-4 or DOTA-MI-exendin-4). NOTA- and DOTA-exendin-4 without the linker were used as references. The affinity of the peptides for the GLP-1R was determined in a competitive binding assay using GLP-1R transfected cells. Biodistribution of [^68^Ga]Ga-NOTA-exendin-4, [^68^Ga]Ga-NOTA-MI-exendin-4, [^177^Lu]Lu-DOTA-exendin-4 and [^177^Lu]Lu-DOTA-MI-exendin-4 was performed in INS-1 tumor-bearing BALB/c nude mice and PET/CT was acquired to visualize renal retention and tumor targeting. For all tracers, dosimetric calculations were performed to determine the kidney self-dose.

**Results:** The affinity for the GLP-1 receptor was in the low nanomolar range (< 11 nM) for all peptides. Ex vivo biodistribution revealed a significant lower kidney uptake of [^68^Ga]Ga-NOTA-MI-exendin-4 four hours post injection (34.2 ± 4.2 %ID/g), compared with [^68^Ga]Ga-NOTA-exendin-4 (127.7 ± 9.5 %ID/g). Accumulation of [^68^Ga]Ga-NOTA-MI-exendin-4 in the tumor was 25.0 ± 8.0 4 h p.i. and similar to that of [^68^Ga]Ga-NOTA-exendin-4 (24.9 ± 9.3). PET/CT confirmed the findings in the biodistribution studies. Kidney uptake of [^177^Lu]Lu-DOTA-MI-exendin-4 was 13.0 ± 2.5 %ID/g 72 h p.i., compared with 45.8 ± 3.9 %ID/g for [^177^Lu]Lu-DOTA-exendin-4. Uptake in the tumor was 3.7 ± 1.7 and 6.9 ± 2.9 %ID/g 72 h p.i., for [^177^Lu]Lu-DOTA-MI-exendin-4 and [^177^Lu]Lu-DOTA-exendin-4 respectively. For both compounds with the cleavable linker an improved tumor-to-kidney ratio was found. The estimated absorbed kidney dose was reduced with 12% for the Ga-68-labeled and 58% for the Lu-177-labeled cleavable compounds.

**Conclusion:** Both new exendin analogues with a methionine–isoleucine-linker showed reduced renal retention and improved tumor-to-kidney ratios compared with their reference without linker. Future studies should demonstrate whether [^68^Ga]Ga-NOTA-MI-exendin-4 results in improved detection of small insulinomas in close proximity to the kidneys with PET/CT. [^177^Lu]Lu-DOTA-MI-exendin-4 might open a window of opportunity for exendin-based radionuclide therapy.

**References**

1. Antwi K, Fani M, Nicolas G, et al. Localization of hidden insulinomas with (6)(8)Ga-DOTA-exendin-4 PET/CT: a pilot study. J Nucl Med. 2015;56:1075–8.
2. Luo Y, Yu M, Pan Q, et al. ^68^Ga-NOTA-exendin-4 PET/CT in detection of occult insulinoma and evaluation of physiological uptake. Eur J Nucl Med Mol Imaging. 2015;42:531–2.

## OP06

### Influence of peptide length on the in vitro and in vivo properties of ^177^Lu-labelled gastrin analogues

#### Anton Amadeus Hörmann^1^, Maximilian Klingler^1^, Taraneh Zavvar^1^, Christine Rangger^1^, Christian Mair^1^, Elisabeth von Guggenberg^1^

##### ^1^Department of Nuclear Medicine, Medical University of Innsbruck, Innsbruck, Austria

*EJNMMI Radiopharmacy and Chemistry* 2023; 8(Suppl1): OP06

**Aim:** The use of radiolabelled minigastrin (MG) analogues for targeting CCK2Rs in cancers, especially medullary thyroid cancer (MTC), is limited by rapid proteolytic degradation in vivo. Enzymatic degradation could be significantly reduced by site-specific modifications in the linear peptide's C-terminal receptor-specific domain (1). In this study, the in vitro and in vivo effects of the N-terminal lengthening or shortening of the amino acid sequence DGlu-Ala-Tyr-Gly-Trp-(N-Me)Nle-Asp-1-Nal-NH2 (DOTA-MGS5) were investigated.

**Materials and methods:** Based on human MG and pentagastrin (PG), two MG analogues containing 13 and 7 amino acids with site-specific amino acid substitutions were synthesised by standard Fmoc strategy. The in vitro characterization included determination of changes in hydrophilicity and serum protein binding. Two CCK2R-expressing cell lines were used to evaluate the cell internalisation. Receptor affinity was evaluated in A431-CCK2R cells expressing the human CCK2R. The stability and biodistribution of the ^177^Lu-labeled peptide analogues were evaluated in BALB/c mice. Additional blocking studies with PG demonstrated receptor-specific uptake in CCK2R expressing tissues.

**Results:** The ^177^Lu-labelled gastrin analogues showed high hydrophilicity, while the binding to serum proteins was threefold increased for the MG analogue compared to the PG derivative. A strong affinity for the CCK2R in the low nanomolar range could be verified. For both ^177^Lu-labelled gastrin analogues, high cell internalisation (~ 30 to ~ 50% after 2 h incubation) could be demonstrated. A further enhancement of in vivo stability 10 min p.i. was confirmed for the PG derivative (~ 93% intact radiopeptide), whereas the MG peptide derivative demonstrated reduced stability (~ 69% intact radiopeptide). In BALB/c mice, the radiolabelled peptides showed comparable uptake in the stomach, which was efficiently blocked by co-injection of PG. The MG analogue demonstrated increased uptake in the spleen, liver and kidneys when compared to the PG derivative.

**Conclusion:** A combination of high cell internalisation and a favourable biodistribution profile was confirmed for both radiopeptides. However, lengthening the peptide sequence significantly lowered in vivo stability and increased uptake in renal tissue, while shortening resulted in reduced absorption in the kidneys. Further biodistribution studies will be carried out to evaluate the tumour targeting potential of the new gastrin analogues.

**Reference**

1. Klingler M, Summer D, Rangger C, Haubner R, Foster J, Sosabowski J, Decristoforo C, Virgolini I, von Guggenberg E. DOTA-MGS5, a new cholecystokinin-2 receptor-targeting peptide analog with an optimized targeting profile for theranostic use. J Nucl Med. 2019;60:1010–6.

## OP07

### Enhancing the in vivo stability of somatostatin-based radiotracers with the amide-to-triazole switch methodology

#### X. Guarrochena^1,2,3^, P. Kanellopoulos^4^, A. Stingeder^1,3^, L. Recnik^1,2,3^, I. Feiner^1,2,3^, T. Maina^4^, B. A. Nock^4^, T. L. Mindt^1,2,3^

##### ^1^Ludwig Boltzmann Institute Applied Diagnostics, General Hospital of Vienna, Vienna, Austria. ^2^Department of Inorganic Chemistry, Faculty of Chemistry, University of Vienna, Vienna, Austria. ^3^Department of Biomedical Imaging and Image Guided Therapy, Division of Nuclear Medicine, Medical University of Vienna, Vienna, Austria. ^4^Molecular Radiopharmacy, INRASTES, NCSR “Demokritos”, 15341 Athens, Greece

*EJNMMI Radiopharmacy and Chemistry* 2023; 8(Suppl1): OP07

**Aim:** Somatostatin-based radiopeptides are used in clinics for the diagnosis and therapy of neuroendocrine tumors. DOTA-TATE and DOTA-TOC display high affinity for SST2R whereas DOTA-NOC is a multisomatostatin binding analog (SST2,3,5R). Neither of these radiopeptides interact with all the SSTR subtypes. AT2S is a somatostatin based radioligand that preserves the ability to interact with all the receptor subtypes.^1^ However, the limited in vivo stability prevents its use in clinics. Our group has developed a strategy to stabilize radiopeptides based on the use of 1,4-disubstituted 1,2,3-triazoles as metabolically stable amide bond bioisosteres.^2^ The stabilization of AT2S was attempted with the amide-to-triazole switch methodology. Additionally, the effect of the triazole incorporation on the peptide stability was compared to the use of the Neprilysin endopeptidase inhibitor Entresto®.^3^

**Materials and methods:** The peptide elongation and the triazole insertion were both accomplished on solid phase. The DOTA-conjugated triazolopeptides were labeled with In-111. Competition binding assays were performed to determine the affinity for SST1,2,3,5R. The lead peptide was subjected to in vivo blood stability studies in Swiss albino mice. Preliminary biodistribution experiments and SPECT imaging studies were conducted with HEK-SST2R xenografts. Additional in vivo stability and biodistribution studies were performed posterior to the administration of Entresto®.

**Results:** The synthesis of cyclic triazolopeptides was achieved with purities greater than 95%. The radiochemical purity and yield were over 95%. The lead compound displayed high to moderate nanomolar affinity for all the receptor subtypes. The in vivo stability was twofold higher compared to the reference compound but the administration of Entresto® showed to be more efficient in preserving the integrity of the peptide. The tumor uptake was comparable to the reference compound AT2S but the renal uptake showed to be higher. The use of Entresto® enhanced the tumor uptake.

**Conclusion:** The amide-to-triazole switch methodology has been successfully applied for the first time in the stabilization of a cyclic radiopeptide. This strategy served to obtain somatostatin-based peptidomimetics with preserved in vitro receptor affinity and enhanced in vivo stability. The use of Entresto® has shown to be superior to preserve the integrity of the radiopeptide in blood and resulted in higher tumor uptakes. Different approaches will be evaluated to reduce the renal uptake and future experiments with SST1,3,5R Xenografts are in planning.

**References**

1. Tatsi A, Maina T, Cescato R, Waser B, Krenning EP, de Jong M, Cordopatis P, Reubi JC, Nock BA. EJNMMI Res. 2012;2(1):1.
2. Valverde IE, Bauman A, Kluba CA, Vomstein S, Walter MA, Mindt TL. Angew Chem Int Ed. 2013;52(34):8957–60.
3. Nock BA, Maina T, Krenning EP, de Jong M. J Nucl Med. 2014;55(1):121–7.

## OP08

### [^99m^Tc][Tc(N)(PNP)]-tagging of biomolecules: effects of the introduction of water-soluble substituents on the PNP ligand structure

#### Bolzati C^1^, Salvarese N^1^, Spolaore B^2^, Gobbi C^1^, Ghiani S^3^, Maiocchi A^4^

##### ^1^ICMATE-CNR, Corso Stati Uniti, 4- 35127, Padova, Italy. ^2^Department of Pharmaceutical Sciences, University of Padua, Via Marzolo, 5 - 35131 Padova, Italy. ^3^Bracco Imaging SpA, Bioindustry Park del Canavese, Via Ribes 5 - 10010 Colleretto Giacosa. Torino, Italy. ^4^Bracco S.p.a; Via Caduti di Marcinelle, 13, 20134 Milano, Italy

*EJNMMI Radiopharmacy and Chemistry* 2023; 8(Suppl1): OP08

**Aim:** [^99m^Tc][Tc(N)(PNP)]-system (PNP = alkoxy-alkyl-bis-phosphinoamine) is an interesting platform for the labeling of bioactive molecules derivatized with a chemically accessible cysteine residue [1]. One usage restriction is attributable to the need of heating over extended times to complete the complexation reaction; such conditions are incompatible with sensitive targeting vectors, which required mild reaction conditions and low temperature.

The development of ligand systems that form stable and hydrophilic chelates at room temperature and under mild reaction conditions is of great interest for the conjugation of molecular effectors. In this connection, air-stable and ws-hydroxyalkyl-functionalized phosphines represent an interesting class of coordinating ligands to produce ws-chelate/conjugates in vivo stable and characterized by favorable pharmacokinetic profiles. Our aim is to explore the effects of ws substituents on the reactivity properties of the PNP ligand and its ability to form a reactive ws-[Tc(N)(PNP)]2+-framework [2, 3]. The mainstream bulky PNP ligands are used for the sake of comparison.

**Materials and methods:** Ws *N*,*N*-bis(di-hydroxymethylphosphinoethyl)methoxyethylamine (PNP3OH) was synthesized as phosphonium salt PNP3OH·HCl (*N*,*N*-bis(tri-hydroxymethylphosphoniumethyl)methoxyethylamine-hydrochloride) and fully characterized. Then, the formation of the ws-[^99m^Tc(N)(PNP3OH)]2+-moiety was investigated as well as its reactivity with different coligands characterized by dianionic [S^S]2−, [S^O]2− and monoanionic [S^NH_2_]− sets of donors (YZ) as bifunctional chelating agents (BFCA). Then, the effective applicability of ws-[^99m^Tc(N)(PNP3OH)]2+-synthon to the labeling of protein scaffolds was assessed by choosing apomyoglobin (apoMb) as a model protein, which was previously derivatized via site-specific enzymatic reaction catalyzed by transglutaminase with the H-Cys-Gly-Lys-Gly-OH tetra-peptide (H3Cys~apoMb) for insertion in the protein sequence of a reactive N-terminal Cys for ^99m^Tc chelation. Labelings were performed in physiological conditions at room temperature within 30 min. The stability and biological behavior of selected [^99m^Tc][Tc(N)(PNP3OH)]-tagged compounds were studied.

**Results:** The insertion of water-soluble pendant groups on the P atoms of PNP does not affect its coordination properties versus the [^99m^Tc][Tc(N)]-core allowing the formation of the corresponding [^99m^Tc(N)(PNP3OH)]2+-synthon. Rather, such modification changes the physical–chemical properties of the building block promoting its water solubility and improving the reactivity toward cysteine-based and dithiolate-containing BFCA. Radiosyntheses were performed efficiently under physiological conditions at room temperature within 30 min using a low BFCA concentration (10-6 M).[^99m^Tc(N)(PNP3OH)(YZ)]+/0 complexes show good in vitro and in vivo stability, desirable clearance from the non-target tissues and excretion mainly through the kidneys. Notably, rapid labeling and site specificity are preserved when the synthon was reacted with H3Cys~apoMb model.

**Conclusion:** Conclusions. Incorporation of bioactive molecules into a ws-[^99m^Tc(N)(PNP)]-based mixed compound is feasible through reproducible, highly specific, and quantitative reactions, with no evidence of by-products. In a wider perspective, this supports the application of the [^99m^Tc(N)(PNP)]-technology to the labeling of temperature-sensitive biomolecules for SPECT imaging.

**Acknowledgment**

Associazione Italiana per la Ricerca sul Cancro: AIRC, IG 2020 ID 24528 and Bracco Imaging SpA

**References**

1. Bolzati C, Dolmella A. Nitrido technetium-99 m core in radiopharmaceutical applications: four decades of research. Inorganics. 2020;8:3. https://doi.org/10.3390/inorganics8010003.
2. Bolzati C, Ghiani S, Maiocchi A, Refosco F, Salvarese N, Spolaore B. Method for labeling of sensitive and thermosensitive targeting biomolecules with technetium based compounds. 2018.
3. Bolzati C, Salvarese N, Spolaore B, Vittadini A, Forrer D, Brunello S, Ghiani S, Maiocchi A. Water-soluble [Tc(N)(PNP)] moiety for room-temperature ^99m^Tc labeling of sensitive target vectors. Mol Pharm. 2022. https://doi.org/10.1021/acs.molpharmaceut.1c00816.

## OP11

### Benzoazacrown chelator BATA as a better candidate than DOTA for TAT with Bi(III) and Ac(III)

#### Ekaterina V. Matazova^1^, Bayirta V. Egorova^1^, Anna V. Pashanova^2^, Anastasia D. Zubenko^2^, Artem A. Mitrofanov^1^, Olga A. Fedorova^2,3^, Stanislav V. Ermolaev^4^, Aleksandr N. Vasiliev^1,4^, Stepan N. Kalmykova^1^

##### ^1^Lomonosov Moscow State University, 119991 Leninskie Gory, 1/3, Moscow, Russian Federation. ^2^A. N. Nesmeyanov Institute of Organoelement Compounds of Russian Academy of Sciences, 119991 Vavilova, 28, GSP-1, Moscow, Russian Federation. ^3^Mendeleev University of Chemistry and Technology of Russia, 125047 Miusskaya sqr., 9, Moscow, Russian Federation. ^4^Institute for Nuclear Research of Russian Academy of Sciences, 117312 60th October Anniversary Prospect, 7a, Moscow, Russian Federation

*EJNMMI Radiopharmacy and Chemistry* 2023; 8(Suppl1): OP11

**Aim:** Alpha-emitting radionuclides are increasingly being considered as part of the novel radiopharmaceuticals due to their much higher linear energy transfer (LET) and hence more cytotoxicity than β-emitters [1]. Ac-225 and Bi-213 are among the most commonly used radionuclides for TAT: preparations based on them are already undergoing phase II clinical trials [2]. Although the commonly used DOTA-based conjugates with peptides has already shown great results, the quantitative binding of the metal cation with heat-sensitive targeting biomolecules remains a challenge. Generally, the complexation with DOTA requires heating up to 100 °C, which complicates preparation procedure [3]. Moreover, some in vivo instability of the AcDOTA was discovered which supposed to be due to the thermodynamic preference of DOTA for smaller ions than the largest trivalent ion in periodic table [4]. Our aim was conducting the detailed research from thermodynamic stability of Bi(III) and Ac(III) complexes with new benzoazacrown ligand BATA together with kinetic study to biodistribution in normal mice.

**Materials and methods:** Stability constants were determined by potentiometric titration as well as FISRE method (free-ion selective radiotracer extraction) with HDEHP (di-(2-ethylhexyl)phosphoric acid). Radiolabelling was conducted with Bi-207 and Ac-228 radioisotopes of Bi(III) and Ac(III) in mQ water and radiochemical yield was determined by TLC (thin layer chromatography) on aluminum-backed TLC plates (cellulose, Sigma-Aldrich) and the eluent 0.9% NaCl–0.01 M NaOH. Kinetic study of acid-assisted dissociation and formation of BiBATA was conducted by UV absorption spectroscopy in I = 0.6 M (H,K)Cl to prevent hydroxides and colloid formation of the free Bi(III). Complex stability in fetal bovine serum was determined by precipitation of serum peptide and measuring radioactivity of supernatant by gamma-spectroscopy. Biodistribution study was conducted with Bi-207 and Ac-225 labelled complexes in accordance with EU Directive 2010/63/EU for animal experiments and metabolic chamber was used in case of AcBATA complex to collect excrements for further measurements.

**Results:** The absolute values of the stability constants of BiBATA forms determined by potentiometric titration were very high, so the FISRE method was used to obtain reliable values. We used the same method for the complex with Ac(III) as well as potentiometric titration with Ac(III) lanthanide analogue La(III). Obtained stability constants of BATA complexes were higher than those of DOTA indicating a great affinity of Bi(III) and Ac(III) to BATA. Radiolabelling of BATA occurred immediately at room temperature moreover BATA was labeled with Ac(III) at a lower ligand concentration than DOTA. A comparison was made of the formation and dissociation rates of the BiBATA complex with the BiDOTA and BiDTPA complexes in an acidic medium. Under these conditions, BiBATA and BiDTPA complexes formed quickly, while the complex of Bi(III) with DOTA did not form at all. The dissociation of the BiBATA occurred at pH < 1, while at pH 2 and above the complex was stable which is explained by the different stability of diprotonated and monoprotonated species. The mechanism of acid-assisted dissociation of Bi(III) complex with BATA consist in fast protonation of a coordinated carboxylate group in the first step, then the transfer of this proton from the carboxylate group to a nitrogen atom of macrocycle which is the rate-determining step and finally the dissociation of the double-N-protonated complex. Such inspiring results prompted us to study the stability in media with competing cations and blood serum. BATA labelled with Bi-207 and Ac-228 demonstrated stability in vitro at least for 2 days in case of Bi(III) and at least for 1 day in case of Ac(III). Hence biodistribution of BATA complexes in normal mice was studied 1 and 6 h after administration of the Bi(III) complex and 6 h after administration of the Ac(III) complex. The BiBATA complex had a very similar biodistribution profile and was eliminated from the body just as quickly as the complex BiDOTA. The organ with the highest Bi(III) accumulation was kidneys and % ID/g was the same for BiBATA and BiDOTA complexes. Almost all AcBATA was cleared from the body after 6 h via urinary excretion and the accumulation in each organ did not exceeded 0.5% ID/g indicating very high stability in vivo.

**Conclusion:** In course of our research of Bi(III) and Ac(III) complexes with BATA we obtained high thermodynamic stability constants as well as high stability of the complex in vivo. High stability in vivo of BiBATA is associated with the high kinetic inertness of mono- and deprotonated forms of the complex. In addition BATA was found to be superior to DOTA for the rapid chelation of Bi(III) and Ac(III) due to the less rigid structure of the 18-crown-6. Taken together it seems that the 18-crown-6 macrocycle is more preferable for such large cations as Bi(III) and Ac(III).

**Acknowledgment**

This work was supported by Russian Science Foundation grant No 18-73-10035

**References**

1. Graf F, Fahrer J, Maus S, Morgenstern A, Bruchertseifer F, Venkatachalam S, Fottner C, Weber MM, Huelsenbeck J, Schreckenberger M, Kaina B, Miederer M. PLoS ONE. 2014;9(2):e88239.
2. Sollini M, Marzo K, Chiti A, Kirienko M. Rendiconti Lincei. Scienze Fisiche e Naturali. 2020;31:231–47.
3. Essler M, Gärtner FC, Neff F, Blechert B, Senekowitsch-Schmidtke R, Bruchertseifer F, Morgenstern A, Seidl C. EJNMMI 2012;39:602–12.
4. Deal KA, Davis IA, Mirzadeh S, Kennel SJ, Brechbiel MW. J Med Chem. 1999;42:2988–99.

## OP12

### Preclinical evaluation of [^68^Ga]Ga-DATA5m.SA.FAPi: a room temperature gallium-68 labeled radioligand for FAP targeting

#### Alondra Escudero-Castellanos^1^, Surachet Imlimthan^1^, Elena Menéndez^1^, Eirinaios Pilatis^1^, Euy Sung Moon^2^, Tilman Läppchen^1^, Hendrik Rathke^1^, Ali Afshar-Oromieh^1^, Frank Rösch^2^, Axel Rominger^1^, Eleni Gourni^1^

##### ^1^Department of Nuclear Medicine, Inselspital, Bern University Hospital, University of Bern, Bern, Switzerland. ^2^Department of Chemistry—TRIGA site, Johannes Gutenberg—University of Mainz, Germany

*EJNMMI Radiopharmacy and Chemistry* 2023; 8(Suppl1): OP12

**Aim:** Tumor stroma consists the largest portion of the total tumor mass (over 90%) [1] and it has been shown that the activated stroma undertakes critical roles in cell invasion, extravasation, migration, angiogenesis, immune evasion and therapeutic resistance. Cancer-Associated Fibroblasts (CAFs), a crucial component of tumor stroma, count approximately 80% of all fibroblasts in tumor microenvironment [2]. CAFs are identified by several biomarkers with Fibroblast Activation Protein (FAP), a type II transmembrane protein, being one of them. FAP is highly expressed on the surface of CAFs and promotes tumor growth invasion, metastasis and immunosuppression and its inhibition may increase the antitumor biological response [3–4]. Furthermore, it appears to be a promising target in the field of nuclear medicine, with the potential to be used for non-invasive radionuclide-based in vivo tumor imaging and therapy.

The aim of the present study was the preclinical evaluation of a FAP-based inhibitor, which has the advantage to be labeled with gallium-68 at room temperature (RT) with the potential to be used for diagnostic applications of FAP positive tumors.

**Materials and methods:** The FAP inhibitor UAMC1110 was functionalized with the chelator 5-[1,4-bis tertbutoxycarbonylmethyl-6-(tert-butoxycarbonylmethyl-methyl-amino)-[1,4] diazepan-6-yl]-pentanoic acid (DATA5m-3tBu) via the squaric acid-based spacer. The generated precursor DATA5m.SA.FAPi was labeled with gallium-68 at RT, using the Modular-Lab PharmTracer module by Eckert and Ziegler. The quality control of the radiotracer and its stability over time as well as in human serum was verified by RP-HPLC. The lipophilicity and the tendency of [^68^Ga]Ga-DATA5m.SA.FAPi to bind to human plasma proteins was also investigated. [^68^Ga]Ga-DATA5m.SA.FAPi was further evaluated in vitro (saturation and internalization binding studies) using the human hTERT PF179T CAF cells which overexpress FAP as proven by western blot and immunofluorescence staining. Furthermore, biosdistribution as well PET/CT imaging studies were performed in U87MG (TM1) and PC3 (TM2) tumor xenografts.

**Results:** DATA5m.SA.FAPi was labelled with gallium-68 at RT in 98% radiochemical purity with the apparent molar activity (Am) ranging between 16 and 22 GBq/µmol. The stability 4 h (4 h after labeling) and metabolic stability (30 min after incubation with human serum) revealed no change in the chromatograms patterns. The binding of the radiotracer to human proteins was about 10% after 30 min of incubation. The radiotracer exhibited a rather hydrophilic profile with a logDoctanol/PBS of − 3.6 ± 0.1. [^68^Ga]Ga-DATA5m.SA.FAPi showed high affinity for the FAP positive CAF cells, with Kd = 0.92 ± 0.23 nM and Bmax = 0.92 ± 0.23 nM. The calculated number of receptors per cell was found to be approximately 540,000. Specific internalization was found in cell culture with 93% of the total cell associated activity has been internalized after 1 h of incubation with the cells. Biodistribution studies in both tumor models showed high and specific accumulation at 3 h p.i. in tumor (TM1 = 7.5 ± 3.0, TM2 = 4.7 ± 0.3% IA/g), bone (TM1 = 6.1 ± 1.2, TM2 = 5.7 ± 0.3% IA/g), pancreas (TM1 = 6.9 ± 1.0, TM2 = 3.7 ± 0.6% IA/g) and blood (TM1 = 5.0 ± 0.7, TM2 = 3.9 ± 0.6% IA/g). PET/CT studies illustrate the results from the biosdistribution studies.

**Conclusion:** DATA5m.SA.FAPi can efficiently be labeled at RT with gallium-68, leading to a radiotracer with high affinity towards FAP. The ^68^Ga-labeled conjugate is taken up specifically by the stromal cells exhibiting fast and high internalization rate already at early time points. The results of the in vivo studies in two different models provide evidence for the potential use of [^68^Ga]Ga-DATA5m.SA.FAPi as a diagnostic tracer for non-invasive imaging of FAP positive tumors.

**References**

1. Huber MA, Schubert RD, et al. J Investig Dermatol. 2003;120(2):182–8.
2. Smith NR, Baker D, et al. Clin Cancer Res. 2013;19(24):6943–56.
3. Moon ES, Elvas F, et al. EJNMMI Radiopharm Chem. 2020;5(19):1–20.
4. Jansen K, Heirbaut L, et al. J Med Chem. 2014;57(7):3053–74.

## OP13

### 3p-C-NETA-TATE: a theranostic somatostatin analogue targeting neuroendocrine tumors

#### S. Ahenkorah^1^ E.Murce^2^, C. Cawthorne^3^, Yann Seimbille^2^, Thomas Cardinaels^4^, C. C. Deroose^5^, G. Bormans^1^, M. Ooms^6^, Frederik Cleeren^1^

##### ^1^Radiopharmaceutical Research, Department of Pharmacy and Pharmacology, KU Leuven, Leuven. ^2^Department of Radiology and Nuclear Medicine, Erasmus MC, Rotterdam, The Netherlands. ^3^Nuclear Medicine and Molecular Imaging, Department of Imaging and Pathology, KU Leuven, Leuven, Belgium. ^4^Institute for Nuclear Materials Science, Belgian Nuclear Research Center (SCK CEN), Mol, Belgium. ^5^Nuclear Medicine Unit, University Hospitals Leuven, Leuven, Belgium. ^6^NURA research group, Belgian Nuclear Research Center (SCK CEN), Mol, Belgium

*EJNMMI Radiopharmacy and Chemistry* 2023; 8(Suppl1): OP13

**Aim:** Radiolabeled somatostatin analogues such as [^68^Ga]Ga-DOTATATE and [^177^Lu]Lu-DOTATATE have become important tools for diagnosis and treatment of patients with neuroendocrine tumors. [^18^F]AlF-NOTA-octreotide, a promising ^18^F-labeled somatostatin analogue and potential alternative for ^68^Ga-DOTA-peptides, is currently under clinical evaluation [1]. Ideally, the same precursor (combination of chelator-linker-vector) should be used for production of both diagnostic and therapeutic radioprobes to ensure very similar (e.g. Al^18^F/^213^Bi/^177^Lu) or identical (e.g. complementary Tb-radionuclides) pharmacokinetic properties, allowing for accurate personalised dosimetry estimation, and radionuclide therapy of NET patients. 3p-C-NETA is a versatile chelator that can be used for both diagnostic and therapeutic applications [2, 3]. We have recently reported promising results for [^18^F]AlF-3p-C-NETA-TATE [2] and here we present the first results of radiosynthesis and in vitro evaluation of [^213^Bi]Bi-3p-C-NETA-TATE and [^177^Lu]Lu-3p-C-NETA-TATE.

**Materials and methods:** 3p-C-NETA-TATE was radiolabeled with ^177^Lu (NaOAc, 0.1 M, pH 4.1, 12 min) or ^213^Bi (Tris-HCl, 4 M, pH 8.5, 7 min) at 40 or 95 °C. The in vitro stability of the corresponding radiocomplexes was determined in PBS and human serum at 37 °C. In vitro cell binding and internalization was performed with [^213^Bi]Bi-3p-C-NETA-TATE (185 kBq/well; Am: 1.23 GBq/µmol) or [^177^Lu]Lu-3p-C-NETA-TATE (53 kBq/well; 176.66 Am: GBq/µmol) using SSTR2 expressing cells (BON-1.SSTR2) [4]. Cell viability (104 cells/well) and clonogenic assay (103 cells/well) was performed with [^213^Bi]Bi-3p-C-NETA-TATE using the same cell line.

**Results:** 3p-C-NETA-TATE efficiently sequestered ^177^Lu (RCC 95%) and ^213^Bi (RCC 90%) at 12 and 7 min respectively at 40 °C. [^177^Lu]Lu-3p-C-NETA-TATE showed excellent in vitro stability in both PBS and mouse serum (90% intact complex at day 3). Starting with 94.3% radiochemical purity, [^213^Bi]Bi-3p-C-NETA-TATE demonstrated good stability (90% intact radiocomplex) after 5 h in both PBS and human serum. High SSTR2 specific cell binding and internalization (11.4 ± 0.7% of which 62.1% is internalized) was observed after 60 min incubation for [^177^Lu]Lu-3p-C-NETA-TATE whereas only 3.3 ± 0.5% cell binding (of which 39.2% is internalized) was observed for [^213^Bi]Bi-3p-C-NETA-TATE, probably due to blocking effects because of low apparent molar activity. 99% blocking after co-incubation with 100 µM octreotide was observed for both tracers at 60 min. Reduced viability of BON-1.SSTR2 was observed after 48 h incubation with [^213^Bi]Bi-3p-C-NETA-TATE (9.9 ± 0.9% viability at 0.1 MBq activity). Also, only 0.1 MBq activity of [^213^Bi]Bi-3p-C-NETA-TATE was required to achieve cell surviving fraction of 0.1 (SF0.1).

**Conclusion:** 3p-C-NETA-TATE is an excellent and versatile agent that can be used for both targeted radionuclide therapy (^177^Lu, ^213^Bi) and diagnostic applications (Al^18^F) and has advantages as an alternative to the DOTA analogues in current clinical use. [^18^F]AlF-3p-C-NETA-TATE and [^213^Bi]Bi-3p-C-NETA-TATE/[^177^Lu]Lu-3p-C-NETA-TATE will be further evaluated as potential theranostic pairs in SSTR2 expressing tumor mice.

**References**

1. Eur J Nucl Med Mol Imaging. 2020;47(13):3033–46.
2. Theranostics. 2022;12(13):5971–85.
3. Bioorg Med Chem Lett. 2008;18(11):3436–9.
4. Front Endocrinol. 2018;9(APR).

## OP15

### Benzoazacrown ligands for a chelation of copper and lead radioisotopes and comparative stability of their complexes in vitro and in vivo

#### L. Zamurueva^1^, B. Egorova^1^, A. Zubenko^2^, A. Pashanova^2^

##### ^1^Lomonosov Moscow State University, Leninskie Gory, 119991, 1, Moscow, Russia. ^2^A.N.Nesmeyanov Institute of Organoelement Compounds of Russian Academy of Sciences, Vavilova St. 28, 119991, Moscow, Russia

*EJNMMI Radiopharmacy and Chemistry* 2023; 8(Suppl1): OP15

**Aim:** Nowadays many studies in radiopharmacy are devoted to copper radioisotopes 67Cu and 64Cu [1], which can be used for theranostics, and 212Pb isotope, which can be applied in radiopharmaceuticals as an in vivo generator of short-lived therapeutic alpha-emitter 212Bi [2]. These radionuclides bind to biological vectors using bifunctional ligands. Macrocyclic H4DOTA and its analogs are widely used as such ligands in radiopharmaceuticals. Radiolabeling of H4DOTA with copper and lead cations requires heating. In our study complexes of lead and copper cations with new benzoazacrown ethers possessing varied number of chelating groups, including variation of azacrown-cavity size. Complexation with these ligands occurred instantly at room temperature.

**Materials and methods:** The ligand protonation constants and stability constants of complexes were determined by potentiometric titration. The method of spectrophotometric titration in the visible and UV regions was used to refine the stoichiometry of formed complexes. To radiolabel the complex, 64Cu was isolated from the proton irradiated nickel target and the long-lived lead isotope 210Pb—from the solution of parent 226Ra with its daughters. Thin layer chromatography followed by autoradiography and gamma spectrometry was used to control the bound fraction of radionuclide. The stability of the complexes was studied in biologically relevant media (solutions of microelements, isotonic solution of NaCl and ninefold excess of serum proteins). To analyze the stability of the most effective copper complex in vivo, a biodistribution of complex in mice was studied in comparison with copper blank solution.

**Results:** Complexes with a tetraacetate ligand have the highest constants (logβ(CuL) = 24.8, logβ(PbL) = 21.6). Furthermore tetraacetate (L1) and tetrapicolinate (L3) ligands can form binuclear complexes with Cu2+ (logβ(Cu2L1) = 34.4 and logβ(Cu2L3) = 33.7). All complexes with lead isotopes were stable in presence of blood serum excess during the studied time range (2 days). However, the possible release of 212Bi after decay of 212Pb chelated by these benzoazacrowns still remains to be resolved. For copper isotopes, the complex with the highest constant turned out to be the least effective: after 2 h, in the presence of an excess of serum, only about 20% of copper remained in the composition of the complex. The complex of copper with a ligand with 3 acetate groups and a smaller cavity was slightly more stable: after 2 h, about half of the copper complex remained intacted. Finally, the complex with a ligand with 4 picolinate groups was stable in the presence of an excess of serum: 100% copper remained in the complex over the studied time range (1 day). According to biodistribution, an increased content of the complex in the urine was observed compared to the blank experiment in 1 h post injection. The total content of the radionuclide detected in mice was 1.5 times lower, when it was injected as a complex, in comparison with the blank experiment, the accumulation of radioisotope in the liver was 2 times less (7.3% D/g for CuL and 13.1% D/g for Cu2+) after 6 h.

**Conclusion:** All the studied ligands form stable complexes with lead cations and are suitable for further studies with the 212Pb/212Bi generator pair. For copper, only the ligand with 4 picolinate groups proved to be stable under in vitro conditions. This copper complex is excreted from the body of mice faster than copper blank solution (CuCl2), however, after 6 h accumulation is still observed in organs (but less than in the blank experiment), which may indicate a partial dissociation of the complex in vivo.

**Acknowledgements**

This study is supported by Russian Science Foundation №18-73-10035.

**References**

1. Ahmedova A, Todorov B, Burdzhiev N, Goze C. Copper radiopharmaceuticals for theranostic applications. Eur J Med Chem. 2018;157:1406–25.
2. Kokov KV, et al. Pb : production approaches and targeted therapy applications. 2022, pp. 122.

## OP16

### Can we profit from the auger electron emission of terbium-161 using somatostatin receptor antagonists?

#### Cristina Müller^1,5^, Sarah D. Busslinger^1^, Francesca Borgna^1^, Ulli Köster^2^, Jan Rijn Zeevart^3^, Pascal V. Grundler^1^, Nicholas P. van der Meulen^1,4^, Roger Schibli^1,5^

##### ^1^Center for Radiopharmaceutical Sciences ETH-PSI, Paul Scherrer Institute, Villigen-PSI, Switzerland. ^2^Institut Laue-Langevin, Grenoble, France. ^3^Necsa, Pelindaba, South Africa. ^4^Laboratory of Radiochemistry, Paul Scherrer Institute, Villigen-PSI, Switzerland. ^5^Department of Chemistry and Applied Biosciences, ETH Zurich, Zurich, Switzerland

*EJNMMI Radiopharmacy and Chemistry* 2023; 8(Suppl1): OP16

**Aim:** Terbium-161 has similar decay properties as lutetium-177 but co-emits a substantial amount of Auger electrons [1]. It was commonly believed that Auger electrons are only effective when localized in close proximity to the tumor cell nucleus. However, it has been recently demonstrated by our group that DOTA-LM3, a non-internalizing somatostatin receptor (SSTR) antagonist, benefited more from the co-emission of Auger electrons from terbium-161 than the internalizing SSTR agonist DOTATOC [2]. It proofed, however, difficult to draw an unambiguous conclusion due to the large difference in the total cell uptake for this agonist/antagonist pair. The aim of this study was to reproduce the data using radiolabeled DOTATATE, an alternative SSTR agonist, with potentially similar cell uptake as DOTA-LM3.

**Materials and methods:** SST analogues were radiolabeled with terbium-161 and lutetium-177 at molar activities up to 100 MBq/nmol, with a radiochemical purity 99%. Uptake and internalization experiments using SSTR-expressing AR42J tumor cells were performed to determine the membrane-bound and internalized fraction of the radiopeptides. The ^161^Tb-labeled DOTATATE and DOTA-LM3 were investigated in vitro by AR42J cell viability assays and the effects compared to those of their respective ^177^Lu-labeled counterparts. Preclinical therapy studies were performed using ^161^Tb- and ^177^Lu-labeled DOTATATE (2 × 10 MBq/mouse) for comparison with the data obtained using radiolabeled DOTA-LM3.

**Results:** Terbium-161 and lutetium-177 were interchangeable without affecting the SSTR-binding properties of the radiopeptides. [161Tb]Tb-/[^177^Lu]Lu-DOTATATE showed 64 ± 10% uptake on AR42J cells which was much higher than previously published for [161Tb]Tb-/[^177^Lu]Lu-DOTATOC (16 ± 2%) and in the same range as for [^161^Tb]Tb-/[^177^Lu]Lu-DOTA-LM3 (69 ± 6%) [2]. Radiolabeled DOTATATE was rapidly internalized with ~90% of bound radiopeptide localized in the cytoplasm, while ~ 90% of radiolabeled DOTA-LM3 was localized at the cell membrane. Terbium-161 was more effective to reduce cell viability in vitro than lutetium-177 with all of the SST analogues. The shift in the EC50 value was, however, more pronounced with DOTA-LM3 (102×) than with DOTATATE (36×). Therapy studies revealed the most favorable therapeutic effect when using [^161^Tb]Tb-DOTA-LM3, with 6/6 mice still alive at the end of the study on Day 49, while the least effect was observed with [^177^Lu]Lu-DOTATATE resulting in a median survival time of only 21.5 days.

**Conclusion:** With this study, we confirmed our previous observation that membrane-localizing peptides profit more from Auger electron emission than those that are effectively internalized into the cytoplasm. Based on the similar in vitro uptake of DOTATATE and DOTA-LM3, they present a better pair to study the therapeutic potential of Auger electrons depending on their cellular localization. [^161^Tb]Tb-DOTA-LM3 was most effective to reduce cell viability in vitro and tumor growth in vivo among all investigated radiopeptides whereas [^177^Lu]Lu-DOTATATE was the least efficacious.

**References**

1. Lehenberger et al. Nucl Med Biol. 2011;38:917.
2. Borgna et al. Eur J Nucl Med Mol Imaging. 2022;49:1113.

## OP17

### Comparative study on ^64^Cu production on variable energy cyclotrons TR-19 (ACSI)/KIUBE (IBA) using solid and liquid targets

#### Diana Cocioabă^1,3^, Radu Leonte^1^, Alexandra Fonseca^2^, Bogdan Burghelea^1^, Roxana Cornoiu^1,4^, Livia Chilug^1^, Antero Abruhnosa^2^ and Dana Niculae^1^

##### ^1^Horia Hulubei National Institute for Physics and Nuclear Engineering (IFIN-HH), Radiopharmaceutical Research Centre, Magurele, Romania. ^2^Institute for Nuclear Sciences Applied to Health, University of Coimbra (ICNAS/UC), Portugal. ^3^University of Bucharest, Doctoral School of Physics, Faculty of Physics, Bucharest, Romania. ^4^University Politehnica of Bucharest, Doctoral School of Applied Chemistry and Materials Science, Faculty of Chemical Engineering and Biotechnologies, Bucharest, Romania

*EJNMMI Radiopharmacy and Chemistry* 2023; 8(Suppl1): OP17

**Aim:** Copper is involved in many biochemical processes, such as the formation of red blood cells, intestinal absorption of iron and accelerating its release from the liver, maintaining the integrity of vascular walls and the heart and stimulating neurons. It has antioxidant and anti-inflammatory effects and increases the body's resistance to infections. Five of its 27 radioisotopes, namely ^60^Cu (T1/2 = 23.7 min), ^61^Cu (T1/2 = 3.333 h), ^62^Cu (T1/2 = 9.673 min), ^64^Cu (T1/2 = 12.7 h) and ^67^Cu (T1/2 = 61.83 h), can be produced in cyclotron, having desired characteristics for medical applications. 64Cu is of a particular interest for both targeted therapy and follow-up imaging also due to its simultaneous emissions of Auger electrons, β+ (17.86%) and β− (39.03%) particles. The aim of the study was to compare the solid and liquid target routes of Cu-64 production processes in variable energy cyclotrons.

**Materials and methods:** Solid Target: The process involves enriched 64Ni electrodeposition on a platinum support attached to a shuttle, resulting in a compact disk, without stalagmites or cracks. The target was irradiated at TR-19 cyclotron (ACSI, Canada), via ^64^Ni(p,n)^64^Cu reaction. After irradiation, the target is dissolved in an automatic module (Taddeo Synthesis Module, ALCEO System, COMECER, Italy) by using 6M hydrochloric acid and negative voltage at 90° temperature. The resulted solution automatically switches to the purification module, where an ion exchange resin (AG1X8) was employed, eluted with HCl solutions of different concentrations.

Liquid target: The process involves dissolving enriched 64Ni in 10 mM nitric acid as liquid target. The solution was irradiated at KIUBE Cyclone cyclotron (IBA, Belgium) via the same nuclear reaction (^64^Ni(p,n)^64^Cu). After irradiation, the solution is automatically sent to the purification module (IBA Synthera® Extension Module). Two process-specific resins were used to remove impurities.

The radiolabeling of DOTA-NT(8-13) was performed comparatively with Cu-64 chloride solutions, prepared on the two routes. Quality control of the ^64^Cu-DOTA-NT(8-13) was performed by TLC and HPLC, in addition to radionuclide purity and identity analyses at different steps of the processes. ^64^Cu-DOTA-NT(8-13) prepared at different molar activities were tested for stability and the binding profiles were assayed using colon cancer (HT29 and HCT116) cell lines using Ligand Tracer technique.

**Results:** Irradiation of the solid target yielded an activity of 15.42 ± 1.85 GBq at end of bombardment with a beam current of 25 μA and irradiation time of 6 h, and an additional 2 h of post-processing. Liquid target process yielded an activity of 3 GBq by a beam current of 50 μA, irradiation time of 3.5 h and shorter processing time. The radiochemical purity of the radiolabelled peptide, assesed by radio-TLC and radio-HPLC, was higher than 99% after purification regardless the irradiation process. The stability and binding profiles of ^64^Cu-DOTA-NT(8-13) tested in the same conditions on the colon cancer cell lines were not significantly different.

**Conclusion:** Both routes, using liquid and solid targets respectively, for Cu-64 production are optimized for using with variable energy cyclotrons and post-processed accordingly. There are some practical considerations and technical limitations to be taken into account for best results; they are presented and discussed in detail. Molar activity, yield, processing time, target preparation, radionuclide impurities, dose to operators and practical aspects are all factors to be weigh carefully when deciding for one or the other of the production routes, without affecting the quality of the final radiopharmaceutical product.

**References**

1. Niculae D, Dusman R, Leonte RA, Chilug LE, Dragoi CM, Nicolae A, Serban RM, Niculae DA, Dumitrescu IB, Draganescu D. Biological pathways as substantiation of the use of copper radioisotopes in cancer theranostics. Front Phys. 2021;8. https://doi.org/10.3389/fphy.2020.568296.
2. Fonseca AI, Alves VH, do Carmo SJC, Silva M, Hrynchak I, Alves F, Falcão A, Abrunhosa AJ. Production of GMP-compliant clinical amounts of copper-61 radiopharmaceuticals from liquid targets. Pharmaceuticals 2022;15(6):723. https://doi.org/10.3390/ph15060723.
3. Palma E, Raposinho P, Cabral Campello MP, Belo D, Guerreiro JF, Alves V, Fonseca A, Abrunhosa AJ, Paulo A, Mendes F. Anticancer activity and mode of action of copper(II)-bis(thiosemicarbazonato) complexes with pendant nitrogen heterocycles. Eur J Inorg Chem. 2021. https://doi.org/10.1002/ejic.202100168.

## OP18

### natCr(p,x) or natV(α,x)? Dosimetric assessments at comparison for high-purity ^52g^Mn PET tracer production

#### Barbaro F^1,2^, Canton L^1^, Carante M P^2,3^, Colombi A^2,3^, De Nardo L^1,4^, Fontana A^3^, Meléndez-Alafort L^5^

##### ^1^INFN, Sezione di Padova, Padova, Italy. ^2^Dipartimento di Fisica dell’Università di Pavia, Pavia, Italy. ^3^INFN, Sezione di Pavia, Pavia, Italy. ^4^Dipartimento di Fisica e Astronomia dell’Università di Padova, Padova, Italy. ^5^Istituto Oncologico Veneto IOV IRCCS, Padova, Italy

*EJNMMI Radiopharmacy and Chemistry* 2023; 8(Suppl1): OP18

**Aim:** ^52g^Mn appears as promising tracer for positron emission tomography (PET) thanks to its decay properties (β+ = 29.4%, E(β+) avg = 242 keV) and its quite long half-life (t1/2 = 5.6 day) [1]. Potential nuclear-medicine applications in imaging require a sufficiently quantity and high quality production in compliance with the European Pharmacopoeia requirements. Focus of this work is to develop precise simulations and models to compare the standard ^nat^Cr(p,x)^52g^Mn production route and the alternative ^nat^V(α,x)^52g^Mn one here proposed [2, 3]. To this aim the radionuclidic purity and dose increase, due to the co-produced radioactive contaminants, have been evaluated.

**Materials and methods:** The nuclear code Talys has been employed to optimize the ^nat^V(α,x)^52g^Mn cross section by tuning the parameters of the microscopic level densities [4]. Thick-target yields have been calculated from the expression of the rates as energy convolution of cross sections and stopping powers, and finally integrating over the time evolution of the relevant decay chains. Dosimetric evaluations have been accomplished by means of the OLINDA software considering the injection of [^xx^Mn]Cl^2^ in female and male phantoms [5, 6]. Finally, the dose increase has been calculated by combining the yield of xxMn radioisotopes estimated for both reactions with the dosimetric outcomes.

**Results:** Good agreement was obtained between cross sections calculations and measurements. With the natV(α,x) route, the dose increase shows a less harmful impact on patients’ health due to a reduced contamination by other Mn radioisotopes.

**Conclusion:** Both natV(α,x) and natCr(p,x) reactions are suitable for a clinically acceptable production of ^52g^Mn. If we consider a thick-target production (200 µm), the Vanadium target requires a α beam with 48 MeV, while the Chromium target implies a 17 MeV proton beam. Compared to natCr(p,x)52gMn, the natV(α,x)52gMn reaction produces larger quantity of the PET tracer, a longer time with radionuclidic purity higher than 99%, and finally, considering the injection of the [^52g^Mn]Cl_2_ compound, a systematically lower dose increase.

**References**

1. Wooten AL, Aweda TA, Lewis BC, Gross RB and Lapi SE. Biodistribution and PET Imaging of pharmacokinetics of manganese in mice using Manganese-52. PLoS ONE. 2017;12(3).
2. Colombi A, Carante MP, Barbaro F, Canton L, Fontana A. Production of high-purity 52gMn from natV targets with α beams at cyclotrons. Nucl Technol. 2022;208:735–52 (2022). https://doi.org/10.1080/00295450.2021.1947122.
3. Barbaro F, Canton L, Carante MP, Colombi A, De Nardo L, Fontana A, Meléndez-Alafort L. The innovative 52gMn for PET imaging: production cross section modeling and dosimetric evaluation. https://arxiv.org/abs/2204.00402.
4. Goriely S, Hilaire S, Koning AJ. Improved predictions of nuclear reaction rates with the TALYS reaction code for astrophysical applications. A&A. 2008;487:767.
5. Stabin MG, Sparks RB, Crowe E. OLINDA/EXM: the second-generation personal computer software for internal dose assessment in nuclear medicine. J Nucl Med. 2005;46:1023–7.
6. De Nardo L, Ferro-Flores G, Bolzati C, Esposito J, Melèndez-Alafort L. Radiation effective dose assessment of [51Mn]- and [52Mn]-chloride. Appl Radiat Isot. 2019;153:108805.

## OP19

### Production and separation of the PET-radionuclide Ti-45 from a liquid nat-Sc target for ligand complexation

#### Unni Augestad Kvitastein^1^, Chubina Pathma Kumarananthan^1^, Nikolai Golten Fiskeseth^1^ & Tom Christian Holm Adamsen^1,2^

##### ^1^Department of Chemistry, University of Bergen, 5020 BERGEN, Norway. ^2^Centre for Nuclear Medicine, Department of Radiology, Haukeland University Hospital, 5021 BERGEN, Norway

*EJNMMI Radiopharmacy and Chemistry* 2023; 8(Suppl1): OP19

**Aim:** The most common PET-radionuclides are unsuitable for imaging of some physiological processes due to their short (e.g. C-11, Ga-68) or long half-lives (e.g. Zr-89, Cu-64). They either result in insufficient imaging or excessive radiation exposure. Titanium-45 is a promising PET-radionuclide with a half-life of 3.08 h. It has favorable decay characteristics for PET-imaging (85% positron decay) and has previously been produced in a cyclotron via the Sc-45(p,n)Ti-45 reaction by using a solid target [1, 2]. The aim of this study is to optimize the production of Ti-45 using a liquid target. Then isolate Ti-45 from the target material and other impurities formed during irradiation, using solid phase extraction (SPE) and liquid–liquid extraction (LLE) for further complexations with ligands. This to form a foundation for later development of radiopharmaceuticals labeled with Ti-45 for use in PET imaging.

**Materials and methods:** The liquid target was prepared by dissolving Sc(NO_3_)_3_·3H_2_O in HNO_3_. Using a PET Trace 860 cyclotron equipped with a PETtrace 800 ^68^Ga Liquid target, different concentrations (1.0–2.5 M) Sc(NO_3_)_3_ were irradiated with a 14.3 MeV proton beam for 60–180 min with a beam-current of 20–30 μA. In the SPE approach the cyclotron product was loaded onto a ZR-resin, then the Sc-species were washed out with HCl, and finally Ti-45 was eluted using a ligand. This method was also automated using the FASTlabTM 2 synthesizer. In the LLE approach a mixture of guaiacol/anisole was used to extract Ti-45 from the aqueous phase into the organic phase. The phases were separated using a centrifuge and isolated. The organic phase was then mixed with different ligand solutions for complexation. The cyclotron product and the separation fractions were analyzed with gamma-ray spectrometry and the formation of the complexes was confirmed with radio-HPLC. A full factorial design was used to optimize the Ti-45 activity.

**Results:** Gamma-ray spectrometry revealed EOB activities of Ti-45 ranging from 0.40 to 1.17 GBq in the cyclotron product. Co-production of radionuclidic impurities, i.e. Sc-44, Sc-44 and Mo-93m were found, and trace amounts of Ti-44 were also detected. The radionuclidic purity of the cyclotron product ranged from 85.5 to 99.3 %. Gamma-ray spectrometry of each separation fraction from the SPEs indicates impurities being washed out with HCl, and most of Ti-45 being eluted out by the ligand solution. For the LLEs the gamma-ray data show higher activity of Ti-45 in the organic phase, compared to the aqueous phase. Radio-HPLC of the [Ti-45]-ligand complexes shows peaks with retention times close to the retention times of cold reference Ti-ligand complexes.

**Conclusion:** The productions resulted in Ti-45 activities ranging from 0.40 to 1.17 GBq (EOB) with a radionuclidic purity between 85.5% and 99.3%. Radionuclidic impurities, i.e, Sc-44, Sc-44m and Mo-93m were found, including trace amounts of Ti-44. Using lower currents and irradiation times yielded lower amounts of impurities. A model of the irradiation parameters and Ti-45 activities reveals that a combination of low current and irradiation time, and high concentration of both Sc(NO_3_)_3_ and HNO_3_ yields the highest Ti-45 activities. The complexation of Ti-45 with two different ligands was successfully achieved, utilizing both SPE and LLE for isolation of Ti-45. The SPE was also successfully automated by using the FASTlabTM 2 synthesizer.

Further work includes isolating and refining the Ti-45 radiotracer from the complexation process through the use of semi-preparative HPLC. It is also necessary to study the biodistribution of the Ti-45 radionuclide. This is planned in two steps: (1) Ti-45 radiotracer distribution itself in healthy mice (2) biodistribution of a Ti-45 labeled biomolecule in a relevant cancer model.

**References**

1. Chaple IF, Thiele K, Thaggard G, Fernandez S, Boros E, Lapi SE. Optimized methods for production and purification of Titanium-45. Appl Radiat Isot 2020;166:109398.
2. Kuhn S, Spahn I, Scholten B, Coenen HH, Positron and γ-ray intensities in the decay of 45Ti. Radiochimica Acta 2015;103(6):403–9.

## OP20

### Second generation Al^18^F-labeled D-amino acid based peptide for CXCR4 targeted molecular imaging

#### Muriel Spahn^1^, Kaat Luyten^1^, Tom Van Loy^2^, Christopher Cawthorne^3^, Christophe M. Deroose^3^, Dominique Schols^2^, Guy Bormans^1^ and Frederik Cleeren^1^

##### ^1^Laboratory for Radiopharmaceutical Research, Department of Pharmaceutical and Pharmacological Sciences, KU Leuven; Leuven, Belgium. ^2^Laboratory of Virology and Chemotherapy, Department of Microbiology, Immunology and Transplantation, KU Leuven; Leuven, Belgium. ^3^Nuclear Medicine and Molecular Imaging, Department of Imaging and Pathology, KU Leuven; Leuven, Belgium

*EJNMMI Radiopharmacy and Chemistry* 2023; 8(Suppl1): OP20

**Aim:** CXCR4 PET imaging with [^68^Ga]Pentixafor has been shown to have intrinsic diagnostic value [1, 2]. However, development of a radiopharmaceutical labelled with fluorine-18 would be of benefit due its favorable characteristics compared to gallium-68. Therefore, the aim of this study was to develop an alternative CXCR4-targeting scaffold with high CXCR4 binding affinity based on viral macrophage inflammatory protein II-derived CXCR4 antagonist and composed entirely of d-amino acids [3], which conserves high binding to CXCR4 when labelled with fluorine-18. The first generation ligand [^18^F]AlF-NOTA-DV1-k-(DV3) was created by combining DV1 (first 21 aa of vMIP-II) with DV3 (first ten aa of vMIP-II) via a lysine bridge and the second generation ligand consisted of two linked DV1 arms with the purpose of increasing the affinity towards CXCR4, resulting in [^18^F]AlF-NOTA-2xDV1(c11sc12s).

**Materials and methods:** [^18^F]AlF labeling of both D-peptides was done in a GMP compliant, fully automated Trasis AllinOne® module. [^68^Ga]Ga-Pentixafor was produced in an automated SCINTOMCIS GRP module. The radiochemical purity was assessed with radioHPLC using a reversed phase PRP-1 column coupled to an UV detector and a NaI(Tl) scintillation detector. The CXCR4 affinity was acquired in a competitive assay by incubating CXCR4 overexpressing Jurkat cells with the natAlF-D-peptides and the fluorescently labeled endogenous CXCR4 ligand CXCL12AF647. Cell binding assays were conducted on u87.MG, u87.CD4, u87.CD4.CXCR4, u87.CD4.CXCR7, u87.CD4.CCR5 and u87.CD4.CCR7 cells and the target specificity was assessed by exposing the ligand to an excess of small molecule receptor competitors (75 µM), namely 4,4′-diisothiocyanatostilbene-2,2′-disulfonic acid (CD4 antagonist), AMD3100 (CXCR4 antagonist), Maraviroc (CCR5 antagonist), VUF11207 (CXCR7 agonist), or (2×)DV1(c11sc12s) (self-block). The in vivo profile was analyzed in u87.CXCR4 tumor xenografted mice using µPET/CT and an ex vivo biodistribution study at 75 min. p.i..

**Results:** [^18^F]AlF-NOTA-(2x)DV1(c11sc12s) was successfully labeled with a RCY of 41.1 ± 15.5% (n = 3), a RCP of 99.4 ± 0.2% (n = 3) and an apparent molecular activity of 19.61 ± 3.5 MBq/nmol (n = 3). The IC50 values were 5.3 ± 0.9 nM, 1.3 ± 0.2 nM and 8.6 ± 1.1 nM for AlF-NOTA-DV1-k-(DV3), AlF-NOTA-(2×)DV1(c11sc12s) or [natGa]Ga-Pentixafor, respectively.

The affinity towards CXCR4 was assessed in cell binding assays and flow cytometry was used to confirm the selective overexpression of receptors, as well as the simultaneous absence of CXCR4 in cell lines other than u87.CD4.CXCR4. The total-bound fraction of [^18^F]AlF-NOTA-(2×)DV1(c11sc12s) in u87.CXCR4 cells was the highest compared to [^68^Ga]Ga-Pentixafor and [^18^F]AlF-NOTA-DV1-k-(DV3) (12.2 ± 0.5% vs. 8.9 ± 0.3% vs. 6.0 ± 0.1% respectively). In u87.MG, u87.CD4, u87.CD4.CXCR4, u87.CD4.CCR5, u87.CD4.CCR7 and u87.CD4.CXCR7 cells the total-bound fraction of [^18^F]AlF-NOTA-(2×)DV1(c11sc12s) was 23.3 ± 1.1%, 22.4 ± 0.3%, 32.4 ± 1.8%, 19.7 ± 0.8%, 24.4 ± 1.0% or 25.2 ± 1.2%, respectively. In comparison, the total-bound fraction of [^68^Ga]Ga-Pentixafor on u87.CD4, u87.CD4.CCR5 and u87.CD4.CXCR4 cells was 1.4 ± 0.1%, 1.3 ± 0.1% or 3.2 ± 0.1%, respectively. Contrary to expectations, the uptake of [^18^F]AlF-NOTA-(2×)DV1(c11sc12s) was not completely blocked in u87.CD4.CXCR4 cells exposed to AMD3100 (ligand uptake reduction by 82.5%). The same observation was made in the other cell lines, where the uptake was reduced by 20.1%, 19.4% and 53.3% in u87.CD4, u87.CD4.CCR5 or u87.CD4.CXCR7 cells, respectively, when exposed to the corresponding receptor competitor. Adding AMD3100 to the aforementioned receptor competitors further decreased the uptake by 79.8%, 62.3% and 79.7% in u87.CD4, u87.CD4.CCR5 or u87.CD4.CXCR7 cells, respectively. The strongest uptake inhibition was measured by adding (2×)DV1(c11sc12s), resulting in 99.1% and 93.8% blockage on u87.CXCR4 or u87.CD4 cells, respectively, indicating non CXCR4-specific uptake.

In u87.CXCR4 tumor xenografted SCID mice, both Al^18^F-tracers demonstrated an elevated accumulation in the liver, spleen and bone marrow, however, the tumor uptake remained comparably low. The co-injection of AMD3100 (5 mg/kg i.v.) showed a reduction in the uptake in all these organs (up to 94% in liver), including the xenografted u87.CXCR4 tumor [4].

**Conclusion:** The second generation ligand [^18^F]AlF-NOTA-2xDV1(c11sc12s) has been successfully labeled and demonstrated a high uptake in u87.MG cells and u87 cells overexpressing CD4, CXCR4, CCR5, CCR7 and CXCR7. The inability to fully block the ligand from binding onto u87.CD4.CXCR4 cells and the similar uptake profile in cells overexpressing CD4, CXCR7, CCR5 or CCR7 suggests a lack of selectivity for CXCR4. This was also mirrored in the in vivo assays, where the strong hepatic uptake was blockable using AMD3100, since AMD3100 has been reported to antagonize CXCR4 heterodimers as well. Therefore additional cell lines overexpressing potential targets are required in order to fully understand the versatile binding profile of [^18^F]AlF-NOTA-2xDV1(c11sc12s) [5].

**References**

1. Kraus S, et al. (^68^)Ga-pentixafor PET/CT for detection of chemokine receptor CXCR4 expression in myeloproliferative neoplasms (in Eng). J Nucl Med. 2022;63(1):96–9. https://doi.org/10.2967/jnumed.121.262206.
2. Osl T, Schmidt A, Schwaiger M, Schottelius M, Wester HJ. A new class of PentixaFor- and PentixaTher-based theranostic agents with enhanced CXCR4-targeting efficiency (in Eng). Theranostics. 2020;10(18):8264–80. https://doi.org/10.7150/thno.45537.
3. Demoin DW, et al. Synthesis and evaluation of an (18)F-labeled pyrimidine-pyridine amine for targeting CXCR4 receptors in gliomas (in Eng). Nucl Med Biol. 2016;43(10):606–11. https://doi.org/10.1016/j.nucmedbio.2016.05.005.
4. Luyten K, et al. D-peptide-based probe for CXCR4-Targeted molecular imaging and radionuclide therapy (in Eng). Pharmaceutics. 2021;13(10). https://doi.org/10.3390/pharmaceutics13101619.
5. Kalatskaya I, Berchiche YA, Gravel S, Limberg BJ, Rosenbaum JS, Heveker N. AMD3100 is a CXCR7 ligand with allosteric agonist properties. Mol Pharmacol. 2009;75(5):1240. https://doi.org/10.1124/mol.108.053389.

## OP21

### Preclinical testing and automated synthesis of [^177^Lu]-labelled DOTA-MGS5 for application in CCK2R-targeted therapy

#### Taraneh Sadat Zavvar^1^, Anton A. Hörmann^1^, Maximilian Klingler^1^, Christian Mair^1^, Lieke Joosten^2^, Gerben Franssen^2^, Peter Laverman^2^ Elisabeth von Guggenberg^1^

##### ^1^Department of Nuclear Medicine, Medical University of Innsbruck, 6020 Innsbruck, Austria. ^2^Department of Medical Imaging, Radboud University Medical Center, 6525 Nijmegen, The Netherlands

*EJNMMI Radiopharmacy and Chemistry* 2023; 8(Suppl1): OP21

**Aim:** DOTA-MGS5 is a novel minigastrin (MG) analogue with improved tumour-to-kidney ratio and optimised in vivo stability which can be considered a promising new candidate for the theranostic use in neoplasms that express the cholecystokinin-2 receptor (CCK2R). This receptor is overexpressed in a variety of cancers, in particular medullary thyroid carcinoma. With the goal of the clinical translation of [^177^Lu]Lu-DOTA-MGS5 for peptide receptor radionuclide therapy (PRRT), we performed specific preclinical testing and established the automated synthesis process.

**Materials and methods:** A431-CCK2R cells stably transfected with human CCK2R and AR42J cells expressing rat CCK2R were used to investigate the receptor-specific cell internalization of [^177^Lu]Lu-DOTA-MGS5. Biodistribution studies in A431-CCK2R xenografted female BALB/c nude mice were conducted up to 7 days post-injection. The validation of the radiolabelling process was carried out using a Modular-Lab PharmTracer synthesis module (Eckert and Ziegler, Berlin, Germany), DOTA-MGS5 in GMP quality and no-carrier added [^177^Lu]LuCl3 produced from highly enriched ytterbium-176. Product specifications and analytical procedures were defined according to European Pharmacopoeia monographs available for other radiopharmaceuticals. For DOTA-MGS5, a toxicity study in Wistar rats was carried out in a GLP-compliant laboratory.

**Results:** [^177^Lu]Lu-DOTA-MGS5 showed a high cell uptake of 68.0 ± 2.3% in A431 cells and 48.6 ± 2.2% in AR42J cells 4 h after incubation. The radiolabelled peptide demonstrated a favourable biodistribution profile in mice with a low non-specific accumulation of radioactivity in most of the tissues. Somewhat prolonged retention of radioactivity was observed in the CCK2R-expressing stomach. The tumour uptake in A431-CCK2R xenografts was remarkably high, with values of 68.1 ± 10.0% IA/g for the first time point of 1 h p.i. studied and dropped to 28.9 ± 7.2% and 12.6 ± 3.3% IA/g 1 and 3 days after injection, respectively. Based on the pharmacokinetic data obtained in mice, dosimetry estimates for humans were calculated. The cassette-based preparation of [^177^Lu]Lu-DOTA-MGS5 was accomplished within 45 min. A radiochemical purity of > 95% could be achieved up to 4 h post preparation. Based on the performed toxicity study, a maximum dose of 100 µg was defined for the first therapeutic use.

**Conclusion:** Favourable tumour uptake and tumour retention were confirmed for [^177^Lu]Lu-DOTA-MGS5 in A431-CCK2R xenografted mice. The performed preclinical testing and automation of synthesis support the clinical translation of [^177^Lu]Lu-DOTA-MGS5 in patients with advanced medullary thyroid carcinoma and other CCK2R expressing tumours.

**References**

1. Klingler M, Summer D, Rangger C, Haubner R, Foster J, Sosabowski J, et al. DOTA-MGS5, a new cholecystokinin-2 receptor-targeting peptide analog with an optimized targeting profile for theranostic use. J Nucl Med. 2019;60(7):1010–6.
2. Hörmann AA, Klingler M, Rangger C, Mair C, Decristoforo C, Uprimny C, et al. Radiopharmaceutical formulation and preclinical testing of ^68^Ga-labeled DOTA-MGS5 for the regulatory approval of a first exploratory clinical trial. Pharmaceuticals. 2021;14(6):575.

## OP22

### Synthesis and evaluation of two ^18^F-labeled Lys-ureido-Aad derivatives for imaging prostate-specific membrane antigen expression with positron emission tomography

#### Kelly Lu^1^, Chengcheng Zhang^1^, Zhengxing Zhang^1^, Helen Merkens^1^, Ruiyan Tan^1^, Nadine Colpo^2^, François Bénard^1,2,3^, Kuo-Shyan Lin^1,2,3^

##### ^1^Department of Molecular Oncology, BC Cancer, Vancouver, BC V5Z 1L3, Canada. ^2^Department of Functional Imaging, BC Cancer, Vancouver, BC, V5Z 4E6, Canada. ^3^Department of Radiology, University of British Columbia, Vancouver, BC V5Z 1M9, Canada

*EJNMMI Radiopharmacy and Chemistry* 2023; 8(Suppl1): OP22

**Aim:** Majority of prostate cancer is marked by an overexpression of prostate-specific membrane antigen (PSMA) which serves as an excellent target for both diagnostic and therapy due to its large extracellular domain. Both radiolabeled antibody and small molecules have been identified to target PSMA; in particular, the small molecule lysine-ureido-glutamate (Lys-ureido-Glu) motif, which binds to PSMA active site glutamate carboxypeptidase II (NAALADase), has served as the pharmacophore for the recently FDA-approved PSMA-targeting [^18^F]DCFPyL for imaging prostate cancer with positron emission tomography (PET). While there are many reported Lys-ureido-Glu-based PSMA tracers, problems such as high salivary gland and kidney uptake exist. Our project aims to optimize the pharmacokinetic and pharmacodynamics profiles of ^18^F-labelled PSMA tracers for diagnosis of prostate cancer and cancer metastasis. Our work here presents the synthesis and evaluation of two novel lysine-ureido-α-aminoadipic acid (Lys-ureido-Aad)-based PSMA tracers labeled with ^18^F via nucleophilic substitution or isotopic exchange reaction.

**Materials and methods:** KL01040 (nitrilotriacetic acid-Lys(6-fluoronicotinic acid)-tranexamic acid-9-anthrylalanine-Lys-ureido-Aad), its 6-trimethylammonium precursor and KL01007 (nitrilotriacetic acid-Lys(2-azidoacetic acid)-tranexamic acid-9-anthrylalanine-Lys-ureido-Aad) were constructed on solid phase and purified by HPLC after cleavage and deprotection. KL01007 was further clicked with N-propargyl-para-pyridiniumtrifluoroborate to yield KL01007-pyridine-BF3. [^18^F]KL01040 was prepared by nucleophilic substitution of its 6-trimethylammonium precursor with [^18^F]fluoride in DMF at 70 °C using tetrabutylammonium bicarbonate as the base. [^18^F]KL01007-pyridine-BF3 was prepared by ^18^F-19F isotopic exchange in pyridazine solution (pH = 2) incubated at 80 °C. PET/CT imaging and biodistribution studies were conducted at 1 h post-injection in mice bearing PSMA-expressing LNCaP prostate tumor xenografts.

**Results:** KL01040, its 6-trimethylammonium precursor, KL01007 and KL01007-pyridine-BF3 were synthesized in 35.0, 32.3, 84.1 and 3.7% yield, respectively. [^18^F]KL01040 and [^18^F]KL01007-pyridine-BF3 were prepared in 25 and 2% decay-corrected radiochemical yield, respectively. PET imaging showed clear visualization of LNCaP tumor xenografts by [^18^F]KL01040 and [^18^F]KL01007-pyridine-BF3 and both tracers were excreted mainly via the renal pathway. The tumor uptake values of [^18^F]KL01040, [^18^F]KL01007-pyridine-BF3 and [^18^F]DCFPyL were comparable: 11.2 ± 2.06, 11.2 ± 3.44 and 10.3 ± 1.28 %ID/g, respectively. The uptake values in spleen, liver, kidneys and salivary glands were 0.83 ± 0.41, 0.58 ± 0.15, 33.2 ± 10.1 and 0.79 ± 0.11 %ID/g, respectively for [^18^F]KL01040, 0.35 ± 0.20, 0.24 ± 0.08, 7.78 ± 2.15 and 0.23 ± 0.07 %ID/g, respectively for [^18^F]KL01007-pyridine-BF3, and 2.42 ± 1.11, 1.83 ± 0.12, 118 ± 12.6 and 1.27 ± 0.36 %ID/g, respectively for [^18^F]DCFPyL. The tumor-to-muscle, tumor-to-blood, tumor-to-kidney and tumor-to-liver uptake ratios were 38.9 ± 9.40, 5.78 ± 1.13, 0.36 ± 0.09 and 19.8 ± 3.90, respectively for [^18^F]KL01040, 99.7 ± 46.6, 15.1 ± 6.25, 1.54 ± 0.77 and 48.5 ± 15.9 %ID/g, respectively for [^18^F]KL01007-pyridine-BF3, and 39.1 ± 13.1, 16.9 ± 2.93, 0.09 ± 0.02 and 5.69 ± 0.94 %ID/g, respectively for [^18^F]DCFPyL.

**Conclusion:** Deriving from the Lys-ureido-Aad pharmacophore renders both [^18^F]KL01040 and [^18^F]KL01007-pyridine-BF3 significantly lower uptake in most normal organs/tissues compared to those of [^18^F]DCFPyL derived from the Lys-ureido-Glu pharmacophore. This coupled with their comparable tumor uptake results in higher tumor-to-background contrast for [^18^F]KL01040 and [^18^F]KL01007-pyridine-BF3 than [^18^F]DCFPyL. Both [^18^F]KL01040 and [^18^F]KL01007-pyridine-BF3 are promising for imaging PSMA expression with PET and Lys-ureido-Aad is a potential pharmacophore for further optimization of PSMA-targeting imaging and radiotherapeutic agents for characterization and treatment of angiogenesis-related diseases and PSMA-expressing cancers.

**References**

1. Silver DA, et al. Prostate-specific membrane antigen expression in normal and malignant human tissues. Clin Cancer Res. 1997;3(1):81–5.
2. Kozikowski AP, et al. Design of remarkably simple, yet potent urea-based inhibitors of glutamate carboxypeptidase II (NAALADase). J Med Chem. 2001;44(3):298–301.

## OP23

### Synthesis and biological evaluation of chelator-based small molecule PET-radiotracers for imaging of PD-L1

#### Fabian Krutzek^1^, Cornelius K. Donat^1^, Martin Ullrich^1^, Klaus Kopka^1,2,3^, Sven Stadlbauer^1^

##### ^1^Helmholtz-Zentrum Dresden-Rossendorf, Bautzner Landstraße 400, 01328 Dresden, Germany. ^2^Technische Universität Dresden, School of Science, Faculty of Chemistry and Food Chemistry, Mommsenstrasse 4, 01062 Dresden, Germany. ^3^National Center for Tumor Diseases (NCT), Partner Site Dresden, University Cancer Center (UCC), 01307 Dresden, Germany

*EJNMMI Radiopharmacy and Chemistry* 2023; 8(Suppl1): OP23

**Aim:** The programmed cell death ligand 1 (PD-L1) is overexpressed by various cancers, resulting in a downregulation of the local immune response and therefore enabling further tumor growth [1]. Immune checkpoint inhibitors (ICIs) can reactivate the immune system, however, only 30% of the patients respond to an ICI monotherapy [2]. Since PD-L1 is heterogeneously expressed within and across tumor sites, there is an urgent clinical need for non-invasive diagnostic tools to support the therapeutic decision process. Small molecule-based radiotracers for noninvasive molecular PD-L1 imaging offer improved tissue penetration, fast blood clearance and low immunogenicity over radiolabeled antibodies.

**Materials and methods:** Based on a published small molecule PD-L1 inhibitor, 10 different radioligands were synthesized and radiolabeled with copper-64 (HZDR, 30 MeV TR-FLEX cyclotron). Binding affinities were determined on PC3 cells stably overexpressing human PD-L1. For in vivo evaluation, qualitative PET/CT imaging (nanoSCAN PET/CT, Mediso) was performed in NMRI-FoxN1-nude mice bearing PC3-hPD-L1 xenografted tumors.

**Results:** Modification of the PD-L1 binding motif with strongly water-solubilizing sulfonate and phosphonate groups, different linker units and a NODAGA-chelator in 21–25 organic synthesis steps (12–13 longest linear sequence) yielded 10 different ligands [3]. The 64Cu-labelled radiotracers exhibited logD values between − 3.17 and − 4.15 for six ligands of the first series, with dissociation constants (Kd) between 80.5 and 532.8 nM, as determined by saturation binding assays. Depending on the number and pattern of sulfonate and phosphonate groups, the in vivo experiments showed drastically different pharmacokinetic profiles: Compounds bearing a less hydrophilic linker showed improved tumor uptake. Three sulfonates resulted in increased blood circulation times of up to 24 h due to albumin binding, increased renal clearance but also low tumor uptake (SUVmax = 1.4). Substitution of one sulfonate with a phosphonate reduced the circulation time to 2 h, however, accompanied by mainly hepatobiliary clearance. To achieve predominant renal clearance, a second series of four compounds bearing two phosphonate and one sulfonate groups in the solubilizer unit and larger halogens at the central aryl core for improved tumor uptake were synthesized and are currently tested in vivo.

**Conclusion:** Sulfonate groups in the PD-L1 tracers increased circulation times along with renal clearance, while tracers with one phosphonate group reduced the blood circulation time but lead to a more hepatobiliary clearance. Structural modifications to increase the binding affinity and improve tumor uptake are currently ongoing.

**References**

1. Postow MA, Callahan MK, Wolchok JD. J Clin Oncol. 2015;33:1974–82.
2. Topalian SL, Drake CG, Pardoll DM. Cancer Cell. 2015;27:450–61.
3. Stadlbauer S, Krutzek F, Kopka K. EP21212444.0. December 6th 2021.

## OP25

### Development of ACE-selective radioligands as molecular tools to investigate the imbalance of the renin-angiotensin-aldosterone system during Covid-19

#### C. Vaccarin^1^; D. Beyer^1^; P. V. Grundler^1^; N. P. van der Meulen^1,2^; R. Schibli^1,3^; C. Müller^1,3^

##### ^1^Center for Radiopharmaceutical Sciences ETH-PSI-USZ, Paul Scherrer Institute, Villigen-PSI, Switzerland. ^2^Laboratory of Radiochemistry, Paul Scherrer Institute, 5232 Villigen, Switzerland. ^3^Department of Chemistry and Applied Biosciences, ETH Zurich, Zurich, Switzerland

*EJNMMI Radiopharmacy and Chemistry* 2023; 8(Suppl1): OP25

**Aim:** The renin-angiotensin-aldosterone system (RAAS) plays a crucial role in maintaining cardiovascular homeostasis, which is frequently compromised during Covid-19 disease progression [1]. It is hypothesized that ACE and ACE2, two primary components of RAAS, play a significant role in this context [2]. Recent studies suggest that, following the virus infection, an imbalance favoring the ACE functional expression may be responsible for the onset or worsening of Covid-19 symptoms [3]. In this project, we aimed to develop radioligands for the Positron Emission Tomography (PET) imaging of the ACE expression and dynamics starting from known enzyme-selective inhibitors (e.g. BPP9a and lisinopril). Such tools may aid researchers and physicians in following the currently-unclear ACE/ACE2 expression interplay, granting a better understanding of the pathological processes involved in Covid-19 and other relevant cardiovascular pathologies.

**Materials and methods:** The BPP9a peptide and the small molecule lisinopril were selected as ACE-targeting molecules, which were conjugated to an aliphatic linker connected to a DOTA macrocyclic chelator. The ligands’ syntheses were performed by solid-phase peptide synthesis. The chemical identity and purity of the final compounds were assessed by HRMS and HPLC analyses, respectively. The resultant DOTA-BPP9a and DOTA-LIS were labeled with gallium-67 (as a longer-lived surrogate of gallium-68). The final radioligands were evaluated in vitro regarding their ACE affinity and selectivity using ACE- and ACE2-positive HEK cells. Biodistribution and SPECT imaging studies were carried out in HEK-ACE/HEK-ACE2 xenograft-bearing nude mice.

**Results:** DOTA-BPP9a and DOTA-LIS were obtained at 19% and 20% yield, respectively, with a chemical purity > 98%. [67Ga]Ga-DOTA-BPP9a and [67Ga]Ga-DOTA-LIS showed high uptake in ACE-HEK cells (15.4 ± 1.6% and 56.2 ± 0.6%, respectively) and selectivity for the ACE enzyme, with no significant uptake in ACE2-expressing cells (< 0.1%). In biodistribution studies, [67Ga]Ga-DOTA-BPP9a and [67Ga]Ga-DOTA-LIS accumulated in HEK-ACE xenografts (3.1 ± 0.3% IA/g and 16.2 ± 1.1%), but no uptake was observed in HEK-ACE2 xenografts (< 0.5% IA/g) at 3 h post-injection (p.i.). Accumulation in other tissues and organs was negligible for both radioligands (< 1.0% IA/g). The only exception was found to be the kidneys, where [67Ga]Ga-DOTA-BPP9a showed significantly higher retention (3.2 ± 0.8% IA/g) compared to [67Ga]Ga-DOTA-LIS (1.1 ± 0.2% IA/g). The superior biodistribution profile of [67Ga]Ga-DOTA-LIS was also confirmed by SPECT-CT imaging at 1 h, 3 h, and 24 h p.i.

**Conclusion:** The data in this work showed that the small-molecule-based [67Ga]Ga-DOTA-LIS outperformed the peptide-based [67Ga]Ga-DOTA-BPP9a as a valid molecular tool for ACE imaging. [67Ga]Ga-DOTA-LIS was characterized by higher uptake in HEK-ACE xenografts and lower renal retention compared to [67Ga]Ga-DOTA-BPP9a. These characteristics make [67Ga]Ga-DOTA-LIS a favorable candidate for further preclinical investigations towards translation to a clinical setting, and for evaluating ACE expression dynamics during Covid-19 disease progression.

**References**

1. Amraei R, et al. COVID-19, renin-angiotensin system and endothelial dysfunction. Cells. 2020;9(7):1652.
2. Zamorano CN, et al. ACE2: Evidence of role as entry receptor for SARS-CoV-2 and implications in comorbidities. Elife. 2020;9:e61390.
3. Rysz S. et al. COVID-19 pathophysiology may be driven by an imbalance in the renin-angiotensin-aldosterone system. Nat Commun. 2021;12(1):2417.

## OP27

### Preliminary results of the Lip-Re1 phase 1 trial: pharmacokinetics study of ^188^Re-SSS/Lipiodol for HCC treatment

#### C. Bouvry^1^, V. Ardisson^1^, S. Laffont^1^, X. Palard-Novello ^1,2^, C. Bertrand^1^, E. Garin^1,3^, N. Lepareur^1,3^

##### ^1^Centre Eugene Marquis, Rennes, France. ^2^LTSI, INSERM UMR 1099, Rennes, France. ^3^Institut Numecan, INSERM UMR-S 1241, Rennes, France

*EJNMMI Radiopharmacy and Chemistry* 2023; 8(Suppl1): OP27

**Aim:** Hepatocellular carcinoma (HCC), the major primary liver cancer, is a major cause of death, and its incidence is increasing. It often appears on an underlying disease and is usually detected late, with a curative treatment which therefore can only be proposed to a small minority of patients. Taking advantage of the dual blood supply and rich vasculature of the liver, transarterial radioembolisation (TARE) with radiolabeled Lipiodol has demonstrated its interest for the management of HCCs at intermediate to advanced stages [1]. The aim of this study was to evaluate biodistribution and pharmacokinetics of ^188^Re-SSS/Lipiodol [2] after intra-arterial injection for palliative treatment of HCC.

**Materials and methods:** A Phase 1 clinical study was initiated for the treatment of patients suffering from inoperable HCC. Inclusion criteria were measurable tumour, uni- or multinodular, occupying less than 50% of hepatic volume, BCLC stages A–C (or CLIP 0–4), without portal vein thrombosis, after escape, intolerance or contraindication to Sorafenib. 6 patients were treated at first dose-step (1581 ± 414 MBq) and 6 at step 2 (3331 ± 472 MBq) by intra-arterial injection of ^188^Re-SSS/Lipiodol via a transfemoral catheter. Pharmacokinetics data were obtained by collecting blood samples at 1, 6, 12, 24, 48 and 72 h, urinary samples at 24, 48 et 72 h, and faeces samples at 72 h, and with 4 or 5 scintigraphies (SPECT/CT) over a period of 72 h.

**Results:** A mean peak of 1.90 ± 0.67% of injected activity is observed in serum at 12 h then decreasing quickly. Elimination is mainly through urines, remaining very low with 0.97 ± 0.59% of injected activity over 72 h. Digestive elimination is quasi non-existent. TLC analyses of serum and urine samples indicate metabolites are essentially hydrophilic. Scintigraphies show an important, and stable in time, hepatic fixation (79.40 ± 8.87%), with a very good tumour targeting (58.15 ± 13.59%). Only notable extrahepatic fixation is in the lungs, which thus represent main organ at risk.

**Conclusion:** These preliminary results demonstrate exceptional properties of ^188^Re-SSS/Lipiodol, with good tumour targeting and excellent stability in vivo. Tolerance and treatment response are also encouraging [3]. Completion of this dose-escalation study should enable to determine maximum tolerable dose for this new promising treatment.

**References**

1. Bouvry C, Palard X, Edeline J, Ardisson V, Loyer P, Garin E, Lepareur N. Transarterial radioembolization (TARE) agents beyond 90Y-microspheres. BioMed Res Int. 2018;Article ID 1435302:14. https://doi.org/10.1155/2018/1435302.
2. Lepareur N, Ardisson V, Noiret N, Garin E. ^188^Re-SSS/Lipiodol: development of a potential treatment for HCC from bench to bedside. Int J Mol Imaging. 2012;Article ID 278306:9. https://doi.org/10.1155/2012/278306.
3. Delaunay K, Edeline J, Rolland Y, Lepareur N, Laffont S, Palard X, Bouvry C, Le Sourd S, Pracht M, Ardisson V, Noiret N, Bellissant É, Garin E. Preliminary results of the Phase 1 Lip-Re I clinical trial: biodistribution and dosimetry assessments in hepatocellular carcinoma patients treated with ^188^Re-SSS Lipiodol radioembolization. Eur J Nucl Med Mol Imaging. 2019;46:1506–17.

## OP28

### Theranostic potential of [67Cu]Cu-NOTA-trastuzumab against HER2 positive breast cancer

#### Jessica Pougoue Ketchemen

##### Department of Medical Imaging, College of Medicine, University of Saskatchewan, Saskatoon, SK, S7N 0W8, Canada

*EJNMMI Radiopharmacy and Chemistry* 2023; 8(Suppl1): OP28

**Aim:** Breast cancer (BC) is the most common cancer in women, and its effective management is still a global challenge despite recent advances^1^. Human epidermal growth factor receptor 2 (HER2) is overexpressed in 25–30% of BC, and is characterized by poor diagnosis, recurrence, and aggressiveness^2^. The effectiveness of approved anti-HER2 therapies is modest, especially in advanced disease and resistance to them is common^3^. Radioimmunotherapy has been effective in other cancers. Copper-67 (67Cu) is a promising theranostic isotope because its 185 keV gamma rays are excellent for immunoSPECT, and it has ideal beta emissions (mean 141 keV and maximum 562 keV) for therapy^4^. Here, we have developed a 67Cu-labeled anti-HER2 monoclonal antibody trastuzumab as a theranostic against HER2 positive BC. First, we evaluated which chelator (p-SCN-Bn-NETA, p-SCN-Bn-DOTA or p-SCN-Bn-NOTA showed the best properties in terms of 67Cu-labeling and stability of the formed complex. The most promising chelator was used afterwards to develop the 67Cu-labeled trastuzumab and the construct was evaluated using HER2 positive cells with different receptor densities, and pharmacokinetics were evaluated in healthy mice.

The aim of this study was to develop a 67Cu-labeled trastuzumab using a suitable bifunctional chelator and evaluate its theranostic properties

**Materials and methods:** We conjugated trastuzumab with p-SCN-Bn-NOTA, p-SCN-Bn-NETA, or p-SCN-Bn-DOTA, and radiolabeled it with [67Cu]CuCl2. HPLC, iTLC and bioanalyzer were used to determine the radiochemical yield and purity. Stability of the complexes was studied in human serum and PBS over a period of 5 days at 37 °C. Binding characteristics were determined using flow cytometry in BT-474 (high HER2 density) and MCF-7 (low HER2 density). In vivo stability was studied in healthy nude mice over a 3-day period. Internalization of the immunoconjugate was evaluated using Incucyte live-cell imaging. ImmunoSPECT imaging and biodistribution of [67Cu]Cu-NOTA-trastuzumab were done in mice bearing BT-474 and MCF-7 xenografts at 24 h, 48 h and 120 h post injection.

**Results:** Pure and homogenous NETA-trastuzumab, DOTA-trastuzumab and NOTA-trastuzumab conjugates (> 95%) were obtained as shown by SEC-HPLC and bioanalyzer. All compounds were obtained in high RCY (≥ 90%) and RCP (≥ 90%), as confirmed with iTLC and radio-HPLC, with an apparent molar activity of 1 MBq/µg. After 5 days, in vitro stabilities were 31 ± 10.7%, 28 ± 7% and 97 ± 3% in human serum, and 94 ± 2%, 86 ± 4%, and 79 ± 6% in PBS for [67Cu]Cu-NETA-trastuzumab, [67Cu]Cu-DOTA-trastuzumab, and [67Cu]Cu-NOTA-trastuzumab, respectively. Hence, we proceeded with [67Cu]Cu-NOTA-trastuzumab. Flow cytometry showed low KD and EC50 values of trastuzumab (10.8 nM and 10.3 nM) and NOTA-trastuzumab (29.5 nM and 15.9 nM), with NOTA-rituximab (control IgG) showing no binding. Internalization was HER2 density dependent. [67Cu]Cu-NOTA-trastuzumab was stable in vivo as with low bone and kidney uptake was observed. Xenografts were well delineated using microSPECT/CT, and tumor uptake was 32.3 ± 1.8% IA/g (BT-474) and 15.3 ± 3.0% IA/g (MCF-7) at 24 h p.i, and 33.1 ± 10.6% IA/g (BT-474) and 14.7 ± 2.8% IA/g (MCF-7) at 120 h p.i.

**Conclusion:** NOTA was the most promising 67Cu-chelator tested in this study in terms of radiolabelling efficiency and stability. [67Cu]Cu-NOTA-trastuzumab is a potential promising theranostic agent against HER2 positive BC with good pharmacokinetic properties. Further pharmacokinetics, dosimetry, and therapy studies are ongoing.

**References**

1. Wilkinson L, Gathani T. Understanding breast cancer as a global health concern. Br J Radiol. 2022;95(1130):20211033.
2. Latta EK, Tjan S, Parkes RK, O’Malley FP. The role of HER2/neu overexpression/amplification in the progression of ductal carcinoma in situ to invasive carcinoma of the breast. Mod Pathol. 2002;15(12):1318–25.
3. Gote V, Nookala AR, Bolla PK, Pal D. Drug resistance in metastatic breast cancer: tumor targeted nanomedicine to the rescue. Int J Mol Sci. 2021;22(9):4673.
4. Merrick MJ, Rotsch DA, Tiwari A, et al. Imaging and dosimetric characteristics of 67 Cu. Phys Med Biol. 2021;66(3):035002.

## OP29

### Site-specific Zr-89 labeling of a VHH construct as a half-life extending building block for PET applications.

#### Leekens S.^1^, Cawthorne C.^2^, Bormans G.^1^, Casteels P.^3^, Cleeren F.^1^

##### ^1^Radiopharmaceutical Research, Department of Pharmacy and Pharmacology, University of Leuven, Leuven. ^2^ Nuclear Medicine and Molecular Imaging, Department of Imaging and Pathology, University of Leuven, Leuven, Belgium. ^3^Sanofi Ghent, Ghent, Belgium

*EJNMMI Radiopharmacy and Chemistry* 2023; 8(Suppl1): OP29

**Aim:** Single-domain antibodies or VHH constructs display excellent features for PET imaging applications like high specificity, affinity and stability, decreased production cost, low immunogenicity, and improved tissue penetration. Their convenient, low molecular weight allows fast renal drainage and therefore afford rapid target to background ratios, which is ideal for immunoPET using short-lived radionuclides (e.g., Ga-68 or F-18) [1]. On the contrary, rapid clearance of the tracer may impact target accumulation, a pivotal parameter in defining its therapeutic potential (TRNT) [2]. Therefore, a generic technique for half-life extension (HLE) using an albumin-binding VHH building block (ALB) engineered with a C-terminal, free cysteine can be an interesting tool to prolong the biological half-life of VHH or protein constructs in general. For vector molecules with a longer physical half-life, zirconium-89 (Zr-89, T1/2: 3.3 days) is often considered an ideal PET radionuclide, with DFO* as the preferred chelator [3]. Here we describe an optimized protocol for site-specific Zr-89 labeling of proteins employing thiol-maleimide chemistry in combination with DFO*-maleimide.

**Materials and methods:** The ALB VHH was reduced in 10 mM dithiothreitol (DTT) in phosphate-buffered saline (PBS, pH 7.4), whereafter it was buffer exchanged to 0.10 mM tris(2-carboxyethyl)phosphine (TCEP) in PBS (pH 7.4). A 2.5 excess of DFO*-maleimide was added while incubating the sample at 37 °C for 60 min. The crude reaction was purified after overnight incubation at 4 °C using preparative size exclusion chromatography (SEC), and conjugate quality was confirmed by SEC and UPLC. DFO*-ALB was radiolabeled with Zr-89 (HEPES, 0.5 M, pH 7.2, 37 °C, 60 min) and purified by SEC. In vitro stability of the tracer was assessed in serum at 37 °C using radioSEC and iTLC. In vivo evaluation was performed in healthy NMRI mice (n = 4) using µPET/CT at 4, 24, and 48 h p.i., the latter followed by a biodistribution study. Mice were administered a bolus injection of ± 3 MBq [^89^Zr]Zr-DFO*-ALB, 5 mg/kg tracer, adjusted with non-labeled ALB via i.v. tail injection.

**Results:** DTT is incompatible during thiol-maleimide reactions, so a buffer exchange to a TCEP buffer was necessary. Excess free thiol-containing ALB VHH was allowed to oxidize overnight to form dimers and was separated, together with the excess chelator, by SEC purification. The final conjugate DFO*-ALB revealed a monodisperse profile on SEC and showed an average neutral mass that conforms with the predicted theoretical mass. DFO*-ALB was obtained with a recovery of ± 90% with high purity and without the formation of aggregates. Radiolabeling of DFO*-ALB with Zr-89 was successful (95%; determined by iTLC) and excellent RCP (99%) after SEC. [^89^Zr]Zr-DFO*-ALB showed good stability (90 ± 0.7%) in serum after 48 h. As expected, µPET/CT evaluation showed blood pool accumulation at all time points, with the heart and major vessels clearly visible. Biodistribution data at 48 h support the PET data by revealing high tracer concentrations in the blood pool (SUV: 2.31 ± 0.22, 16.2 ± 1.6% ID) and also high uptake in the lungs (SUV: 1.43 ± 0.26), heart (SUV: 0.91 ± 0.04) and kidney (SUV: 0.82 ± 0.06) was observed which corresponds to the expected pharmacokinetics of an albumin-binding VHH building block.

**Conclusion:** Site-specific Zr-89 labeling of DFO*-ALB proved to be an efficient process with excellent RCC. µPET/CT and biodistribution data corresponded with the expected pharmacokinetics of an albumin binding protein. In general, we highlight the potential of an albumin-binding building block for half-life extension in the context of TRNT and imaging using site-specific Zr-89-DFO*-labeling.

**References**

1. Jovčevska I, Muyldermans, . The Therapeutic potential of nanobodies. BioDrugs. 2020;34:11–26.
2. Kijanka M, Dorresteijn B, Oliveira S, van Bergen en Henegouwen PM. Nanobody-based cancer therapy of solid tumors. Nanomedicine. 2015;10:161–74.
3. Vugts DJ, et al. Comparison of the octadentate bifunctional chelator DFO*-pPhe-NCS and the clinically used hexadentate bifunctional chelator DFO-pPhe-NCS for ^89^Zr-immuno-PET. Eur J Nucl Med Mol Imaging. 2017;44:286–95.

## OP30

### How to radiolabel a hydrophobic VHH with Tc-99m for Blood Brain Barrier crossing evaluation?

#### S. Bacor, M. Ahmadi, C. Lombardi, N. De Leiris, M. Debiossat, D. Fagret, P. Perret, C. Ghezzi

##### Université Grenoble Alpes, Laboratoire Radiopharmaceutiques Biocliniques (LRB), Inserm, CHU Grenoble Alpes, 38000 Grenoble, France

*EJNMMI Radiopharmacy and Chemistry* 2023; 8(Suppl1): OP30

**Aim:** In order to develop a new radiotracer for early diagnosis of Alzheimer's disease, a nanobody (VHHref) which consists of the unique variable domain of naturally occurring heavy-chain antibodies of camelidae (a), and targeting a specific form of the Tau protein in vitro and ex vivo has been modified and optimized to penetrate the brain in vivo. Two cell-penetrating peptides (CPP), CPP1 and CPP2, have been combined with VHHref to improve its ability to cross the Blood Brain barrier (BBB) and increase its cerebral availability. An evaluation of crossing a differentiated cellular BBB model has been then performed for these three VHHs. VHHref-CPP2, which is a promising candidate present a hydrophobic character more important than VHHref. However, it has been also a challenge in terms of radiolabeling, being likely to aggregate more easily depending on the conditions. The VHHs contain a poly-Histidine Tag (poly-His) used as a specific site suitable for labeling with Tc-99m using tricarbonyl method. The good accessibility of the poly-His Tag is crucial to radiolabel with a good labeling yield. The aim of this study was to show the need to add excipients to label with Tc-99m an hydrophobic VHH in order to maintain a good solubility during the radiolabeling process and also purification step.

**Materials and methods:** Circular dichroism (CD) spectra were obtained from 200 to 260 nm at temperatures ranging from 25 to 80 °C to determine the best temperature of incubation for radiolabeling of VHHref, VHHref-CPP1 and VHHref-CPP2.

In a first time, VHHref, VHHref-CPP1, VHHref-CPP2 were radiolabeled with Tc-99 m without excipient. In a second time, we studied several excipients (Tween, a mixture of glycerol and Tween) at different percentage during radiolabeling of the VHHref-CPP2 and another hydrophobic VHHt-CPP2.

After all radiolabeling, a purification step was necessary with a NAP-5 size exclusion column. Different excipients (BSA, Tween, a mixture of glycerol and Tween) were also tested to avoid aggregation and to improve the elution performance on the column. The radiochemical purity (RCP) was determined by radio-HPLC before and after purification step with the NAP column. The partition coefficients (log *P*) of the labeled compounds were determined by measuring the distribution for each radiolabeled complex between 1‐octanol and phosphate‐buffered saline (PBS) so as to assess their lipophilicity.

**Results:** The conformational changes observed by CD indicated a fairly average thermal resistance of the VHHs below 50 °C. The incubation temperature chosen for radiolabeling was 50 °C for VHHref and VHHref-CCP1 while for VHHref-CCP2 it was reduced at 37 °C.

VHHref and VHHref-CPP1 were successfully radiolabeled without excipient in the labeling medium which was not the case for VHHref-CPP2. In fact, more than 80% of radioactivity remained on the purification columns due to probable formation of aggregates during radiolabeling.

Tween did not interfere in the labeling and increased the labeling yield, with a higher RCP before purification. The addition of a mixture of glycerol and of Tween during labeling shown even better RCP before purification (close to 70%). These results revealed that the presence of excipient during hydrophobic VHH radiolabeling was essential to keep the solubility of these VHHs and to avoid the possible formation of hydrophobic interactions (aggregates formation). Furthermore, no significant difference was observed on the RCP after purification, regardless of the excipient used at this step. On the other hand, it was necessary for this step to use an excipient in order to avoid non-specific interactions and the formation of aggregates on the column.

**Conclusion:** In this study, we showed that adding excipient was necessary to radiolabel with Tc-99m hydrophobic VHHs, like VHHref-CPP2. Among all tested excipients, the mixture glycerol and Tween was the most favorable for maintaining good solubility of the compound without interference with the radiolabeling, therefore allowing obtaining successfully radiolabeling with a good labeling yield. These steps could be applied as a standard procedure to radiolabel other hydrophobic compounds that have been produced without excipient. However, the optimal strategy will most probably be to produce the molecule with the appropriate excipient from the production step and keep it throughout the labeling protocol as we have also demonstrated for VHHref-CPP2 and VHHt-CPP2.

**Reference**

1. Muyldermans S, et al. Nanobodies: natural single-domain antibodies. Annu Rev Biochem. 2013;82:775–97.

## OP31

### Metabolites identification by ESI(+)-MS spectroscopy of the selective αvβ3 RGDechi peptide and of two [^99m^Tc][Tc(N)PNP]-tagged derivatives

#### C. Bolzati^1^, N. Salvarese^1^, R. Seraglia^1^, C. Gobbi^1^, D. Carpanese^2^, L. Meléndez-Alafort^2^, A. Rosato^2,3^, M. Saviano^4^, A. Del Gatto^5^, L. Zaccaro^5^

##### ^1^Institute of Condensed Matter Chemistry and Technologies for Energy ICMATE-CNR, Corso Stati Uniti, 4, 35127 Padova, Italy. ^2^Veneto Institute of Oncology IOV-IRCCS, Via Gattamelata, 64, 35128 Padova, Italy. ^3^Department of Surgery, Oncology and Gastroenterology, University of Padova, Via Gattamelata, 64, 35128 Padova, Italy. ^4^Institute of Cristallography IC−CNR, Via Amendola 122/O, 70126 Bari, Italy. ^5^Institute of Biostructures and Bioimaging IBB-CNR, Via Pietro Castellino 111, 80134 Napoli, Italy

*EJNMMI Radiopharmacy and Chemistry* 2023; 8(Suppl1): OP31

**Aim:** RGDechi peptide is a potent and selective antagonist of αvβ3 receptors [1, 2]. This nineteen amino-acids peptide comprises a cyclic portion containing the RGD motif, recognized by all integrins, and a linear sequence derived from the C-terminal portion of echistatin, responsible for β3-subunit specificity. Animal studies of [^99m^Tc][Tc(N)PNP]-tagged CysRGDechi (^99m^Tc1), CysRGDechi1-17 (^99m^Tc2) derivatives confirmed the ability of the peptides to discriminate between αvβ3 and αvβ5 integrins in vivo, showing promising biodistributions, but low absolute uptake in αvβ3 positive tumors [3]. This might be a consequence of their in vivo degradation, as proved by the presence of metabolites in urine.

With the aim of improving the biological stability of these compounds and enhancing their bioavailability and accumulation at the tumor site, the biodegradation products of RGDechi and ^99m^Tc1-2 were identified.

**Materials and methods:** ^99m/99g^Tc1-2 were synthesized under carrier-added conditions and characterized by RP-HPLC (UV/Radio) combined with LC-MS analyzes.

To define the hydrolysis/cleavage products, RGDechi and ^99m/99g^Tc1-2 were incubated, at 37 °C, in mouse and human sera and in fresh mouse kidney homogenate. The mixtures were analyzed by RP-HPLC-UV/Radio, LC-MS and MALDI-TOF-MS [4].

**Results:** The chemical identity of ^99m/99g^Tc1-2 was established to be in agreement with the proposed formulation consisting of the [Tc(N)PNP]-scaffold bound to one cysteine-N,S chelator carrying the peptide, in a syn/anti isomeric arrangement.

Stability studies on RGDechi allowed identification of the main enzymatic cleavage site located between the Pro17-Ala18 residues of the peptide [4].

A different metabolic pathway was detected for ^99m/99g^Tc1-2. Compounds were stable in sera, but not in mouse kidney homogenate, since the collected chromatograms showed a significant shift of the peaks. Similar outcomes were detected in HPLC analysis of urine collected from animals injected with ^99m^Tc1-2. Chromatograms reveled a good coincidence between stability in vivo and in tissue-homogenates.

For both radiolabeled peptides, a common cleavage site, invariably from the syn or anti nature of the compounds, has been identified between the Asp7-Asp8 at the echistatin portion.

**Conclusion:** These findings indicate a different metabolic pathway of the radiolabeled peptides compared to the unlabeled RGDechi, probably because of the conjugation of the [Tc(N)PNP]-moiety to the amino-acid sequence, which could limit the recognition of the peptides by serum enzymes.

Data provide important information about the approach that can be adopted to improve the enzymatic stability of the peptide, which can result in the overall magnification of the pharmacokinetic profile of the corresponding radiolabeled compounds.

**Acknowledgment**

The authors acknowledge Associazione Italiana per la Ricerca sul Cancro (AIRC) for financial support (AIRC, IG 2020 ID 24528).

**References**

1. Del Gatto A, Zaccaro L, Grieco P, Novellino E, Zannetti A, Del Vecchio S, Iommelli F, Salvatore M, Pedone C, Saviano M. Novel and selective αvβ3 receptor peptide antagonist: design, synthesis, and biological behavior. J Med Chem. 2006;49(11):3416–20. https://doi.org/10.1021/jm060233m.
2. Zannetti A, Vecchio SD, Iommelli F, Gatto AD, Luca SD, Zaccaro L, Papaccioli A, Sommella J, Panico M, Speranza A, et al. Imaging of αvβ3 expression by a bifunctional chimeric RGD peptide not cross-reacting with Αvβ5. Clin Cancer Res. 2009;15(16):5224–33. https://doi.org/10.1158/1078-0432.CCR-08-3270.
3. Bolzati C, Salvarese N, Carpanese D, Seraglia R, Meléndez-Alafort L, Rosato A, Capasso D, SavianoM, Del Gatto A, Comegna D, et al. [^99m^Tc][Tc(N)PNP43]-labeled RGD peptides as new probes for a selective detection of αvβ3 integrin: synthesis, structure–activity and pharmacokinetic studies. J Med Chem. 2018;61(21):9596–610. https://doi.org/10.1021/acs.jmedchem.8b01075.
4. Comegna D, Zannetti A, Del Gatto A, de Paola I, Russo L, Di Gaetano S, Liguoro A, Capasso D, Saviano M, Zaccaro L. Chemical modification for proteolytic stabilization of the selective αvβ3 integrin RGDechi peptide: in vitro and in vivo activities on malignant melanoma cells. J Med Chem. 2017;60(23):9874–84. https://doi.org/10.1021/acs.jmedchem.7b01590.

## OP32

### Site-specific tagging of scFvD2B-HysTag with [^99m^Tc][Tc(CO)3]+ framework for SPECT imaging of PSMA in prostate cancer: preliminary in vitro investigation

#### C. Gobbi^1^, B. Spolaore^2^, D. Carpanese^3^, V. Rossi^3^, N. Salvarese^1^, L. Melendez-Alafort^3^, Antonio Rosato^3,4^, G. Fracasso^4^, C. Bolzati^1^

##### ^1^ICMATE-CNR, Padova, Italy. ^2^Department of Pharmaceutical Science, University of Padua, Italy. ^3^Istituto Oncologico Veneto IOV-IRCCS, Padua, Italy. ^4^Department of Medicine, University of Verona, Verona, Italy

*EJNMMI Radiopharmacy and Chemistry* 2023; 8(Suppl1): OP32

**Aim:** scFvD2B is a single chain variable fragment (29 kDa) of the monoclonal antibody IgGD2B specific to the extracellular domain of prostate specific membrane antigen (PSMA) overexpressed in prostate cancer (PCa).^1,2^ scFvD2B has shown promising properties, in terms of high stability, favorable pharmacokinetics and specificity, efficiently accumulating in PSMA-expressing PCa tumors. The small size of scFvD2B allows both faster penetration into tumor sites and rapid clearance from non-target organs, allowing the attainment of good contrast and sensitivity on the day of injection. This consents the use of relatively short-lived radionuclides such as ^99m^Tc or 64Cu with the benefit of an appreciable reduction in the dose absorbed by patients compared to other radionuclides.

Hexahistidine tags (His-tags), incorporated into recombinant proteins to facilitate purification using metal-affinity chromatography, are useful binding sites for radiolabeling with [^99m^Tc(CO)3]+framework.

Therefore, in order to obtain a radioimmunoconjugate suitable for PCa SPECT imaging, His-Tag sequence was engineered at the C-terminal portion of scFvD2B and labeled using the [^99m^Tc(CO)3]+system. 3

For comparison the labeling of the corresponding no Tag scFvD2B was also assessed.

**Materials and methods:** scFvD2B-HisTag and no Tag scFvD2B were synthesized by cloning the IgGD2B VH and VL chain genes into an E. Coli vector. [^99m^Tc][Tc(CO)_3_(OH_2_)_3_]+ was produced through the commercial IsoLink® kit and the radiolabeling was performed at 37 °C for 2 h using 100–150 µg of scFvD2B-HysTag or no Tag scFvD2B in a final volume of 250 µL. Radioimmunoconjugates were characterized by RP-HPLC. [^99m^Tc][Tc(CO)_3_]-scFvD2B-HisTag was purified using NAP5 size exclusion column, and evaluated for stability in PBS, human serum, and transchelation (His, Cys, GSH, EDTA 10 mM). Cellular uptake and internalization were assessed in PSMA(+) (LNCaP and PC3-PIP) and PSMA(−) (PC3) cell lines.

**Results:** Under the above-mentioned labeling conditions, [^99m^Tc][Tc(CO)_3_]-scFvD2B-HisTag returned a RCY in the range 28–43%; after purification RCP was 98%. Meanwhile, no formation of [^99m^Tc][Tc(CO)3]-labeled no tag scFvD2B was detected. [^99m^Tc][Tc(CO)_3_]-scFvD2B-HisTag showed a steady stability profile over time. According to cell studies, the uptake and internalization values were encouraging for future in vivo biodistribution insights. The values were: in LNCaP cells 6% and 4% respectively, in PC3-PIP 30% and 15%; in PC3 3% and 0.7%. Blocking studies, with an excess of scFvD2B-HisTag, confirmed the specificity of [^99m^Tc][Tc(CO)_3_]-scFvD2B-HisTag for PSMA receptor.

**Conclusion:** [^99m^Tc][Tc(CO)_3_]-scFvD2B-HisTag was easily produced with a high RCP. The reaction was site-specific and the radioimmunoconjugate was stable in vitro and possessed a high accumulation in PSMA(+) cells. The process was receptor mediated. Studies are in progress to determine its in vivo performance.

**Funding**

The authors acknowledge Associazione Italiana per la Ricerca sul Cancro (AIRC) for financial support (AIRC, IG 2020 ID 24528).

**References**

1. Carpanese D, et al. Targeting prostate cancer with the anti-PSMA scFvD2B: a theranostic promise for nuclear medicine. Clin Transl Imaging. 2019;7(4):295–301.
2. Carpanese D, et al. Development of ^177^Lu-scFvD2B as a potential immunotheranostic agent for tumors overexpressing the prostate specific membrane antigen. Sci Rep. 2020;10.
3. Williams JD, et al. Optimal his-tag design for efficient [^99m^Tc(CO)_3_]+ and [^188^Re(CO)_3_]+ labeling of proteins for molecular imaging and radionuclide therapy by analysis of peptide arrays. Bioconjug Chem. 2020;32:1242–54.

## OP33

### ^68^Ga-Ornibactin for Burkholderia cepacia complex infection imaging using positron emission tomography

#### Bendova K.^1^, Raclavsky V.^3^, Novotny R.^3^, Novy Z.^1,2^, Hajduch M.^1,2^, Petrik M.^1,2^

##### ^1^Institute of Molecular and Translational Medicine, Faculty of Medicine and Dentistry, Palacky University Olomouc. ^2^Czech Advanced Technology and Research Institute, Palacky University Olomouc. ^3^Department of Microbiology, Faculty of Medicine and Dentistry, Palacky University Olomouc

*EJNMMI Radiopharmacy and Chemistry* 2023; 8(Suppl1): OP33

**Aim:** Although bacteria from Burkholderia cepacia complex (BCC) are generally considered non-pathogenic to healthy population, some of these species may cause severe nosocomial infections in immunocompromised patients, especially those with granulomatous disease and cystic fibrosis (1). Due to the BCC’s unpredictable behaviour in these patients and its resistance to various antibiotics and disinfectants, accurate diagnostic tool is required, so that an adequate and effective treatment could be initiated(2). We present here the use of gallium-68 labelled siderophore ornibactin, low molecular weight chelator produced by BCC for iron scavenging, for BCC infection imaging by the means of positron emission tomography (PET).

**Materials and methods:** Ornibactin was labelled with gallium-68 and radiochemical purity of resulting complex was measured on RP-HPLC. In vitro studies, including stability in various solutions, plasma protein binding values and partition coefficient, of ^68^Ga-Ornibactin were performed. The uptake was evaluated in several members of BCC as well as in diverse respiratory pathogens. Ex vivo biodistribution studies were performed in non-infected mice and in mice with hind muscle infection. In vivo imaging with PET/CT was done in a mouse model of muscle infection and in a rat model of lung infection.

**Results:** Ornibactin can be labelled with ^68^Ga with high radiochemical purity (> 95%), this complex shows great stability in human serum (97.99 ± 1.35% after 2 h incubation), it has hydrophilic properties (log *P* = − 2.67 ± 0.05%) and low plasma protein binding values (6.72 ± 0.71% after 2 h incubation). Its in vitro uptake reaches highest values in Burkholderia multivorans culture (7873.55 ± 84.11 %AD/g culture), while in other respiratory pathogens it is significantly lower (highest measured value in S. aureus, 982.09 ± 49.89 %AD/g culture). Ex vivo and in vivo results show rapid pharmacokinetics with renal excretion and accumulation of the complex in the site of infection in both animal models.

**Conclusion:** It is feasible to radiolabel Ornibactin with gallium-68 with high radiochemical purity. ^68^Ga-Ornibactin has promising in vitro characteristics and high specificity for BCC. It shows optimal pharmacokinetics and accumulates well in the site of infection, making it promising compound for BCC infection imaging.

**Funding**

The authors received financial support from the European Regional Development Fund—Project ENOCH (No. CZ.02.1.01/0.0/0.0/16_019/0000868), the European infrastructure for translational medicine EATRIS-ERIC-CZ (No. LM2018133), the Internal Grant Agency of Palacky University (Project number: IGA_LF_2022_012) and the project National Institute of virology and bacteriology (Programme EXCELES, ID Project No. LX22NPO5103)—Funded by the European Union - Next Generation EU.

**References**

1. Häfliger E, Atkinson A, Marschall J. Systematic review of healthcare-associated burkholderia cepacia complex outbreaks: presentation, causes and outbreak control. Infect Prev Pract. 2020;2(3). https://doi.org/10.1016/j.infpip.2020.100082.
2. Sfeir MM. Burkholderia cepacia complex infections: more complex than the bacterium name suggest. J Infect. 2018;77(3):166–70. https://doi.org/10.1016/j.jinf.2018.07.006.

## OP34

### ^68^Ga-siderophores for imaging ***Escherichia coli*** infection: selection of suitable candidates

#### M. Petrik^1^, K. Bendova^1^, A. Palyzova^2^, Z. Novy^1^, V. Havlicek^2,3^, M. Popper^1^, M. Hajduch^1^

##### ^1^Institute of Molecular and Translational Medicine, Faculty of Medicine and Dentistry and Czech Advanced Technology and Research Institute, Palacky University, Olomouc, Czech Republic. ^2^Institute of Microbiology of the Czech Academy of Sciences, v.v.i., Prague, Czech Republic. ^3^Department of Analytical Chemistry, Faculty of Science, Palacky University, Olomouc, Czech Republic

*EJNMMI Radiopharmacy and Chemistry* 2023; 8(Suppl1): OP34

**Aim:** Increased mortality rates from infectious diseases is a growing public health concern (1). Timely and accurate diagnosis of infection is crucial for effective patient care. New and better approaches are needed for early diagnosis and monitoring of infections. Molecular imaging allows for longitudinal, non-invasive assessments and can provide key information about infectious processes deep within the body (2). Siderophores are iron-specific chelators recognized by specific microbial transporters, representing one of few fundamental differences between microbial and mammalian cells. Replacing iron by gallium-68 without loss of bioactivity is possible allowing molecular imaging by positron emission tomography (PET) (3). Here, we report on the evaluation of siderophores produced and/or utilized by *Escherichia coli*, radiolabelled with Ga-68, for the detection of *E. coli* infections.

**Materials and methods:** Selected siderophores synthesized or utilized by *E. coli* were radiolabelled with Ga-68 using acetate buffer. Radiochemical purity was analyzed by ITLC-SG and/or RP-HPLC. Partition coefficient, stability in different media and plasma protein binding values were determined to characterize the studied ^68^Ga-siderophores in vitro. In vitro uptake of the selected ^68^Ga-siderophores was tested in various microbial cultures. Ex vivo biodistribution was studied in normal Balb/c mice at 30 and 90 min p.i.. Introduction of different animal models of *E. coli* infection and in vivo evaluation of the studied ^68^Ga-siderophores by PET/CT or PET/MRI was initiated.

**Results:** Tested siderophores were labelled with ^68^Ga with high (> 95%) radiochemical purity. The resulting complexes exhibited hydrophilic properties (log *P* ranging from − 4.04 to − 2.72), high stability (~ 85–99% by 120 min incubation) in human serum and variable plasma protein binding (~ 5–60% by 120 min incubation). In vitro uptake of ^68^Ga-siderophores was highly dependent on the type of microbial culture. Most of the tested ^68^Ga-siderophores showed high uptake in *E. coli* cultures. In normal mice, some ^68^Ga-siderophore showed relatively rapid renal excretion and low blood values, whereas other potential candidates for *E. coli* detection displayed high kidney retention, excretion through the gastrointestinal tract and significant retention in the blood 90 min after injection. Initial PET/CT images of infected animals showed specific accumulation of selected ^68^Ga-siderophores in *E. coli* infected tissues.

**Conclusion:** Different siderophores utilized by *E. coli* can be labelled with Ga-68 with high affinity and radiochemical purity. Most of the studied ^68^Ga-siderophores displayed suitable in vitro characteristics and acceptable pharmacokinetics properties in mice. The high and specific uptake of most of the studied ^68^Ga-siderophores by *E. coli* was confirmed in vitro, proving their potential for imaging of *E. coli* infections in vivo. Imaging of *E. coli* infection in animal models using ^68^Ga-siderophores is currently ongoing.

**Funding**

We gratefully acknowledge the financial support of the project National institute of virology and bacteriology (Programme EXCELES, ID Project No. LX22NPO5103)—Funded by the European Union—Next Generation EU and the European Regional Development Fund (Project ENOCH No. CZ.02.1.01/0.0/0.0/16_019/0000868).

**References**

1. Polvoy I, et al. Nuclear imaging of bacterial infection: the state of the art and future directions. J Nucl Med. 2020;61:1708–16.
2. Ordonez AA, et al. Molecular imaging of bacterial infections: overcoming the barriers to clinical translation. Sci Transl Med. 2019;11:eaax8251.
3. Petrik M, et al. ^68^Ga-labelled desferrioxamine-B for bacterial infection imaging. Eur J Nucl Med Mol Imaging. 2021;48:372–82.

## OP35

### Developing a radiolabelled tracer for prospective PET imaging of *Plasmodium falciparum* infection

#### Janie Duvenhage^1,2,3^, Jan Rijn Zeevaart^2,3,4^, Cecile Swanepoel^3^, Liani Smith^3^, Lyn-Marie Birkholtz^1^, Thomas Ebenhan^2,3,5^

##### ^1^Department of Biochemistry, Genetics and Microbiology, Institute of Sustainable Malaria Control, University of Pretoria, Pretoria, South Africa. ^2^Radiochemistry, the South African Nuclear Energy Corporation (Necsa) SOC Ltd, Pelindaba, South Africa. ^3^Nuclear Medicine Research Infrastructure NPC, Pretoria, South Africa. ^4^DSI/Preclinical Drug Development Platform, North-West University, Potchefstroom, South Africa. ^5^Department of Nuclear Medicine, University of Pretoria, Pretoria, South Africa

*EJNMMI Radiopharmacy and Chemistry* 2023; 8(Suppl1): OP35

**Aim:** *Plasmodium falciparum* (*P. falciparum*) causes the most severe cases of malaria. Severe malaria (SM), an understudied multisystem disease, affects the host’s organs and can lead to serious complications, some of which leaves life-long neurological and cognitive sequela. Nuclear imaging could aid in investigating the host-parasite mechanisms that governs malaria pathogenicity, however, there are no malaria-specific tracers. Therefore, the aim was to develop zirconium-89 (^89^Zr) radiolabeled malaria-specific tracers, namely, a Plasmodium-specific antibody (IIIB6) and fragment antibody (Pf-Fab), to assess their in vivo biodistribution by micro-PET/CT imaging.

**Materials and methods:** Ethical clearance was obtained through the North-West University’s Animal Research Ethics Committee (AnimCare REC: NWU-00175-18-S5). IIIB6 and Pf-Fab was conjugated to Bz-DFO-NCS followed by ^89^Zr-complexation in ammonium acetate (0.1 M) for 1 h at 25 °C (pH 7). The stability and biodistribution of [^89^Zr]Zr-IIIB6 and [^89^Zr]Zr-Pf-Fab were assessed in BALB/c mice by VOI-analysis of micro-PET/CT images over 24 h. These tracers’ post-mortem biodistribution were compared to [^89^Zr]Zr-h-R3, a monoclonal antibody approved for human tumour imaging, and [^89^Zr]Zr-oxalate (control).

**Results:** Evaluation of cardiac region in micro-PET/CT images for mice over 24 h indicated pharmacological half-lives of 7.1–12.1 h and 3.5–6.0 h for [^89^Zr]Zr-IIIB6 and [^89^Zr[Zr-Pf-Fab, respectively. SUV-based time-activity curves identified unfavorably high hepatic tracer concentrations of [^89^Zr]Zr-IIIB6 at 2–6 h followed by spleen > kidneys > heart > stomach > lung and femur. In the case of [^89^Zr]Zr-Pf-Fab, high kidney uptake was found at 4–6 h post injection, followed by liver > lung > stomach and femur. Ex vivo biodistribution studies showed high stability of both tracers as indicated by the low bone uptake compared to [^89^Zr]Zr-oxalate. These tracers’ similar biodistribution to that of [^89^Zr]Zr-h-R3 confirmed their lack of organ toxicity and lack of “target organ”.

**Conclusion:** Micro-PET/CT-imaging demonstrated that [^89^Zr[Zr-Pf-Fab has a preferred pharmacological profile over [^89^Zr]Zr-IIIB6 towards future PET/CT-imaging of *P. falciparum*-infection. Radiation levels in kidneys may be limiting, yet further characterisation of [^89^Zr]Zr-Pf-Fab specificity and sensitivity is encouraged in malaria-infected animals.

**References**

1. Derbie A, et al. Therapeutic efficacy of artemether-lumefantrine (Coartem®) for the treatment of uncomplicated falciparum malaria in Africa: a systematic review. J Parasitol Res. 2020.
2. Siddiqui AJ, et al. Neurological disorder and psychosocial aspects of cerebral malaria: What is new on its pathogenesis and complications? A minireview. Folia Parasitol. 2020;67:015.
3. Nishanth G, Schlüter D. Blood–brain barrier in cerebral malaria: pathogenesis and therapeutic intervention. Trends Parasitol. 2019;35(7):516–28.
4. Holland JP, et al. ^89^Zr-DFO-J591 for immunoPET of prostate-specific membrane antigen expression in vivo. J Nucl Med. 2010;51(8):1293–300.

## PP01

### Raduiisotope nanocarrier systems for cancer theranostics based on radiation-synthesized polymer nanogels

#### Beata P. Rurarz^1,2^, Joanna Raczkowska^1^, Natalia Gibka^1^, Małgorzata Bukowczyk^1^, Karolina Kowalska^2^, Kinga A. Urbanek^2^, Agnieszka W. Piastowska-Ciesielska^2^,Michał Maurin^3^, Agnieszka Sawicka^3^, Sławomir Kadłubowski^1^, Piotr Ulański^1^, Urszula Karczmarczyk^3^

##### ^1^Institute of Applied Radiation Chemistry, Faculty of Chemistry, Lodz University of Technology, Wróblewskiego 15, 93-590 Łódź, Poland. ^2^Medical University of Lodz, Department of Cell Cultures and Genomic Analysis, Żeligowskiego 7/9, 90‐752 Łódź, Poland. ^3^National Centre for Nuclear Research, Radioisotope Centre POLATOM, Andrzeja Sołtana 7, 05-400 Otwock, Poland; urszula.karczmarczyk@polatom.pl

*EJNMMI Radiopharmacy and Chemistry* 2023; 8(Suppl1): PP01

**Aim:** The application of nanomaterials in medicine may open new diagnostic and therapeutic perspectives. Therefore, we have evaluated a novel nanoplatform based on poly(acrylic acid) (PAA) nanogels, functionalised with DOTA-bombesin and radiolabelled with lutetiumum-177 and yttrium-90.

**Materials and methods:** Nanogels (NG) with controlled properties were radiation-synthesised from water-soluble, biocompatible polymer—poly(acrylic acid)—of two distinct nominal molecular weights (250 kDa and 450 kDa, marked PAA250 and PAA450, respectively). Irradiation of dilute, deoxygenated aqueous solutions of PAA with microsecond pulses of fast electrons from a linear accelerator [1–4] led to the formation of the structures of ca. 50 nm and 90 nm size, respectively. Obtained nanogels were functionalized with the targeting peptide (bombesin) coupled with chelating moiety (1,4,7,10-tetraazacyclododecane-1,4,7,10-tetraacetic acid—DOTA). Radiolabelling of so obtained nanocarriers (NGBD) with [^177^Lu]LuCl3 (LutaPol) and [^90^Y]YCl3 (ItraPol) was performed by mixing 0.5 mg of NGBD (1 mg/mL) with 0.2 mL of ascorbic acid sodium salt solution of pH = 4.5–5.0, and 2–100 μL of the radionuclide. The sample was incubated at 95 ± 5 °C for 15 min. The final formulation's RCY (radiochemical yield) was determined by thin-layer chromatography on glass fibre silica-gel coated plates (ITLC SG) with 0.2 M potassium chloride pH = 2.0–2.5 as a mobile phase [5]. The biological activity of radiolabeled NGBD was proven based on in vitro (uptake of nanocarriers by Human prostate adenocarcinoma cell line PC-3) and in vivo studies (pharmacokinetics in BALB/c mice).

**Results:** As a result of optimized labelling conditions, we achieved a nanocarrier system characterised by high radiochemical yield (> 98%) and good stability, both in human serum and labelling buffer, up to 14 days. Furthermore, the in vitro studies showed that the application of nanocarrier substantially increased radioisotope internalisation in prostate cancer cells and this effect is mediated specifically by the presence of the targeting ligand conjugated to the carrier. The in vivo studies revealed, that both types of NGBD were mainly deposed in the liver. The uptake of nanogels based on PAA450 was 90.2 ± 12.4% ID/g at 2 h p.i.v. and 26.9 ± 4.8% ID/g at 7 days, while PAA250 NGBD uptake was 67.8 ± 9.74% ID/g at 2 h p.i.v. and 18.8 ± 1.8% ID/g at 7 days which clearly indicate that the size of nanoparticles influences biodistribution. The femur uptake was constant for all 7 days, indicating the stability of particles in vivo, and equal to ca. 2%ID/g. We also observed very slow elimination in the urine (ca. 12% ID at 28 h), which consequences a low kidney burden not exceeding 2% ID/g.

**Conclusion:** The results of the study render NGBD a promising ligand for theranostic application. It provides a proof of concept for preparing radiation-synthesised nanogel and paves the way for its further development and pre-clinical use. Further study will be focused on optimization the size of particles that should decrease liver accumulation and on the increase of DOTA substitution which will influence the specific activity of particles.

This work is supported by the National Science Centre, Poland (Grant no. 2019/33/B/ST5/02125).

**References**

1. Ulanski P, Janik I, Rosiak JM. Radiat Phys Chem. 1998;52:289–94.
2. Kadlubowski S, Grobelny J, Olejniczak W, Cichomski M, Ulanski P. Macromolecules 2003;36:2484–92.
3. Kadlubowski S, Ulanski P, Rosiak JM. Polymer (Guildf). 2012;53:1985–91.
4. Jeszka JK, Kadlubowski S, Ulanski P. Macromolecules 2006;39:857–70.
5. Matusiak M, Rurarz BP, Kadłubowski S, Wolszczak M, Karczmarczyk U, Maurin M, Kolesińska B, Ulański P. Pharmaceutics 2021;13:1240.

## PP02

### Automated synthesis of [^68^Ga]Ga-DOTA-Exendin-4 via fractioned ^68^Ge/^68^Ga generator elution

#### Martin Kraihammer^1,2^, Elisabeth von Guggenberg^1^, Michael Gabriel^2^, Christian Uprimny^1^, Clemens Decristoforo^1^

##### ^1^Department of Nuclear Medicine, Medical University of Innsbruck, Innsbruck, Austria. ^2^Department of Nuclear Medicine and Endocrinology, Kepler University Hospital, Linz, Austria

*EJNMMI Radiopharmacy and Chemistry* 2023; 8(Suppl1): PP02

**Aim:** Imaging of insulinoma—a rare subtype of pancreatic neuroendocrine tumors—is a crucial step in the treatment of this disease, as it allows precise surgical excision of the tumorous tissue. PET/CT imaging with ^68^Ga-labelled radiopharmaceuticals targeting Glucagon-like peptide-1 (GLP-1) receptors has shown promising results (1).

The data we present here, describes the automated synthesis of [^68^Ga][Nle14,Lys40(Ahx-DOTA-Ga)NH2]exendin-4 (abbreviated as [^68^Ga]Ga-DOTA-exendin-4), an analogue of GLP-1 with longer biological half-life.

**Materials and methods:** For automated synthesis a Modular-Lab PharmTracer module (Eckert &and Ziegler Radiopharma GmbH, Germany) with a customized synthesis method was used. ^68^Ga was eluted from a GalliaPharm ^68^Ge/^68^Ga generator (Eckert and Ziegler Radiopharma GmbH, Germany). Fractioned elution of the generator was performed, after unsuccessful labelling tests with strong cation exchange (SCX) cartridge pre-purification of ^68^Ga. For radiolabelling, the main ^68^Ga fraction was mixed and heated with 40 µg of DOTA-exendin-4 followed by solid phase extraction purification (SPE) on a C18 cartridge and sterile filtration.

Quality control of the final product including ITLC and RP-HPLC was performed and validated. Stability testing over a period of 4 h was conducted as well.

**Results:** Radiochemical yield as determined by radio-HPLC was 50 ± 5.8% (values decay-corrected to end of synthesis, mean ± SD, n = 5) and specific activity was 26 ± 2.6 MBq/µg (at end of synthesis, mean ± SD, n = 5). For high recovery of the product, addition of 0.1% polysorbate 80 solution to the synthesis process was required.

Radiochemical purity as determined by radio-HPLC was 98% for all batches (n = 5). Quality control for sterility, filter integrity, endotoxin content, ethanol content, radionuclidic purity and identity, pH and appearance were all in line with pre-defined specifications, which were set based on existing Ph.Eur. monographs for ^68^Ga-labelled radiopharmaceuticals.

**Conclusion:** The method described here for automated production of [^68^Ga]Ga-DOTA-exendin-4 yields good, reproducible results with sufficient specific activity for clinical application. The proposed specifications for the labelled product are conforming to current Ph.Eur. monographs for ^68^Ga-labelled radiopharmaceuticals.

**Reference**

1. Luo Y, Pan Q, Yao S, et al. Glucagon-like peptide-1 receptor PET/CT with ^68^Ga-NOTA-exendin-4 for detecting localized insulinoma: a prospective cohort study. J Nucl Med. 2016;57(5):715–20.

## PP03

### The changes of the plaque inflammation assessed by F-18 FDG PET after radioimmunotherapy with I-131 rituximab in patients with lymphoma

#### Byeong Hyeon Byeon^1^, Sang Moo Lim^1^, Hye Jin Kang^2^

##### ^1^Department of Nuclear Medicine, Korea Cancer Center Hospital, Korea Institute of Radiological and Medical Sciences, Seoul, Korea. ^2^Division of Hematology/Oncology, Department of Internal Medicine, Korea Cancer Center Hospital, Korea Institute of Radiological and Medical Sciences, Seoul, Korea

*EJNMMI Radiopharmacy and Chemistry* 2023; 8(Suppl1): PP03

**Aim:** Atherosclerosis is a chronic inflammatory disease modulated by many immune cells, including B cells. Preclinical and several small-scale human studies have reported the reduction of plaque inflammation by using rituximab which depletes atherogenic B-2 cells. We assessed the influence of radioimmunotherapy with I-131 rituximab on the plaque inflammation assessed by F-18 FDG PET in patients with lymphoma.

**Materials and methods:** We retrospectively analyzed F-18 FDG PET scans of patients treated with I-131 rituximab for the first time for relapsed or refractory CD20(+) B cell lymphoma. F-18 FDG PET scans were performed before (PET1), 5 days (PET2), and 1 m (PET3) after the administration of I-131 rituximab. On each PET dataset, target-to-background ratio (TBR) was calculated from the ratio of maximum standardized uptake value of the right carotid artery compared with mean standardized uptake value of inferior vena cava. TBRs measured in PET1, PET2, and PET3 were designated as TBR1, TBR2, and TBR3, respectively. Statistical analysis was performed using the Wilcoxon-test for comparison between the groups. In addition, we divided patients into 2 groups (patients with higher TBR1 versus equal to or lower TBR1 than the median value of TBR1) and compared TBRs on each PET dataset.

**Results:** A total of 32 patients were analyzed for 32 radioimmunotherapies. The mean and SD values of TBR1, TBR2, and TBR3 were 1.49 ± 0.28, 1.36 ± 0.21, and 1.35 ± 0.19, respectively. TBR2 (*P* = 0.008) or TBR3 (*P* = 0.002) was statistically significantly lower than TBR1, while there was no significant difference between TBR2 and TBR3. In patients with higher TBR1 (n = 16), TBR significantly decreased in PET2 (mean TBR = 1.46, *P* = 0.001) or PET3 (mean TBR = 1.42, *P* < 0.001) compared with TBR in PET1 (mean TBR = 1.69). In patients with lower TBR1 (n = 16), however, TBR in PET2 (mean TBR = 1.26) nor PET3 (mean TBR = 1.29) showed significant difference compared with TBR in PET1 (mean TBR = 1.29).

**Conclusion:** In lymphoma patients with higher plaque inflammation, the degree of plaque inflammation measured by F-18 FDG PET significantly decreased after radioimmunotherapy with I-131 rituximab. On the other hand, in patients with lymphoma with lower plaque inflammation, no significant decrease in plaque inflammation was observed after radioimmunotherapy. It is presumed that plaque inflammation was alleviated by B cell depletion by radioimmunotherapy with I-131 rituximab.

**References**

1. Lee I, Byun BH, Lim I, Kim BI, Choi CW, Kim KM, Shin DY, Kang HJ, Lim SM. Comparisons of ^131^I-rituximab treatment responses in patients with aggressive lymphoma and indolent lymphoma. Ann Nucl Med. 2019;33(12):881–90.
2. Shin DY, Byun BH, Kim KM, Kang JH, Lim I, Kim BI, Lee SS, Choi CW, Kang HJ, Lim SM. Radioimmunotherapy with (^131^)I-rituximab as consolidation therapy for patients with diffuse large B-cell lymphoma. Cancer Chemother Pharmacol. 2016;78(4):825–31
3. Lim I, Park JY, Kang HJ, Hwang JP, Lee SS, Kim KM, Choi TH, Yang SH, Kim BI, Choi CW, Lim SM. Prognostic significance of pretreatment ^18^F-FDG PET/CT in patients with relapsed/refractory B-cell non-Hodgkin's lymphoma treated by radioimmunotherapy using ^131^I-rituximab. Acta Haematol. 2013;130(2):74–82.
4. Kang HJ, Lee SS, Byun BH, Kim KM, Lim I, Choi CW, Suh C, Kim WS, Nam SH, Lee SI, Eom HS, Shin DY, Lim SM. Repeated radioimmunotherapy with ^131^I-rituximab for patients with low-grade and aggressive relapsed or refractory B cell non-Hodgkin lymphoma. Cancer Chemother Pharmacol. 2013;71(4):945–53.

## PP04

### Development of freeze-dried kit for one-step expeditious preparation of [^99m^Tc]Tc-PSMA-T4

#### Wioletta Wojdowska^1^, Barbara Janota^1^, Marcin Radzik^1^, Agnieszka Sawicka^1^, Justyna Pijarowska-Kruszyna^1^, Antoni Jaroń^1^, Michał Maurin^1^, Piotr Garnuszek^1^

##### ^1^National Centre for Nuclear Research Radioisotope Centre POLATOM, Otwock, Poland

*EJNMMI Radiopharmacy and Chemistry* 2023; 8(Suppl1): PP04

**Aim:** The purpose of this study was to develop a kit formulation for radiopharmaceutical preparation of [^99m^Tc]Tc-PSMA-T4.

**Materials and methods:** The kit formulation was optimized using different amounts of the active substance and excipients and under different labelling conditions. Influence of PSMA-T4, EDDA, tricine and SnCl_2_ × 2H_2_O content, tricine/EDDA ratio, activity and volume of ^99m^Tc-pertechnetate eluate and the time of heating on radiochemical purity of [^99m^Tc]Tc-PSMA-T4 was tested using LC–MS and HPLC. To confirm the structure of the final radiopharmaceutical preparation, the ^99m^Tc/^99^Tc-EDDA/PSMA-T4 complex was synthesized via tricine/EDDA co-ligand exchange reaction. The complex was analyzed using LC-MS. The quality control and stability testing of the kit formulation and the resulting radiolabelled compound were performed by HPLC and TLC analysis.

**Results:** Several different developmental batches of PSMA-T4 kits were prepared, which varied in parameters including the content of PSMA-T4 (5–30 μg) and excipients: EDDA, tricine, SnCl_2_ × 2H_2_O and also tricine/EDDA ratio. The final formulations contained 23 ug PSMA-T4 (net), 50 μg SnCl_2_ × 2H_2_O, 50 mg tricine, 5 mg EDDA, 29 mg Na_2_HPO_4_ × 12H_2_O and 3 mg NaH_2_PO_4_ × 2H_2_O. A few batches were prepared under GMP conditions and tested extensively regarding defined specification. The radiolabelling performed by incubation of 300–1000 MBq of ^99m^Tc[Tc]O4·at 100 °C for 15 min resulted in reproducible radiochemical purity (RCP) > 91%. Kit-stability was proven for 12 months at 2–8 °C. The radioactive preparation was stable for > 4 h at 25 °C.

**Conclusion:** The kit formulation for convenient preparation of [^99m^Tc]Tc-PSMA-T4 for clinical application was developed, showing high stability of the kit as well as high RCP of the final radioactive preparation.

**References**

1. Liu S, Edwards DS. Chem Rev. 199;99(9):2235–68. https://doi.org/10.1021/cr980436l.
2. Liu S, Edwards DS, Looby RJ, Harris AR, Poirier MJ, Barrett JA, Heminway SJ, Carroll TR. Bioconjug Chem. 1996;7(1):63–71. https://doi.org/10.1021/bc950069.

## PP05

### Synthesis and radiolabeling of designed HYNIC-GPLGAAD with ^99m^Tc for fibrin imaging in invivo

#### Sara Hadadi^1^*, Soraya Shahhosseini^1^, Sedigheh Rezaeianpour^2^

##### *Correspondence: Sara.hadadi94@yahoo.com

##### ^1^School of Pharmacy, Shahid Beheshti University of Medical Sciences, Tehran, Iran. ^2^Department of Chemistry, North Tehran Branch of Islamic Azad University, Tehran, Iran

*EJNMMI Radiopharmacy and Chemistry* 2023; 8(Suppl1): PP05

**Aim:** Today, Thrombosis is a serious risk factor for both thromboembolic disease (such as DVT, and PE) and cancer. Fibrin is a major constituent of clots and is present in all types of thrombi, but absent in circulation. Fibrin is a highly sensitive and specific target for molecular imaging of thrombi. In the present study, we have developed a linear fibrin-binding peptide for thrombus imaging. The peptide was synthesized, and radiolabeled with ^99m^-Tc, and its stability in normal saline and human plasma and LogP was determined.

**Materials and methods:** The HYNIC-Gly-Pro-Lys-Gly-Ala-Ala-Asp peptide was synthesized using a standard Fmoc strategy and radiolabeled with ^99m^-Tc. The stability of the radiolabeled peptide in human plasma and normal saline as well as partition coefficient (LogP) was determined. The effect of the peptide on platelet aggregation and the percent of binding radiopeptide to fibrin were studied.

**Results:** HYNIC-GPLGAAD calculated forC31H47N11O11:749.3;found m/z = 750.3 [M + H]+. Analytical RP-HPLC: Rt = 8 min ; 85% A, 15% B, Log*P* = − 2.58 ± 0.51

The optimal labeling conditions were 20 μg peptide, 80 °C, pH 5–6, and incubation time of 30 min. The radiochemical purity of the radiolabeled peptide was more than 95%. The stability of peptide in human plasma at 37 °C was 95 % after 6 h. peptide had no effect on platelet aggregation. fibrin binding of Radiopeptide was 71.36%.

**Conclusion:** The results indicate that peptide is a promising agent for in vitro and in vivo studies.

**References**

1. Felber M, Alberto R. ^99m^Tc radiolabelling of Fe_3_O_4_–Au core-shell and Au–Fe_3_O_4_ dumbbell-like nanoparticles†. 2015.
2. Chung EJ, Cheng Y, Morshed R, Lesniak MS, Tirrell MV, et al. Fibrin binding peptide amphiphile micelles for targeting glioblastoma. Biomaterials. 2014;15:1249–59.
3. Kongsuphol P, Arya SK, Wong CC, Polla LJ, Park MK. Coiled-coil peptide-based sensor for ultra-sensitive thrombin detection. Biosens Bioelectron. 2014;55:26–31.
4. Starmans LWE, Duijnboven SMJV, Rossin R, et al. Evaluation of ^111^In-labeled EPep and FibPep as tracers for fibrin. Mol Pharm. 2013;10:4309–21.
5. Ciesienski KL, Yang Y, et al. Fibrin targeted PET probes for the detection of thrombi. Mol Pharm. 2013;10:1100–10.

## PP06

### Novel hybrid imaging probes targeting the CCK2 receptor by chelator scaffolding

#### G. Gariglio^1^, D. Putzer^2^, E. von Guggenberg^1^, M. Zierke^1^, A. Hörmann^1^, C. Decristoforo^1^

##### ^1^Department of Nuclear Medicine, Medical University Innsbruck, Innsbruck, Austria. ^2^Department of Radiology, Medical University Innsbruck, Innsbruck, Austria

*EJNMMI Radiopharmacy and Chemistry* 2023; 8(Suppl1): PP06

**Aim:** The outcome of surgical tumor resection benefits by preoperative imaging and intraoperative real-time guidance.

Dual-modality probes, combining positron emission tomography (PET) with fluorescence imaging (FI) capabilities in the same molecule, can be used for both applications and therefore are of high clinical relevance [1, 2], in particular if the probes are specifically targeted towards the tumor.

In this project we aim to synthesize dual-labeled probes targeting the CCK2 receptor. They are based on different cyclic scaffolds modified with Minigastrin peptides, as a model targeting moiety, and a fluorescent dye.

**Materials and methods:** The cyclic chelators TRAP-Pr and Fusarinine C (FSC) were chosen as scaffolds. The synthetic approach is based on a first modification to enable click bioconjugation chemistry. This is followed by coupling a fluorescent dye and an azide modified peptide sequence. Finally, the coordinated metals are removed. Characterization is performed by HPLC and MS.

The compounds will initially be characterized in vitro regarding their ^68^Ga radiolabelling and stability properties, continuing with binding studies with CCK2R expressing cell lines.

Then, the most promising compounds will be investigated in normal animals regarding biodistribution and eventually in animal tumor models for targeting and imaging studies using both PET and optical imaging.

**Results:** As initial proof of principle, TRAP-Pr scaffold was successfully coupled with the Sulfo-cyanine5.5 dye and then conjugated to minigastrin5-(PEG)4-N3 by Cu(I) catalyzed azide-alkyne cycloaddition (CuAAC). After removal of copper, the homodivalent dual-modality imaging agent Sulfo-Cy5.5-TRAP(MGS5)2 was obtained and characterized by HPLC and MS. Initial radiolabelling studies revealed successful radiolabelling at high molar activities.

Further synthesis of the corresponding FSC-analog and initial preclinical evaluation of the studied compounds are currently ongoing.

**Conclusion:** The preliminary results show that the preparation of dual-labeled imaging probes based on different chelating scaffolds is feasible.

Following studies will provide data about the applicability of the prepared compounds.

**References**

1. Yuen R, et al. Dual probes for positron emission tomography (PET) and fluorescence imaging (FI) of cancer. Pharmaceutics. 2022;14:645.
2. Summer D, et al. Developing targeted hybrid imaging probes by chelator scaffolding. Bioconjug Chem. 2017;28(6):1722–33.

## PP07

### A 67/64Cu-mixture as a therapeutic alternative to pure 67Cu

#### L. De Nardo^1,2^, G. Pupillo^3^, L. Mou^3^, J. Esposito^3^, A. Rosato^4,5^, L. Meléndez-Alafort^4^

##### ^1^DFA, University of Padua, Padua. ^2^INFN-Padua, Padua. ^3^INFN-LNL, Legnaro (Padua). ^4^IOV-IRCCS, Padua. ^5^DISCOG, University of Padua, Padua

*EJNMMI Radiopharmacy and Chemistry* 2023; 8(Suppl1): PP07

**Aim:** Two copper radioisotopes, 64Cu and 67Cu, are currently considered among the most promising radionuclides for both diagnosis and therapy of cancers. 64Cu radionuclide is already commercially available, as it can be produced with high specific activity by using proton beams available at low energy (i.e. 18–24 MeV), so called medical, cyclotrons. On the other hand, high yield production of 67Cu is difficult, due to the co-production of other Cu-isotopes, especially 64Cu. To address this issue, currently preventing the spread use of 67Cu in preclinical as well as clinical research programs, the possibility of using a mixture of 64Cu and 67Cu radioisotopes for therapeutic applications has been considered in this work.

**Materials and methods:** Copper radioisotopes yields were calculated by considering proton beam irradiation of both ^70^Zn and ^68^Zn targets under different energy ranges and irradiation times. A simple spherical model, representing tumours of different sizes, was used to calculate the absorbed dose due to the self-irradiation for a uniformly distributed 67/64Cu mixture. The biokinetic model for CuCl_2_ published by ICRP 53 [1] was used to assess the human absorbed dose to healthy organs due to the 67/64Cu mixture with the OLINDA software [2].

**Results:** By comparing the absorbed doses to a sphere model due to uniformly distributed 64Cu and 67Cu, it was found that 64Cu administered activity must be about five times higher than that of 67Cu to obtain the same absorbed dose for tumour mass 0.01–10 g and about ten times higher for smaller ones. By administration of a 67/64Cu mixture, a supplemental activity is therefore required to get the same tumour absorbed dose produced by pure 67Cu. This supplemental activity, triggering a dose increment in healthy organs, depends on the time of injection of the 67/64CuCl_2_ mixture, decreasing with increasing time post the end of the bombardment (EOB), due to the increasing 67Cu radionuclide fraction in the mixture.

**Conclusion:** A mixture of 67/64Cu radioisotopes could impart the same tumour absorbed dose as that due to pure 67Cu, with a minimal (99%) 67Cu, obtained after waiting times after the EOB sufficiently long (120–145 h) to allow 64Cu decay [3].

**References**

1. ICRP Publication 53. Ann ICRP. 1988;18(1–4).
2. Stabin M, Farmer A. J Nucl Med. 2012;53(1):585.
3. De Nardo L, et al. Med Phys. 2022;49:2709–24.

## PP08

### Fully-automated GMP production of [^18^F]AlF-NOTA-octreotide on a Trasis AiO module

#### F. Trejo-Ballado, E. Zamora-Romo, H. Gama-Romero, M. Mendoza-Figueroa, R. Tecuapetla-Chantes, U. Rabadán-Domínguez, G. Contreras-Castanon, D. Bolanos-Perez, A. Zarate-Morales, A. Flores-Moreno, M.A. Avila-Rodriguez

##### Unidad Radiofarmacia-Ciclotrón, Facultad de Medicina, Universidad Nacional Autónoma de México, CDMX, 04510, MÉXICO

*EJNMMI Radiopharmacy and Chemistry* 2023; 8(Suppl1): PP08

**Aim:** Gallium 68 labelled somatostatin analogues are the standard for somatostatin receptor PET imaging of neuroendocrine tumors (NET). [^18^F]AlF-NOTA-Octreotide ([^18^F]FOC) is a fluorine labelled alternative with satisfactory biodistribution and dosimetry profiles with high NET lesion detection rate (Long et al., 2019). The goal of this work was to implement the fully automated production of [^18^F]FOC in a Trasis AllinOne module for routine clinical production.

**Materials and methods:** Synthesis was performed via chelation of the chemical precursor NOTA-Octreotide trifluoroacetate (ABX GmbH, Germany) with aluminum-[^18^F] fluoride ([^18^F]AlF), using a commercial PSMA-1007 cassette with minor changes. Briefly, [^18^F] fluoride ion (^18^F]F-) was trapped in a QMA cartridge and washed with 10 mL of WFI. Then the [^18^F] F- is eluted with 0.6 mL of 0.9% NaCl to the reactor vessel preloaded with 60 µL AlCl_3_ 2 mM + 30 µL sodium ascorbate 0.1 M. After incubation for 3 min at 60 °C, 200 µg of chemical precursor in 270 µL sodium acetate buffer (0.1 M, pH 4.1) + 700 mL EtOH were added to the reaction vessel and heated at 100 °C for 10 min. The reaction mixture was diluted with 8 mL of formulation solution (NaAsc 0.59% in NaCl 0.9% in WFI) and purified via SPE on a Sep-Pak C18 light cartridge. After rinsing with 15 mL of formulation solution, [^18^F]FOC was eluted with 1.5 mL of EtOH, diluted with 25 mL of formulation solution, and sterilized by filtration (0.22 µm Sartorius Minisart).

**Results:** Using an initial activity of 140 ± 10 GBq of [^18^F]F-, [^18^F]FOC synthesis was satisfactorily accomplished obtaining yields of 17–20% (ndc) after a synthesis time of 35 min (n = 20), with a radiochemical purity > 98% as determined by analytical HPLC.

**Conclusion:** A convenient and reliable fully automated synthesis of [^18^F]AlF-NOTA-Octreotide was successfully implemented in a cassette-based module as per the GMP standards, and it complied all the quality control tests, obtaining [^18^F]FOC in enough quantity and quality for routine clinical applications. Project supported by UNAM-DGAPA grant PAPIIT-IT200221.

**References**

1. Long et al. Clin Nucl Med. 2019;44:452–8.

## PP09

### The key steps to prepare a VHH targeting mesothelin and four derivatives for theranostic studies using ^68^Ga and ^177^Lu

#### M. Ahmadi, S. Bacot, T. Guenard, F. Raes, E. NGuessan, C. Lombardi, A. Broisat C. Ghezzi

##### Université Grenoble Alpes, Laboratoire Radiopharmaceutiques Biocliniques (LRB), Inserm, CHU Grenoble Alpes, 38000 Grenoble, France

*EJNMMI Radiopharmacy and Chemistry* 2023; 8(Suppl1): PP09

**Aim:** During the past decade, many biomolecules have been developed as theranostic agents using the ^68^Ga and ^177^Lu pair for their radiolabeling. In order to develop a suitable radiolabeled molecule, different parameters should be considered. The control of these parameters is challenging when the vector molecule is a protein associated with a DOTA chelating agent. This chelating agent is characterized by slow kinetic and therefore requires heating. In this study the key steps required for the radiolabeling of a single-domain antibody (VHH) with the Ga/Lu pair, using DOTA, have been identified and evaluated.

**Materials and methods:** A VHH targeting mesothelin^1^ and four derivatives were designed. Firstly thermal stability was studied by Circular Dichroism. VHHs were then conjugated with p-SCN-Bn-DOTA via the primary amine side chain of lysines^2^. Different conditions of molar ratio, pH and incubation time were studied. After identification of suitable conjugated VHH by Mass Spectrometry analysis, radiolabeling parameters with Ga were determined. The effect of buffer and VHH concentration on conjugation stability were also studied.

**Results:** In order to prepare an optimal radiolabelled VHH, three key steps were identified. (a) Thermal stability study: The VHH and its derivatives were heated at 60 °C without secondary structure changes. (b) Conjugation study and MS analysis: The best conjugation conditions were identified with MS analyses, based on the absence of unconjugated VHH residue. A molar ratio of 50:1 (DOTA-VHH) at a pH 8.5–9 was required. (c) Radiolabeling adjustment: 6 nmol of each VHH (0.2–1 mg/mL) in ammonium acetate 0.1 M was radiolabeled with a very good radiochemical purity (> 90%) at pH 3–4, in a final volume of 1 mL. The radiolabeled products were stable at least 2 h after radiolabeling.

**Conclusion:** Identifications of key steps in the radiolabeling procedure of VHH with Ga and/or Lu, led to a high-efficiency-standardised preparation of the radiolabeled theranostic agents.

These steps can be applied as a standard procedure for the development of any VHH radiolabeled with ^68^Ga, ^177^Lu or many of other trivalent radioisotopes such as 161-Tb or 64-Cu.

**References**

1. Montemagno C, Bacot S, Ahmadi M, Kerfelec B, Baty D, Debiossat M, Soubies A, Perret P, Riou L, Fagret D, Broisat A, Ghezzi C. Preclinical evaluation of mesothelin-specific ligands for SPECT imaging of triple-negative breast cancer. J Nucl Med, 2018;59(7):1056–62.
2. Mueller B, Wrasidlo W, Reisfeld R. Determination of the number of e-amino groups available for conjugation of effector molecules to monoclonal antibodies. Hybridoma. 1988;7:453–56.

## PP10

### Automated synthesis for ^18^F-labelled norepinerphrine transporter tracer [^18^F]NS12137 using trasis allinone

#### Noora Rajala^1^, Anna K. Kirjavainen^1,2^, Salla Lahdenpohja^1,3^

##### ^1^Radiopharmaceutical chemistry labortory, Turku PET Centre, University of Turku, Turku, Finland. ^2^Department of Chemistry, University of Turku, Turku, Finland. ^3^Service Hospitalier Frédéric Joliot, CEA, Orsay, France

*EJNMMI Radiopharmacy and Chemistry* 2023; 8(Suppl1): PP10

**Aim:** The norepinephrine transporter (NET) is a monoamine transporter, which has an important role in several neuropshyciatric disorders, such as ADHD and schizophrenia.^1^ Our aim is to develop a cassette-based synthesis for ^18^F-fluorination 3-[(6-[^18^F]fluoro-2-pyridyl)oxy]-8-azabicyclo[3.2.1]octane ([^18^F]NS12137) using the TRASIS AllInOne module.

**Materials and methods:** [^18^F]NS12137 was synthesized using nucleophilic substitution of a brominated precursor. [^18^F]fluoride was loaded in preconditioned QMA cartridge and eluted with a solution of K_2_CO_3_/K222/H_2_O/MeCN. Subsequently, MeCN was evaporated at 110 °C with N_2_ flow and vacuum. The precursor was added to the reaction vial in DMSO and the reaction was carried out at 185 °C for 15 min. The DMSO was evaporated at 185 °C with N_2_ flow and vacuum. Deprotection was achieved using 48% HBr and heating at 50 °C for 5 min. [^18^F]NS12137 was purified by semi-preparative radio-HPLC using a gradient with 7 mM KH_2_PO_4_ and MeCN as eluents. The purified product fraction was formulated using SPE cartridge. [^18^F]NS12137 was eluted from the cartridge using EtOH and saline. The chemical and radiochemical purity of the final product was analyzed using analytical radio-HPLC.

**Results:** The radiochemical yield of formulated product was 12% calculated from initial [^18^F]fluoride with 60 min synthesis time. The activity of the end product was 820 MBq EOS. The radiochemical purity of the product was 99% determined by analytical radio-HPLC.

**Conclusion:** [^18^F]NS12137, was successfully synthesized in good yield using nucleophilic fluorination. More optimization tests are needed for the evaporation of DMSO and HPLC purification.

**Reference**

1. López-Picón FR, Kirjavainen AK, Forsback S, Takkinen JS, Peters D, Haaparanta-Solin M, Solin O. In vivo characterization of a novel norepinephrine transporter PET tracer [^18^F]NS12137 in adult and immature Sprague-Dawley rats. Theranostics. 2019;9(1):11–9.

## PP11

### Aluminum-[^18^F]fluoride radiolabeling optimization by design of experiments approach

#### Carine San^1,2^, Meriem Beddek^2^, Fabienne Dioury^1^, Nicolas Vignal^2^, Benoît Hosten^2^, Maurice Pillet^4^, Laure Sarda Mantel^3^, Marc Port^1^

##### ^1^Conservatoire National des Arts et Métiers (Le Cnam), Laboratoire Génomique, Bioinformatique, Chimie Moléculaire (GBCM), équipe Chimie Moléculaire, Paris, France. ^2^Assistance Publique - Hôpitaux de Paris - Université Paris Cité, Unité Claude Kellershohn, Institut de Recherche Saint-Louis, hôpital Saint-Louis, Paris, France. ^3^Assistance Publique - Hôpitaux de Paris - Université Paris Cité, Service de Médecine Nucléaire, hôpital Lariboisière, Paris, France. ^4^Laboratoire SYMME, EA 4144, Université Savoie Mont-Blanc, Annecy, France

*EJNMMI Radiopharmacy and Chemistry* 2023; 8(Suppl1): PP11

**Aim:** Fluorine-18 (^18^F) is the most favorable positron emitter for tumor imaging. However, direct ^18^F labeling of biomolecules is challenging as it involves multiple steps and stringent conditions that are generally not suitable for biomolecules whose integrity may be altered. Over the past decade, an elegant new approach has been developed by coordination of aluminum-[^18^F]fluoride (Al-^18^F)^1^. The objective of this work was to optimize Al-^18^F radiolabeling by using a design of experiments (DoE) approach.

**Materials and methods:** A NODA derivative bearing a thiourea function (NODA-MP-C4) was prepared as a model chelator. [^18^F]fluoride was trapped in a QMA cartridge and eluted with 1.8 mL of NaCl. Al-^18^F radiolabeling was manually performed by adding an aliquot of 500 MBq of Na[^18^F]F, 30 µL of 2 mM AlCl_3_ and 30 µL of 2 mM NODA to the reaction vessel. After a reaction time of 15 min at 100 °C, the crude mixture was analyzed with radio-HPLC to determine the radiolabeling yield. To optimize this yield, a DoE was designed and analyzed using Ellistat® software (v6.8). Based on numerous reported results and our previous work^2-4^, three experimental factors were investigated: pH, NODA-MP-C4 concentration ([NODA]) and NODA/aluminum ratio (NODA/Al).

**Results:** The DoE study consisted of 20 duplicate experiments over 5 days of Na[^18^F]F production. Results showed all factors investigated have significant effects on radiolabeling yield. According to the DoE results, the optimal set of conditions was: pH = 4.5, [NODA] = 465 µM, and NODA/Al = 1:1. These conditions were tested in triplicate and gave respectively 80.4%, 75.9% and 79.1% of radiolabeling yield [78.5% ± 2.3% (n = 3)], demonstrating the robustness of the study, and giving the highest radiolabeling yield obtained so far.

**Conclusion:** Radiolabeling optimization using a DoE approach was achieved with a satisfactory radiolabeling yield while reducing the number of experiments and improving worker radioprotection. This approach will be used for our future radiofluorination strategies.

**References**

1. McBride, et al. J Nucl Med. 2009;50(6):991–8.
2. Fersing C, et al. Molecules. 2019;24(16):2866.
3. Shetty D, et al. Bioorg Med Chem. 2012;20(19):5941–47.
4. San C, et al. 28th young research fellows meeting, Paris. 2021.

## PP12

### Detectors and reactors for microfluidic synthesis of radiopharmaceuticals

#### L. Fernandez-Maza^1,3^, B. Salvador^2^, A. Corral^1^, D. Orta^1^, A. Luque^2^

##### ^1^Centro Nacional de Aceleradores. Universidad de Sevilla. ^2^Escuela Tecnica Superior de Ingenieria. Universidad de Sevilla. ^3^Hospital Punta de Europa. Algeciras

*EJNMMI Radiopharmacy and Chemistry* 2023; 8(Suppl1): PP12

**Aim:** The purpose of the study was to test and integrate two different components of a lab-on-chip for microfluidic synthesis of radiopharmaceuticals; radiation detectors to monitor the synthesis process, and optimization of reactor chamber.

**Materials and methods:** Silicon photomultipliers (SiPM) were tested to monitor the flow of radioactivity in different parts of the chip. An array of 16 SiPM sensors was placed next to the surface of the chip, covering the different parts where we wanted to monitor the flow of radioactivity.

Polydimethylsiloxane (PDMS) compatibility with [^18^F]Fluoride was studied for the manufacture of microfluidic reactors to prepare radiopharmaceuticals. We also tried to optimize the chamber volume and the top PDMS membrane for evaporations.

Chips and elution product radioactivity were measured in a dose calibrator after every synthesis step.

**Results:** A matrix of 4 × 4 P-on-N sensors of 3 × 3 mm each and separation of 200 μm covered QMA resin chamber and reactor chamber, allowed the monitoring of the flow of radioactivity, from QMA resin to the reaction chamber, and subsequently to the purification resin chamber. Once eluted from the chip, no remaining activity was measured in the cartridges. [^18^F]Fluoride was collected in a vial at the activimeter, recovering all radioactivity, with the decay correction.

Volume of 50 μL gave good results at heating processes, avoiding the breakage of the chamber. Thickness of 50 μm for the top PDMS membrane allowed complete evaporation, without leakage or breakage.

**Conclusion:** SiPMs allow control of radioactivity levels in the synthesis stages, such as preconcentration of F-18 in QMA resin, arrival of radioactivity at the reactor, nucleophilic substitution, protective groups hydrolysis and solvent evaporations.

After subjecting PDMS to the F-18, heating and vacuum normally used in conventional synthesis, we concluded that [^18^F]Fluoride does not adsorb and elutes almost entirely, making PDMS highly suitable for single-use radiopharmaceutical chips due to its low cost, F-18 compatibility and GMP compliance for injectable drugs.

**References**

1. Salvador B, et al. IEEE Sens J. 2019;17:7702.
2. Fernandez-Maza L, et al. Microfluidics Nanofluidics. 2019;23:109.

## PP13

### In vitro stability studies of ^111^In-DTPA-FITC-silk fibroin nanoparticles

#### Angela Alonso Garcia^1^, M Alejandra Asensio Ruiz^1,3^ Luz Bravo Moreno^1^, A A Lozano Pérez^2^, M T Martínez Martínez^1^

##### ^1^Unidad de Radiofarmacia. Hospital Clínico Universitario Virgen de la Arrixaca, Murcia, Spain. ^2^Departamento de Biotecnología, Instituto Murciano de Investigación y Desarrollo Agrario y Alimentario (IMIDA), Murcia, Spain. ^3^Grupo de Radiofarmacia. Instituto Murciano de Investigación Biosanitaria IMIB-Arrixaca. 30120 Murcia, Spain.

*EJNMMI Radiopharmacy and Chemistry* 2023; 8(Suppl1): PP13

**Aim:** In the last decades, application of Silk Fibroin Nanoparticles (SFNs) for drug delivery has notably increased. In the field of new drug development, image guided technique is playing an increasingly important role in the investigation of the biodistribution and pharmacokinetics. In this sense, an approach of dual labeled (fluorescent and radioactive) Silk Fibroin Nanoparticles (SFNs) for biodistribution studies has been developed in our laboratory. The aim of this work is the in vitro stability study of the ^111^In-DTPA-FITC-SFNs in aqueous matrix and human serum, to verify the suitability for injectable formulation and in vivo studies.

**Materials and methods:** The stability of the radiolabeled particles has been evaluated in water for injection (WFI), sodium chloride 0.9%, phosphate buffered solution (PBS) and serum from human healthy donors. A suspension (n = 12) of 3.28 ± 0.016 MBq/mg of ^111^In -DTPA-FITC-SFNs was introduced in a dialysis membrane tubing (3.5 KDa) and immersed in 20 mL of each medium for 24 h at room temperature. Samples from the external medium were taken at 1, 4, 24 h, quantified in activimeter and analyzed by radiochromatography (RTLC) on ACD-A /silica gel (ITLC-SG) to check percentage of “free” ^111^In (Rf = 0.8–1.0).

In parallel, suspensions (n = 9) of 3.28 ± 0.016 MBq/mg of ^111^In-DTPA-FITC-SFNs were incubated in 2 mL of WFI and human serum for 24 h at 37 °C. Then, they were centrifuged, suspended at 1 mg/mL in NaCl 1 mM for Zaverage (d.nm) and Zpotential (ζ, mV) determination.

Integrity of nanoparticles in suspension was checked before labeling by 1.5% agarose gel electrophoresis.

**Results:** Incubation of radiolabeled particles in WFI, sodium chloride 0.9%, PBS and human serum, showed similar elution percentages (1–5%) with no differences of statistical significance (*P* < 0.05). Radioactivity presented in incubation media was characterized as free ^111^In by RTLC.

Zaverage measurement after 24 h in human serum showed an increase in comparison with WFI, 144.8 ± 3.70 and 131.1 ± 1.22, respectively (*P* 0.05).

Electrophoresis assay showed the presence of peptides in nanoparticles suspensions stored more than 6 months at 2–8 °C.

**Conclusion:** ^111^In-DTPA-FITC-SFNs are stable in WFI and serum, with size and Z potential suitable for cell penetration. The nanoparticles can be stored in suspension ready for radiolabeling at 2–8 °C for 6 months.

**References**

1. Lozano-Pérez AA, Rodriguez-Nogales A, Ortiz-Cullera V, Algieri F, Garrido-Mesa J, Zorrilla P, et al. Int J Nanomed. 2014;9:4507–20.
2. Anna Helbok, Clemens Decristoforo, Georg Dobrozemsky, Christine Rangger, Eric Diederen, Brigitte Stark, Ruth Prassl & Elisabeth von Guggenberg. J Liposome Res. 2010;20(3):219–27.
3. Hong Ding, Fang Wu. Theranostics. 2012;2(11):1040–53.

## PP14

### Actions for increased yields and easier maintenance at the Tracerlab FXC-pro system in the synthesis of L-[11C]methionine

#### Peter Mäding, Jörg Zessin, Martin Kreller, Klaus Kopka, Torsten Kniess

##### Helmholtz-Zentrum Dresden-Rossendorf, Institute of Radiopharmaceutical Cancer Research, Bautzner Landstrasse 400, 01328 Dresden, Germany

*EJNMMI Radiopharmacy and Chemistry* 2023; 8(Suppl1): PP14

**Aim:** L-[11C]Methionine (L-[11C]Met) is frequently used for the diagnosis of tumours located in brain, head and neck or for tumours induced by the multiple myeloma. The radio synthesis of L-[11C]Met commonly starts with [11C]CO_2_ with subsequent transformation to [11C]CH_4_ followed by transfer into [11C]CH_3_I which is used for final labelling of the precursor L-homocystein thiolactone hydrochloride (HCTL) [1, 2]. By performing these steps with the TRACERLab FXC-pro System (GE HC), however, we observed inconstant radiochemical yields and high maintenance efforts especially of the absorber material used within the gas phase iodination. Accordingly, we have searched for optimization of this radio synthesis procedure.

**Materials and methods:** [11C]CO_2_ is produced by the 14N(p,α)11C nuclear reaction with the cyclotron TR-Flex (Advanced Cyclotron Systems Inc.). The radiochemical conversion of [11C]CO2 to [11C]CH4 and [11C]CH3I is performed with the FXC-pro system, using a gas phase iodination in a circle process at 720 °C as the key step. [11C]CH_3_I is transferred via an anion exchange cartridge (OASIS® MAX) which has been loaded with the precursor HTCL beforehand to form L-[11C]Met. L-[11C]Met is eluted from the OASIS cartridge with 0.05 M NaH_2_PO_4_ (pH 4.5) and diluted with 0.9% NaCl solution to obtain an injectable solution. We tested different bed volumes of OASIS cartridges to improve the radiochemical yield of L-[11C]Met. Furthermore, we investigated new absorber materials, i.e. sodium hydroxide on carrier and soda lime with indicator, for the gas phase iodination step for enhancing the maintenance cycles of the FXC-pro system.

**Results:** The radiochemical yields of L-[11C]Met could be increased from 28.5 ± 2.3% (n = 20) to 41 ± 5.2% (n = 19) by using two cartridges (30 mg/30 mg) instead of a single cartridge (30 mg) with constant amount of HTCL precursor (4 mg). Further raising the bed volume (60 mg/30 mg) did not result in improvement of the radiochemical yield (39%). By the optimized method, up to 31 GBq of L-[11C]Met were obtained from 110 GBq [11C]CO_2_ starting activity in 16 min synthesis time. The radiochemical purity of L-[11C]Met was 99%, the average content of HCTL and homocysteine was 0.4 µg/mL and 40 µg/mL in the final injection solution, respectively.

By combining two different absorber materials for the iodination step in separate cartridges (soda lime and sodium hydroxide on carrier), the maintenance interval of the gas phase iodination process was increased to 15 radio syntheses as compared to five before optimization. In this line, the visual inspection of the colour change of the soda lime from white into black colour was helpful to follow the consumption of the absorber material.

**Conclusion:** Optimization of the bed volume of the anion exchange cartridge led to increased radiochemical yields of L-[11C]Met. Extension of the maintenance cycles by using alternative absorber materials as suggested by GE HC (Ascarite) improved the robustness of the TRACERLab FXC pro system in routine production.

**References**

1. Pichler V, Vraka C, Berroterán-Infante N, Krcal A, Eidherr H, Traub-Weidinger T, Hacker M, Mitterhauser M, Wadsak W. Appl Radiat Isot. 2018;141:107.
2. Solbach C, Arndt F, Gratzl R, Beer AJ, Fischer G, Wenz J. Eur J Nucl Med Mol Imaging. 2019;46:S1–725.

## PP16

### Comparison of dosimetric results obtained with different software for the cyclotron produced 47Sc labeling a DOTA-folate radiopharmaceutical

#### L. De Dominicis^1,2^, L. De Nardo^2,3^, L. Meléndez-Alafort^4^, G. Pupillo^1^, L. Mou^1^

##### ^1^Istituto Nazionale di Fisica Nucleare-Laboratori Nazionali di Legnaro (INFN-LNL), Legnaro. ^2^Università degli Studi di Padova, Dipartimento di Fisica e Astronomia, Padova. ^3^INFN-Sezione di Padova, Padova. ^4^Istituto Oncologico Veneto IOV-IRCCS, Padova

*EJNMMI Radiopharmacy and Chemistry* 2023; 8(Suppl1): PP16

**Aim:** Theranostics is a newly emerging field of nuclear medicine combining the therapeutic effect of high LET radiation with the diagnostic imaging suitability of photons, within the administration of a unique radiopharmaceutical. In this context, 47Sc (T 1/2 = 3.3492 d) is considered a promising radionuclide due to its decay emissions consisting of β-particles (E β-mean = 162.0 keV) for small-medium-sized tumors treatment and γ-rays (E γ = 159.381 keV) for SPECT (Single Photon Emission Computed Tomography) imaging. However, the clinical and preclinical employment of 47Sc is still limited due to its scarce availability. While possible production routes are under investigation to find the most favorable ones, the dose of radiation that a patient undergoes after the administration of the cm10 DOTA-folate conjugate labeled with 47Sc produced via the natV(p,x) nuclear reaction is assessed in this work. In the calculations, performed with the IDAC-Dose2.1 (v. 2.1) software [1], also the Sc-contaminants’ contribution to the radiation dose is considered. Results are compared to the outputs previously obtained with the MIRDCalc (v. 1.1) software [2] and with the OLINDA (v. 2.1.1) software [3].

**Materials and methods:** IDAC-Dose2.1 is a voxel phantom-based software. The input data needed by IDAC-Dose2.1 are the cumulated activity (the number of disintegrations) divided by the initial administered activity, for each source organ. These values are derived for human organs from the 47Sc-cm10 biodistribution studies carried out on mice [4], through the use of the relative mass scaling method. The organs’ absorbed dose and the effective dose due to the injection of the radiopharmaceutical are estimated by the software, taking into account the presence of other Sc-isotopes, namely 46Sc and 48Sc, co-produced in the natV(p,x) nuclear reaction. The amount of produced activity of each Sc-isotope is calculated from the cross-section data on the EXFOR database [5] and the more recent results of the PASTA project [6] to determine the RadioNuclidic Purity (RNP) and the total effective dose (EDt) in different energy ranges (45–19 MeV, 40–19 MeV, 35–19 MeV, 30–19 MeV) and for different irradiations times (24 h and 80 h). The final results are compared to the outcomes obtained previously with the MIRDCalc software and the published data achieved with the OLINDA software [7].

**Results:** The organs’ absorbed doses and the ED obtained with the various software are generally slightly different (up to 50% of discrepancy only in a few cases). The difference is more marked between OLINDA and the other two software due to the different definitions of the phantom implemented in the software. In fact, OLINDA uses a Non-Uniform Rational B-Spline (NURBS) phantom while MIRDCalc and IDAC-Dose2.1 use a voxel phantom.

A complete comparison between the three software is not possible since the 48Sc contaminant is not implemented in MIRDCalc. However, adopting the general criteria of RNP 99% and contribution of the contaminants to the EDt < 10%, the optimal irradiation conditions for the natV(p,x) nuclear reaction are very similar for the different software.

**Conclusion:** A general accordance between the absorbed doses and EDs due to a 47Sc-radiopharmaceutical has been obtained with three different software. The discrepancies can be traced back to the different volumes and masses describing the organs in the phantom models implemented in the software codes. Some further investigation will be performed to improve the knowledge about the sources of the discrepancies between the software results, in the case of the natV(p,x) nuclear reaction.

**References**

1. Andersson M, et al. EJNMMI Res. 2017;7:88.
2. Kesner A, et al. J Nucl Med. 2018;59(1):473.
3. Stabin MG, et al. J Nucl Med. 2005;46:1023–7.
4. Müller C, et al. J Nucl Med. 2014;55:1658–64.
5. EXFOR Database. 2020. https://www-nds.iaea.org/exfor/exfor.htm.
6. Barbaro F, et al. Rev C. 2021;104:044619.
7. De Nardo L, et al. Phys Med Biol. 2021;66:025003.

## PP17

### [^89^Zr]ZrTrastuzumab preparation based on commercial cassette base synthesis module

#### Malachini Matteo, D'Angelo Armando, Cazzola Emiliano, Gorgoni Giancarlo

##### IRCCS Ospedale Sacro Cuore Don Calabria, Negrar (Vr), Italy

*EJNMMI Radiopharmacy and Chemistry* 2023; 8(Suppl1): PP17

**Aim:** [^89^Zr]Zirconium is one of the emergent isotopes due to its favorable PET imaging characteristics (β+max 0.395 MeV; 22.7%) and half-life (T½78.4 h) ideal for antibodies labeling. Monoclonal antibodies (MAbs) are the most approved biopharmaceutical in the word with multiple and selective targets. The immunoPET can facilitate the approval for new MAbs and can help on patient selection. Due to these needs, a robust production, purification and labelling procedure should be optimized on automatic modules in order to minimize the operator dosimetry and increase the reproducibility.

Aim of this work is based on easy modifications of an automatic, cassette-based, commercial module in order to dissolve the solid target and purify the [^89^Zr]Zirconium in oxalate form (1, 2). After the formulation, in the same cassette, the [^89^Zr]ZrOx will be used to label Trastuzumab and finally purify the [^89^Zr]ZrTrastuzumab. The use of disposable cassettes reduce the possibility to accumulate metal impurities, avoids cleaning step, which are instead mandatory when working with synthesis modules based on fixed tubes technology, and facilitate the transfer of the process on a GMP production environment.

**Materials and methods:** A cassette-based module was used to set up an automatic dissolution, purification and labelling procedure.

The solid [^89^Y]Yttrium target was bombarded on TR-19 cyclotron at 12.5 MeV, without degrader, at different currents (20–60 µA) for a variable time 30–240 min. The coin was transferred to a dedicated coated hotcell and finally inserted on a cassette module in order to dissolve and purify the [^89^Zr]/[^89^Y] material. A 2 N HCl solution was used to dissolve the target material, the solution was transferred to a ZR resin and recovered in the oxalate form. The [^89^Zr]ZrOx solution was used to label DFOTrastuzumab. The final purification based on PD10 was performed and the final solution, was evaluated on a standard quality control profile including immunoreactivity.

**Results:** Different test were done to optimizing the process starting to dissolution and purification of [^89^Zr]ZrOx, average recovery yield of 93% were obtained and after 1 h and 30 min the [^89^Zr]ZrTrastuzumab were collected on final vial with an average labelling yield of 95%. After PD10 purification QC were performed to evaluate the quality control profile of the final solution.

**Conclusion:** What we described on this work is one of the possible way to optimize the automatic [^89^Zr]Zirconium production starting from [^89^Y]Yttrium solid target including: dissolution, purification, MAb labelling, [^89^Zr]ZirconiumMAb purification and formulation. This process is based on cassette module to minimize the impurities, avoid any manual operation on radioactive material management and facilitate the regulatory process for radiopharmaceutical application.

**References**

1. Cisternino S, Cazzola E, Skliarova H, Amico J, Malachini M, Gorgoni G, Anselmi-Tamburini U, Esposito J. Target manufacturing by spark plasma sintering for efficient ^89^Zr production. Nucl Med Biol. 2022;104–105:38–46. https://doi.org/10.1016/j.nucmedbio.2021.11.004.
2. Sciacca G, Martini P, Cisternino S, Mou L, Amico J, Esposito J, Gorgoni G, Cazzola E. A universal cassette-based system for the dissolution of solid targets. Molecules. 2021;26:6255. https://doi.org/10.3390/molecules26206255.

## PP18

### Development of ^89^Zr-radiolabelled obinutuzumab: as a potential immuno-PET agent

#### Elif Tugce Sarcan Bozkir^1^, Stephen Paisey^2^, Martin Ruthdart^3^, A. Yekta Ozer^1^, Christopher Marshall^2^, Neil Hartman^4^

##### ^1^Hacettepe University, Faculty of Pharmacy, Department of Radiopharmacy, Ankara, Turkey. ^2^University Hospital of Wales, Wales Research and Diagnostic PET Imaging Centre, Cardiff, Wales, UK. ^3^University Hospital of Wales, Haematology, Division of Cancer and Genetics, Cardiff, Wales, UK. ^4^Singleton Hospital, Head of Nuclear Medicine, Swansea, Wales, UK

*EJNMMI Radiopharmacy and Chemistry* 2023; 8(Suppl1): PP18

**Aim:** Immuno-PET is currently used as a common non-invasive molecular imaging technique which combines PET radionuclides and antibodies/proteins etc. (1). The combination of PET radionuclides and mAbs provides a better target for diseases, tumour targeting and accumulation in tumours. ^89^Zr is a radiometal and a potential tracer for radiolabelling of mAbs and proteins with a long biological half-life (2, 3). In this study, it was investigated that ^89^Zr radiolabelled Obinutuzumab (Obi) as a potential immuno-PET agent for NHL. ^89^Zr-Obi was prepared in different ratios of components (mAb: DFO-Bz-NCS: ^89^Zr) and evaluated by radio-HPLC and stability test.

**Materials and methods:** Formulation studies pH values of Obi samples were adjusted between 8.9–9.1 with 1 M NaHCO_3_. Afterwards, the conjugation process was realized by adding DFO-Bz-NCS in DMSO at 37 °C at 550 rpm for 1 h. 6 different formulations were prepared with different ratios of (Obi: DFO-Bz-NCS). (mAb:DFO-p-Bz-NCS: ^89^Zr) molar ratios of formulations were given in Table 1.

**Table 1:** Formulation ratios.

Formulation 1 (mAb:DFO-p-Bz-NCS: ^89^Zr) molar ratio: 1:10:10-1
Formulation 2 (mAb:DFO-p-Bz-NCS: ^89^Zr) molar ratio: 1:1:10-1
Formulation 3 (mAb:DFO-p-Bz-NCS: ^89^Zr) molar ratio: 1:15:10-1
Formulation 4 (mAb:DFO-p-Bz-NCS: ^89^Zr) molar ratio: 1:10: 2.10-1
Formulation 5 (mAb:DFO-p-Bz-NCS: ^89^Zr) molar ratio: 1:10: 1.4.10-1
Formulation 6 (mAb:DFO-p-Bz-NCS: ^89^Zr) molar ratio: 1:10: 0.6.10-1

After conjugation occurred, the Sephadex G50 column was used for the recovery (mAb: DFO-Bz-NCS) from the reaction mixture with PBS. pH of ^89^Zr in 1 M oxalic acid solution was adjusted to pH 7 with 0.5 M Na_2_CO_3_ and was added to (mAb: DFO-Bz-NCS) conjugation solution in different amounts and incubated for 1 h at 550 rpm at room temperature (4, 5).

Quality control tests

Radiochemical impurities of formulations were analyzed by radio-HPLC. Stability tests were carried out with 9 MBq of ^89^Zr-Obi in 5 mL saline and samples were taken at certain time intervals at 4, 24, 48, 60, 72 and 90 h and analyzed by radio-HPLC (6, 7).

**Results:** The radio-HPLC chromatograms showed a free ^89^Zr peak observed around 40th min whereas ^89^Zr labelled antibodies, proteins etc., peaks were generally obtained between 20th and 30th min. Radio-HPLC results showed that ^89^Zr -Obi was radiolabelled successfully. Also, labelling yields (ROI%) were found within the limits in formulations 1; 3 and 6 for ^89^Zr-Obi although free ^89^Zr was found in formulations 2; 4; 5. Stability results showed that minimal degradation started at 24th h. Stability studies were carried out on the optimum Formulation 2 obtained after the formulation studies. While there was no free ^89^Zr at the 0th and 4th h, ROI (%) of ^89^Zr-Obi was observed as 96.24% and 96.32%, respectively. The degradation was found at 94.13% at 24th h which meant a minimal decomposition of ^89^Zr-Obi started around 24th h. The ratio decreased each time point after 24th h and 84.21% was found at the 48th h. A significant difference was found among 69.95 %, 55.19 % and 48.4% at the time points of 60th, 72th and 90th h, respectively (*P* < 0.05).

**Conclusion:** ^89^Zr-Obi has been labelled by using the DFO-p-Bz-NCS chelating agent with high radiochemical purity. Radio-HPLC results of ^89^Zr-Obi showed high radiochemical purity. Stability studies of ^89^Zr-Obi showed that the formulation was stable for 24 h and (^89^Zr /total) activity was determined as 94.13% at the end of 24th h. Degradation began to be observed after 24 h.

**References**

1. Zettlitz KA, Tavaré R, Knowles SM, Steward KK, Timmerman JM, Wu AM. ImmunoPET of malignant and normal B Cells with (^89^)Zr- and (124)I-labeled obinutuzumab antibody fragments reveals differential CD20 internalization in vivo. Clin Cancer Res. 2017;23(23):7242–52.
2. Dilworth JR, Pascu SI. The chemistry of PET imaging with zirconium-89. Chem Soc Rev. 2018;47(8):2554–71.
3. Sarcan ET, Silindir-Gunay M, Ozer AY, Hartman N. ^89^Zr as a promising radionuclide and it’s applications for effective cancer imaging. J Radioanal Nucl Chem. 2021;330(1):15–28.
4. Vosjan MJ, Perk LR, Visser GW, Budde M, Jurek P, Kiefer GE, et al. Conjugation and radiolabeling of monoclonal antibodies with zirconium-89 for PET imaging using the bifunctional chelate p-isothiocyanatobenzyl-desferrioxamine. Nat Protoc. 2010;5(4):739–43.
5. Knight JC, Paisey SJ, Dabkowski AM, Marculescu C, Williams AS, Marshall C, et al. Scaling-down antibody radiolabeling reactions with zirconium-89. Dalton Trans. 2016;45(15):6343–7.
6. Natarajan A, Habte F, Gambhir SS. Development of a novel long-lived immunoPET tracer for monitoring lymphoma therapy in a humanized transgenic mouse model. Bioconjug Chem. 2012;23(6):1221–9.
7. White JM, Kuda-Wedagedara AN, Wicker MN, Spratt DE, Schopperle WM, Heath E, et al. Detecting TRA-1-60 in cancer via a Novel Zr-89 labeled ImmunoPET imaging agent. Mol Pharm. 2020;17(4):1139–47.

## PP19

### First patient-reported experience using ^99m^Tc-Tektrotyd at the nuclear medicine service in Kosovo

#### Armend Jashari^1,2,4^, Fakir Spahiu^3^, Ismet Bajrami^3^, Emilija Janevik-Ivanovska^1^

##### ^1^Faculty of Medical Sciences, Goce Delcev University Stip, Republic of North Macedonia. ^2^Hospital and University Clinical Service of Kosovo-Shskuk. ^3^Nuclear Medicine Department in Hospital and University Service of Kosovo. ^4^Alma Mater Europaea Campus College "REZONANCA", Republic of Kosovo

*EJNMMI Radiopharmacy and Chemistry* 2023; 8(Suppl1): PP19

**Aim:** This presentation is focused in the introduction of ^99m^Tc-Tectrotyd radiopharmaceutical for visualization of neuroendocrine tumors and the first patient—reported experience in the department of Nuclear Medicine in Pristina after standardization and validation of labelling and quality control procedures.

**Materials and methods:** The realization of this study is based on the idea of how to contribute to having a good safe new radiopharmaceutical product, using a radiopharmaceutical kit, of HYNIC-D-Phe1, Tyr3-Octreotide giving it as ^99m^Tc-HYNIC-TOC.

Tektrotyd or HYNIC—(D-Phe1, Thyr3-Octreotide) trifluoroacetate (Polatom) radiopharmaceutical labelled with ^99m^ Technetium was administered intravenously in a single dose of 370–740 MBq. The examinations were performed using the whole-body technique and SPECT of the selected body parts.

**Results:** Determination of radiochemical purity was performed using validated chromatographic procedures in the hospital radiopharmacy

The RCP of the ^99m^Tc-Tektrotyd samples administered to patients and after labelling was 93 ± 0.43%. Free pertechnetate and colloidal species were absent/were within the permissible limits.

Whole-body scintigraphy and SPECT were performed 2 h and 4 h after the application of ^99m^Tc-Tektrotyd. The obtained results were interpreted taking into account the obtained chromogranin levels and histopathology after taking samples from the suspicious mass.

**Conclusion:** From a practical point of view, we hope that our first experience and obtained results will indicate the importance of using new radiopharmaceuticals labeled with technetium-99m, as in this case ^99m^Tc-Tectrotyd, in the diagnosis and follow-up of patients with neuroendocrine tumors, especially for the diagnosis of which somatostatin receptors are overexpressed (especially subtype 2 and, to a lesser extent, subtypes 3 and 5).The benefit-risk ratio is clearly positive from a clinical point of view.

**References**

1. Sergieva S, Robev B, Dimcheva M, Fakirova A, Hristoskova R. Clinical application of SPECT-CT with ^99m^Tc-Tektrotyd in bronchial and thymic neuroendocrine tumors (NETs). Nucl Med Rev Cent East Eur. 2016;19(2):81–7.
2. Jashari A, Grozdanovska J, Kaçiu Y, Makraduli LJ, Adzi-Andov LJ, Janevik-Ivanovska E. Cost-effective quality control method for radiochemical purity of ^99m^Tc-Tectrotyd used in a Hospital Radiopharmacy unit. Knowl Int J Knowl Pract. 2021;49(4):731–37.

## PP20

### COST action 19114: a European network dedicated to targeted alpha therapy (TAT) with astatine-211

#### Jean-Francois Gestin^1^, Sture Lindegren^2^, Joelle Gaschet^3^, Stig Palm^2^ Jose Reinaldo Chicaro de Freitas^4,^ Dana Niculae^5^, Marek Pruzynski^6^, Petra Kolenc^7^, Andreas I. Jensen^8^, Penelope Bouziotis^9^, Emilija Janevik Ivanovska^10^, Laurent Navarro^11^, Antero Abrunhosa^12^, Holger Jensen^13^, Renata Mikolajczak^14,^ Matthias M. Herth^15^, Mark Konijnenberg^16^, Per Albertsson^17^, François Guerard^18^, Emma Aneheim^19^

##### ^1^Université de Nantes, Inserm, CNRS, Centre de Recherche en Cancérologie et Immunologie Nantes—Angers (CRCINA)—UMR 1232, ERL 6001, F-44000 Nantes, France. ^2^Department of Medical Radiation Sciences, Institute of Clinical Sciences, the Sahlgrenska Academy, University of Gothenburg; 413 45 Gothenburg, Sweden. ^3^Université de Nantes, CNRS, Inserm, CRCINA, 44000, Nantes, France. ^4^FGUMA Marqués de Beccaria, 29010 Málaga, Spain. ^5^Radiopharmaceutical Research Centre, Horia Hulubei National Institute for Physics and Nuclear Engineering, 30 Reactorului Street, Măgurele, 077125 Ilfov, Romania. ^6^Institute of Nuclear Chemistry and Technology, Dorodna 16, 03-195 Warsaw, Poland. ^7^Department of Nuclear Medicine, University Medical Centre Ljubljana, 1000 Ljubljana, Slovenia. ^8^Center for Nanomedicine and Theranostics (The Hevesy Laboratory), DTU Health Technology, Technical University of Denmark (DTU), Ørsteds Plads 345C, 2800 Lyngby, Denmark. ^9^Radiochemical Studies Laboratory, INRASTES, National Center for Scientific Research Demokritos, Aghia Paraskevi 15341 Athens, Greece. ^10^University "Goce Delčev", Faculty of Medical Sciences, Štip, RNMacedonia. ^11^Precirix, Brussels, Belgium. ^12^ICNAS/CIBIT, Institute for Nuclear Sciences Applied to Health, University of Coimbra, Coimbra, Portugal. ^13^Cyclotron and PET unit KF-3982, Copenhagen University Hospital, Copenhagen, Denmark. ^14^National Centre for Nuclear Research, Radioisotope Centre POLATOM, 05-400 Otwock-Swierk, Poland. ^15^Department of Drug Design and Pharmacology, Faculty of Health and Medical Sciences, University of Copenhagen, Universitetsparken 2, 2100 Copenhagen, Denmark. ^16^Department of Radiology and Nuclear Medicine, Erasmus MC, 3000 CA Rotterdam, The Netherlands. ^17^Department of Oncology, Region Vastra Gotaland, Sahlgrenska University Hospital, Gothenburg, Sweden. ^18^Université de Nantes, Inserm, CNRS, Centre de Recherche en Cancérologie et Immunologie Nantes—Angers (CRCINA)—UMR 1232, ERL 6001, F-44000 Nantes, France. ^19^Department of Medical Radiation Sciences, Institute of Clinical Sciences, the Sahlgrenska Academy, University of Gothenburg; 413 45 Gothenburg, Sweden

*EJNMMI Radiopharmacy and Chemistry* 2023; 8(Suppl1): PP20

**Aim:** The main research aim of the Network for Optimized Astatine-Labeled Radiopharmaceuticals (NOAR) COST Action is to successfully demonstrate that TAT with Astatine-211 can become the European standard for treatment of certain cancerous pathologies, such as glioma, multiple melanoma, breast, ovarian, prostate cancer, neuroendocrine tumours, Non Hodgkins Lymphoma, and others.

**Materials and methods:** In order to achieve this main aim, the following points must be addressed: Astatine-211 targetry, production and logistics; correct speciation of At-211 for pharmaceutical production; optimization of vectors for At-211 labeling and development of validated radiolabeling protocols; automation in production of At-211 and At-211 radiopharmaceuticals; development of dosimetry techniques for At-211 TAT; identification of the regulatory environment for TAT using At-211 radiolabeled vectors; investigation of pharmacoeconomics of TAT with At-211; education and communication aspects.

**Results:** Among the expected outputs of the NOAR COST Action CA19114 are the following: a strong capacity to produce At-211 radiopharmaceuticals, by gathering European skills in radiochemistry, chemistry, biology, dosimetry and preclinical as well as clinical research, to conduct clinical trials and facilitate development at a European level; establishment of robust partnerships among participants, thus raising the capacity for further collaboration; training of Early Career Investigators (ECIs) and PhD students through Short Term Scientific Missions (STSMs); establishment of a network which will permit innovative technology on At-211 TAT to be patented and exploited in Europe, particularly by involving ECIs.

**Conclusion:** Efficient exchange of knowledge and efficient networking will be provided within the COST NOAR Action, to bring together all European stakeholders interested in the promotion of At-211 for medical applications and to allow association with partners outside Europe (USA, South Africa, Japan). Joining European and international research efforts will significantly increase the fundamental and applied knowledge on At-211 and will allow Europe to take a technology lead in the field of At-211 TAT.

**References**

1. Lindegren L, Albertsson P, Back T, Jensen H, Palm S, Aneheim E. Realizing clinical trials with astatine-211: the chemistry infrastructure. Cancer Biother Radiopharm. 2020;35(6):425–36. This work was funded by the horizon 2020 Framework Programme of the European Union under the COST Action NOAR “Network for Optimized Astatine labeled Radio-pharmaceuticals” (CA19114).
2. Maingueneau C, Berdal M, Eychenne R, Gaschet J, Chérel M, Gestin J-F, Guérard F. 211At and 125I-labeling of (hetero)aryliodonium ylides: astatine wins again. Chem Eur J. 2022;28(11):e202104169.

## PP21

### Development of a kit preparation for radiolabelling of the SST2 receptor antagonist TECANT1 with technetium-99m

#### Agnieszka Sawicka^1^, Doroteja Novak^2^, Barbara Janota^1^, Anton Hörmann^3^, Marko Kroselj^4^, Alicja Hubalewska^5^, Renata Mikolajczak^1^, Petra Kolenc^4^, Clemens Decristoforo^3^, Piotr Garnuszek^1^

##### ^1^Radioisotope Centre POLATOM, National Centre for Nuclear Research, Otwock, Poland. ^2^Faculty of Pharmacy, University of Ljubljana, Ljubljana, Slovenia. ^3^Department of Nuclear Medicine, Medical University Innsbruck, Innsbruck, Austria. ^4^Department of Nuclear Medicine, University Medical Centre Ljubljana, Ljubljana, Slovenia. ^5^Department of Endocrinology, Jagiellonian University Medical College, Cracow, Poland

*EJNMMI Radiopharmacy and Chemistry* 2023; 8(Suppl1): PP21

**Aim:** Somatostatin receptor imaging is well established but in recent years, development has focused on SST2 receptor (SSTR) antagonists due to their imaging superiority over agonists. The development of ^99m^Tc-labelled SSTR antagonists could provide broad availability and cost effectiveness by using ^99^Mo/^99m^Tc generators. [^99m^Tc]Tc TECANT1, a receptor antagonist based on LM3 (LM3 (p-Cl-Phe-cyclo(D-Cys-Tyr-DAph(Cbm)-Lys-Thr-Cys)-D-Tyr-NH2) attached to tetraamine chelator, showed promising properties in preclinical studies (1). Our primary goal was therefore to provide a reliable method for facile preparation of a ^99m^Tc-labelled SST2 receptor antagonist, [^99m^Tc]Tc TECANT1, in a hospital radiopharmacy setting, suitable for a first-in-human multi-centre clinical trial, based on a cold kit preparation.

**Materials and methods:** In order to ensure successful on-site preparation of a high-quality radiopharmaceutical immediately before administration, a freeze-dried 3-vial kit was developed. The final composition of the kit was established based on the radiolabelling results obtained in the optimization process in which the appropriate precursor mass, pH and buffer were tested. Analytical methods including HPLC and TLC were validated.

**Results:** The prepared GMP grade batches met all predefined specification parameters together with long-term kit stability and stability of radiolabelled product.

**Conclusion:** Based on this kit preparation [^99m^Tc]Tc-TECANT1 is readily available in a suitable pharmaceutical formulation to be advanced into a first-in-human clinical trial.

**Reference**

1. Fani M, et al. Pharmaceuticals. 2020;14(1):19.

## PP22

### Comparison of incubation times of three commercially available kits for preparation of Tc-99m human serum albumin nanocolloids

#### Boštjan Hudomalj, Anja Mihelčič,

##### Onkološki inštitut Ljubljana, Oddelek za nuklearno medicino, Ljubljana, Slovenija

*EJNMMI Radiopharmacy and Chemistry* 2023; 8(Suppl1): PP22

**Aim:** Nanocoll (GE Healthcare) was our standard kit for preparation of Tc-99m HSA nanocolloids since the beginning of sentinel node detection in our facility about 20 years ago. Due to its shortage in 2019 and later discontinuation of production, we were forced to find alternatives in the market. We tried out NanoHSA, manufactured by Rotop, Germany and NanoScan, manufactured by Medi-Radiopharma, Hungary. All three kits have the same amount and similar size distribution of albumin nanoparticles in vials [1, 2, 3], nevertheless they have different incubation times in their instructions for preparation (10–30 min) [1, 2, 3], so we wanted to compare these incubation times and confirm/discard the differences.

**Materials and methods:** In our department we prepare Tc-99m HSA nanocolloid with slightly modified procedure: to gain higher specific radioactivity, we label only 0.2 mg of albumin nanoparticles, which corresponds to 40% of vial content. We achieve this by dissolving vial content in 2.5 mL of saline and removing 1.5 mL from vial. Immediately after we add 1500 mBq of Tc-99m pertechnetate and dilute with saline to final volume of 5 mL.

For determination of radiochemical purity of all three kits we selected the manufacturer's method for quality control of NanoScan, which is simple and fast: ITLC-SG (Agilent Technologies, 12 cm strips) as stationary phase and saline as mobile phase [3]. This method takes about 10 min to complete.

For NanoHSA, which we now regularly use, we compared this RCP method with manufacturer’s: silica gel impregnated plastic sheet (TLC-SG-60, Merck, 12 cm strips) as stationary phase and acetone as mobile phase [2].

**Results:** We labelled 15 kits, 5 of each product with the same lot. They were all prepared by same person, with eluates from same type of Mo-99/Tc-99m generators (Ultra-TechneKow FM, Curium). All eluates were between 4 and 5 h old, previously eluted between 21 and 24 h.

We took samples at six time points after addition of Tc-99m pertechnetate: 10 min, 20 min, 30 min, 60 min, 120 min and 20 h.

RCP of Nanocoll in increasing time points: 89.3%; 92.0%; 93.9%; 96.6%; 97.7% and 98.1%.

RCP of NanoHSA in increasing time points: 95.8%; 96.1%; 97.0%; 98.0%; 98.6% and 98.4%.

RCP of NanoScan in increasing time points: 94.9%; 96.1%; 96.6%; 97.8%; 98.6% and 99.1%.

For NanoHSA we compared two RCP methods by labelling 5 kits. For RCP method with ITLC-SG/saline average result was 95.5% (ranging between 94.6 and 97.4%) and for manufacturer’s method TLC-SG/acetone average was 97.5% (ranging between 96.7 and 99.2%).

**Conclusion:** The best results showed NanoHSA (Rotop) with average RCP of 95.8% (ranging between 95.3 and 96.7%) already after 10 min, which is also prescribed incubation time of manufacturer [2].

Prescribed incubation time of NanoScan (Medi-Radiopharma) is 20 min [3]. We confirmed this with average RCP of 96.1%. ranging between 95.2% and 96.8%.

The slowest rate of labelling was found for Nanocoll (GE Healthcare), which has prescribed incubation time between 10 and 30 min [1]. Our average result after 10 min was 89.3% and after 30 min 93.9% (ranging between 93.6 and 94.3%), not even sufficient for releasing the product for use. But this is not prescribed RCP method by manufacturer.

For NanoHSA we determined that manufacturer’s RCP method with TLC-SG/acetone gives 2.0% higher average result after 10 min than method with ITLC-SG/saline.

**References**

1. Summary of Product Characteristics: Nanocoll (GE Healthcare), revision 09/08/2017.
2. Summary of Product Characteristics: NanoHSA (Rotop), revision 09/04/2018.
3. Summary of Product Characteristics: NanoScan (Medi-Radiopharma), revision 01/06/2016.

## PP23

### A new labelling method of ^99m^Tc-PSMA-HBED-CC

#### Shuichi Shiratori^1,2,3^, Benchamat Phromphao^1,2^

##### ^1^Division of Nuclear Medicine, Department of Radiology, Faculty of Medicine, Chulalongkorn University, Bangkok, Thailand. ^2^Chulalongkorn University Biomedical Imaging Group, Department of Radiology, Faculty of Medicine, Chulalongkorn University, Bangkok, Thailand. ^3^Division of Nuclear Medicine, Department of Radiology, Faculty of Medicine Siriraj Hospital, Mahidol University, Bangkok, Thailand

*EJNMMI Radiopharmacy and Chemistry* 2023; 8(Suppl1): PP23

**Aim:** ^68^Ga-PSMA-HBED-CC (^68^Ga-PSMA-11) was approved by the US FDA as the first PET imaging radiopharmaceuticals for patients with prostate cancer (1). However, utility of ^68^Ga-PSMA-HBED-CC may be limited due to PET/CT or PET/MR accessible and ^68^Ge/^68^Ga generator available (2). Thus, in-house preparation of ^99m^Tc-PSMA-HBED-CC was developed as an alternative to ^68^Ga-PSMA-HBED-CC to ubiquitous affordable in worldwide population.

**Materials and methods:** ^99m^Tc-pertechnetate 370 MBq was added to solution of PSMA-HBED-CC and 4% SnCl_2_·2H_2_O in 10 mL sterile vial, then heated to 100 °C 15 min and incubated while cool down to room temperature. Labelling parameters were optimized to obtain the maximum radiochemical yield of ^99m^Tc-PSMA-HBED-CC. The completeness of chelation was monitored by instant thin layer chromatography (iTLC). Stability of ^99m^Tc-PSMA-HBED-CC was determined.

**Results:** 99mTc-PSMA-HBED-CC was successfully prepared at 100 °C 15 mins using appropriate amount of 10 µg PSMA-HBED-CC and 3 µg SnCl_2_·2H_2_O to reduce Tc-99m 370 MBq in high radiochemical yield (71.49 ± 2.42%), radiochemical purity (98.29 ± 2.65%) and specific activity of 37.84 ± 1.47 GBq/µmol. ^99m^Tc-PSMA-HBED-CC is stable with radiochemical purity more than 95% within 4 h at room temperature.

**Conclusion:** A new labelling method of ^99m^Tc-PSMA-HBED-CC was developed. ^99m^Tc-PSMA-HBED-CC can be administered to patient for SPECT imaging of PCa diagnosis according to IAEA recommendation (3).

**References**

1. FDA news release [Internet]. Silver Spring (MD): U.S. Food & Drug Administration; 2020. FDA Approves First PSMA-Targeted PET Imaging Drug for Men with Prostate Cancer; 2020 December 1 [cited 2022 August 17]; [about 2 screens]. Available from: https://www.fda.gov/news-events/press-announcements/fda-approves-first-psma-targeted-pet-imaging-drug-men-prostate-cancer.
2. Hillier SM, Maresca KP, Lu G, Merkin RD, Marquis JC, Zimmerman CN, et al. ^99m^Tc-labeled small molecule inhibitors of Prostate Specific Membrane Antigen for molecular imaging of prostate cancer. J Nucl Med. 2013;54:1369–76.
3. International Atomic Energy Agency. Quality control in the production of radiopharmaceuticals, TECDOC-1856. IAEA library cataloguing in publication data document 2018.

## PP24

### Influence of the radiolabeling method on in vitro and in vivo stability of a new radiotracer developed to target pancreatic beta cells

#### Ahmadi M, Bacot S, André C, Debiossat M, Ghezzi C and Perret P

##### Université Grenoble Alpes, Laboratoire Radiopharmaceutiques Biocliniques (LRB), Inserm, CHU Grenoble Alpes, 38000 Grenoble, France

*EJNMMI Radiopharmacy and Chemistry* 2023; 8(Suppl1): PP24

**Aim:** M70 is an organic molecule designed to evaluate pancreatic beta cells mass (BCM) using nuclear imaging1. Fragments of antibodies and nanobodies with high specificity and affinity for the chosen target on beta cells were previously evaluated in the laboratory as potential radiotracers of this BCM. However, they presented either a too slow elimination of blood circulation or too high accumulation in the liver. This study aimed to find the best conditions for radiolabeling M70 and its derivative iodo-M70 (IM70) with iodine-125 using different procedures in order to obtain a stable radiotracer ready for use2.

**Materials and methods:** Two known radioiodination procedures were evaluated: (1) oxidation using Iodogen® for labeling of M70 and (2) isotope exchange for labeling of IM70. IM70 is a high lipophilic organic molecule that was only soluble in acetone or corn oil. Due to the solubility problems in aqueous phase, we first studied different concentrations of several excipients to improve the solubility. All radiolabeling parameters were then adjusted. Finally, to achieve a good radiochemical purity, several purification methods had to be evaluated.

**Results:** M70 and IM70 were radioiodinated with a good radiochemical purity. Adding and incubating of a 0.1% w/v of tween to 2 mg/mL of IM70 in acetone allowed holding iodinated IM70 in solution after radiolabeling. However, purification of IM70 was achieved only after the evaluation of several solid phase extraction supports. Both radiotracers were stable in radiolabeling medium for at least 48 h.

In vitro evaluation in mouse blood and in vivo evaluation in mice of M70, radiolabeled using the oxidation method, have shown that there was an immediate deiodination after contact to biological fluid or in vivo injection. On the other hand, IM70 radioiodinated thanks to isotope exchange was stable both in vitro and after in vivo injection in mouse.

**Conclusion:** Despite the availability of many radioisotopes, iodine presents advantages and remains attractive for the development of radiopharmaceuticals, in particular for research investigations of small molecules. Its different radioisotopes can be used for PET/SPECT imaging or radiotherapy (i.e., ^124^I, ^123^I, and ^131^I), and above all, the nature of halogenated atom allows its introduction into a molecule without important changes in its bioavailability. In this study, we have showed that the radiolabeling method has influence on the radiotracer stability and we managed to adjust procedures in order to obtain an injectable radiotracer despite its insolubility.

**References**

1. Eriksson O, et al. In vivo imaging of beta cells with radiotracers : state of the art, prospects and recommendations for development and use. Diabetologia. 2016;59(7):1340–9.
2. Design of radioiodinated pharmaceuticals: structural features affecting metabolic stability towards in vivo deiodination. Eur J Org Chem. 2017;2017(24):3387–414.

## PP25

### Quality of radiopharmaceutical formulations at the Nuclear Medicine Unit, Korle-Bu Teaching Hospital, Ghana

#### Asimen Adu Sarkodie

##### Nuclear Medicine Unit, Korle-Bu Teaching Hospital, Ghana

*EJNMMI Radiopharmacy and Chemistry* 2023; 8(Suppl1): PP25

**Aim:** The quality of radiopharmaceutical formulations for diagnostic imaging at Ghana’s Korle Bu Teaching Hospital (KBTH) Nuclear Medicine Unit has been evaluated.

**Materials and methods:** The radionuclide purity (MoBT), chemical purity, the radiochemical purity of formulated radiopharmaceuticals (^99m^Tc-DTPA, ^99m^Tc-MDP, ^99m^Tc-MIBI) of four (4) ^99^Mo/^99m^Tc generators were assessed using attenuation method, colorimetry, and Instant thin-layer Chromatography-Silica Gel (ITLC-SG) coupled with Gamma Scintillation Spectrometry, respectively. In addition, the accuracies of the well-type γ-counter and the Capintec dose calibrator were examined; and the geometry dependence of the dose calibrator appraised.

**Results:** The respective radionuclide purity of the Na^99m^TcO4 eluates (0.0001- 0.039 µCi ^99^Mo/mCi ^99m^Tc) were lower than the IAEA and US Pharmacopeia recommended MoBT of ≤ 0.15 µCi ^99^Mo/mCi ^99m^Tc. Chemical purity (Al^3+^ breakthrough) of ˂ 10 ppm for all elutions from the four generators satisfies the ≤ 10 ppm recommended Al^3+^ leakage from the generator. RCP for ^99m^Tc-MDP and ^99m^Tc-DTPA ranged 94.2–99.8% and 96.8–99.8% respectively. The estimated RCP for ^99m^Tc-MIBI ranged from 92.6 to 96.8%. The RCPs for ^99m^Tc-MIBI and, ^99m^Tc-MDP in addition to ^99m^Tc-DTPA were comparable to respective recommended RCP ≥ 94% (^99m^Tc-MIBI) plus ≥ 95% (^99m^ Tc-MDP and ^99m^ Tc-DTPA) set by the IAEA, and the United States and European Pharmacopeia. Quality evaluation of the Capintec dose calibrator and well-type γ-counter gave reliable results. The accuracy test for both instruments revealed that calculated percentage (%) errors were within the ± 5% error margin recommended by IAEA. The Capintec dose calibrator response of the ^99m^Tc radioactive source established that 2, 5, and 10 mL syringe volumes had Volume Correction Factors (VCF’s) within the IAEA recommended range of 0.95 ˂ VCF ˂ 1.05. Also, the plot of VCF for 15 consecutive days gave good regression coefficients 10 mL [ R2 = 0.8492]; 5 mL [R2 = 0.962] and 2 mL [R2 = 1].

**Conclusion:** The quality of formulated ^99m^Tc-radiopharmaceuticals (^99m^Tc-DTPA, ^99m^Tc-MDP, ^99m^Tc-MIBI) conformed to internationally recommended criteria.

**References**

1. Boschi A, Uccelli L, Martini P. A picture of modern Tc-99m radiopharmaceuticals: production, chemistry, and applications in molecular imaging. Appl Sci. 2019;9:2526.
2. Shah AS, Hameedullah KA, Khan SU, et al. Comparisons of 99 Mo breakthrough levels in 99 Mo/^99m^Tc generator eluates from two manufacturers and its purification for nuclear medicine imaging. PJR. 2009;19(2):46–9.
3. IAEA. Internationl Atomic Energy Agency. Technetium-99m Radiopharmaceuticals: Manufacture of Kits. IAEA Technical Reports Series. 2008; No. 466. IAEA, Vienna-Austria. STI/DOC/010/466.
4. Ponto JA. Preparation and dispensing problems associated with technetium Tc-99m radiopharmaceuticals. correspondence continuing education courses for nuclear pharmacists and nuclear medicine professionals 2004; Vol. 11, Lesson 1. The University of New Mexico Health Sciences Center College of Pharmacy Albuquerque, New Mexico.
5. Andrade WG, Lima FF. Evaluation of the Eluate Quality of 99 Mo–99m Tc Generators in Recife, Brazil. 2009. In: International nuclear atlantic conference—INAC 2009, Rio de Janeiro, Brazil; September 27-October 2, 2009.
6. IAEA. Internationl Atomic Energy Agency, Quality Assurance for Radioactivity Measurement in Nuclear Medicine. IAEA Technical Report Series. 2006; No. 454, IAEA, Vienna.
7. WHO. Radiopharmaceuticals: General monograh. WHO Drug Information. 2014; Vol. 28, No 2. 162–164.
8. EANM. Radiopharmacy quality control. european association of nuclear medicine. Available at http://www.yumpu.com. Accessed November 18, 2020.
9. Neacsu B, Cimpeanu C, Barna C. Radionuclidic purity—an essential parameter in quality control of radiopharmaceuticals. Roman Rep Phys. 2013;65(1):155–67.
10. UTHealth. Radiation safety program. environmental health and safety 713-500–5840. The University of Texas. Available at https://www.uth.edu. Accessed July 16, 2021.
11. EANM. The radiopharmacy a technologist’s guide. 22–23. Available at http://www.eanm.org. Accessed November 18, 2020.
12. Vincenti LP. Establishing radiopharmaceutical standards at a nuclear medicine unit in Malta. Int J Radiol Imaging Technol. 2016;2(1):2–5.
13. Molavipordanjani S, Hosseinimehr SJ. Fundamental concepts of radiopharmaceuticals quality controls. Pharm Biomed Res. 2018;4(3):1–8.
14. Demeter S, Szweda R, Patterson J, Grigoryan M. Altered [^99m^Tc] Tc-MDP biodistribution from neutron activation sourced 99Mo. J Radioanal Nucl Chem. 2018;316:619–27.
15. Memon SA, Laghari NA, Qureshi ST, Mehdi F. Assessment of molybdenum breakthrough levels in molybdenum-99 / technetium-99m generators: One year experience at NIMRA Jamshoro Pakistan. Int J Radiat Res. 2014;1(4):6–9.
16. Khan KA, Khan G, Zaman UM, et al. Assay for molybdenum-99 breakthrough in pakgen, pinstech generators: a clinical audit at Akuh, Karachi, Pakistan. Pak J Radiol PJR. 2012;22(2):44–5.
17. Momennezhad M, Zakavi SR, Sadeghi R. Determination of molybdenum-99 contamination in Technetium-99m elute obtained from 99Mo/99mTc generator. Iran J Radiat Res. 2010;8(1):31–5.
18. Ibrahim I, Zulkifli MH, Bohari Y, Zakaria I, Wan Hamirul BWK. Minimising molybdenum-99 contamination in technetium-99m pertechnetate from the elution of 99Mo/ 99mTc generator. Jurnal Sains Nuklear Malaysia. 2010;22(1):9–16.
19. Uccelli L, Boschi A, Pasquali M, et al. Influence of the generator in-growth time on the final radiochemical purity and stability of 99mTc radiopharmaceuticals. Sci Technol Nucl Install. 2013.
20. Schwalfenberg GK. The alkaline diet: Is there evidence that an alkaline pH diet benefits health? J Environ Public Health. 2012;727630. 10.1155/2012/727630.
21. Hammermaier A, Reich E, Bögl W. Chemical, radiochemical, and radionuclide purity of eluates from different commercial fission99Mo/99mTc generators. Eur J Nucl Med. 1986;12:41–46. 10.1007/BF00638794.
22. Taşdelen B. ITLC-SG (Instant Thin- Layer Chromatograpy) St. Ph (Stational phase) Mob. Ph (Mobile phase) Loc. of Bou. Ag (Location of bound agent). J Radioanal Nucl Chem. 2011;290(2):283–287.
23. Sadeghpour H, Alavi M, Shahedi M, Entezarmahdi SM, Sakhteman A. Evaluation of radiochemical purities of some radiopharmaceuticals in Shiraz Namazi Teaching Hospital. Trends Radiopharm Scie. 2015;1(1):15–19.
24. Amin MR, Haque A, Biswas A, Mozumder TH. Preparation and Labeling of Technitium-99m Kit in Pharmaceutical Grade Clean Room. J Chem Eng IEB. 2012;27(2):31–5.
25. Ahmed H, Zimmer M, Cutrera P, et al. Radichemical purity and stability of generic Tc-99m DTPA. J Nucl Med. 2015;56(supplement 3), 2740–2740.
26. Abdallah Y, Gar-elnabi ME, Ahmed S, Abdelmajed N. Evaluation of radiochemical purity of received 99Mo/99mTc generator using paper chromatography test. Asian J Med Radiol Res. 2012;5(1):4–10.
27. Miranda ACC, Durante ACR, Fuscaldi LL, de Barboza MF. Current approach in radiochemical quality control of the 99mTc-radiopharmaceuticals: a mini-review. Braz Arch Biol Technol. 2019;62:1–7.
28. Arias JA, Garcia S, Cuadrado ML, et al. 99mTc-MIBI muscle imaging and approach to assess functional anatomy of lower limb muscles. Available at www.semanticscholar.org . Accessed June, 26 2021.
29. Doroudi A, Saraji F, Erfani M, et al. Etesami, B. The stability of 99mTc-MIBI (Sestamibi) complex samples which prepared under ultrasound irradiation technique versus boiling water bath method. J Appl Pharm Sci. 2016;6(11):126–134.
30. Mwape E. Performance evaluation and cross-calibration of capintec Crc 15r and comecer dose calibrators. Available at http://ugspace.ug.edu.gh/handle/123456789/25818. Accessed June 18, 2021.
31. Biodex Medical Systems. Quality Assurance Testing of AtomLab Dose Calibrators (Manufacturer's Instructions). Available at http://www.biodex.com-quality assurance testing. Accessed March 13, 2021.

## PP26

### Radioisotopic exchange reactions for biomolecule pet radiolabeling

#### Álvaro Erhard^1^, Laura Confalonieri^2^, Daniela Imperio^2^, Marcin Balcerzyk^1^, Luigi Panza^2^

##### ^1^Centro Nacional de Aceleradores, Sevilla, Spain. ^2^Università degli Studi del Piemonte Orientale

*EJNMMI Radiopharmacy and Chemistry* 2023; 8(Suppl1): PP26

**Aim:** Radiolabeling of biomolecules using fluorine-18 through the linking carbon—fluorine is a complicated task because of the hard conditions required. Direct radiolabeling using a heteroatom—fluorine linking could solve these complications. The aim is to optimize the radiolabeling and purification method using a radio-isotopic exchange reaction for biomolecules using fluorine-18.

**Materials and methods:** [^18^F]F- was produced in a cyclotron ^18^O(p,n)^18^F and was used directly for radiolabeling or was retained in a suitable cartridge, eluting with NaCl 0.9% and HCl 0.5M (pH = 2.0) acidic [^18^F]F- solution. For radiolabeling, 50 µL of [^18^F]F- or acidic [^18^F]F- was added to the solid precursor and when applicable, pH was adjusted to 2.0 using HCl 0.5M using pH-strips. Radiolabeling was evaluated at 40 and 85 °C using a water-bath (acidic [^18^F]F- was evaluated only at 85 °C). Radiolabeling stability was analyzed in saline and human plasma (1 h, agitation, 37 °C).

Using a micropipette, spots of 1.5 µL were placed in the origin of a TLC-strip at defined period of times to evaluate the radiolabeling kinetics. The TLC method used SilicaGel 60 F254 as stationary phase (10 × 90 mm) and acetonitrile/water 95:5 as mobile phase. The strips were analyzed using a radioTLC equipment.

For reformulation, 0.5 mL of saline was added to the reaction mixture and passed through an Alumina cartridge for purification. Radiolabeling purity was analyzed using the TLC method described previously and the pH was measured using pH-strips.

**Results:** Radiolabeling yields was 37.75% ± 0.02 and 42.4% ± 0.01 when using a radiolabeling time up to 60 min at 40 °C and 45 min at 85 °C, respectively. Purification of the compound provide a purity of 96.9% ± 0.01 at 40 °C and 96.6% ± 0.01 at 85 °C.

Radiolabeling yield was 49.88% ± 0.09 using a radiolabeling time up to 20 min at 85 °C using the acidic [^18^F]F- solution. Purification gave a purity of 97.2% ± 0.01. A pH of 5.5 was found in all the procedures applied.

Stability studies revealed a purity of 97.18% and 95.05% up to 60 min in saline and human plasma, respectively.

**Conclusion:** Radiolabeling and purification of the biomolecule has been achieved in a simple, efficient and stable manner. The use of acidic [^18^F]F- allows to control the pH of the solution, avoiding greater exposure to radiation and reducing Havar compounds in the final product.

**Reference**

1. Jiyuan Li, Yaxin Shi, Zizhu Zhang, Hui Liu, Lixin Lang, Tong Liu, Xiaoyuan Chen, and Zhibo Liu. A metabolically stable boron-derived tyrosine serves as a theranostic agent for positron emission tomography guided boron neutron capture therapy. Bioconjug Chem. 2019;30(11):2870–8. 10.1021/acs.bioconjchem.9b00578

## PP27

### Development of ready to use kit formulation for trastuzumab radioimmunoconjugates and identification of radiochemical purity as the first step in quality control of the final product

#### Marija Arev^1^, Sanja Vranješ-Đurić^2^, Drina Janković^3^, Marija Mirković^2^, Magdalena Radović^2^, Paulina Apostolova^1^, Emilija Janevik-Ivanovska^1^

##### ^1^University ‘Goce Delčev’, Faculty of Medical Sciences, str. “Krste Misirkov” No. 10-A, 2000 Štip, Republic of North Macedonia. ^2^Vinča Institute of Nuclear Sciences, University of Belgrade, Mike Petrovića Alasa 12-14, 11001 Belgrade, Serbia

*EJNMMI Radiopharmacy and Chemistry* 2023; 8(Suppl1): PP27

**Aim:** The aim of this study is to present the part of our project dedicated to obtaining a stable, ready to use freeze dried kit formulation of antibody radioimmunoconjugates (trastuzumab immunoconjugates labelled with ^90^Y and ^177^Lu). As the first step in on-going in vitro stability of the final product and radiochemical purity determination, we used ITLC-SG method with different mobile phases.

**Materials and methods:** Radioactive labelling of trastuzumab was performed with ^90^Y and ^177^Lu via DOTA, DTPA and 1B4M-DTPA in molar ratio 1:20. The specific activity of 1.425 mCi (^90^Y) and 8.150 mCi (^177^Lu) was achieved, using a solutions of 0.04 M HCl. Radiolabeling is performed by adding 8.5 µL of ^90^Y at pH 4.5–5 and 5 µL ^177^Lu at pH 6. Solutions with Tr-DTPA and Tr-1B4M-DTPA were incubated at room temperature for 30 min, while Tr-DOTA was incubated at 40 °C for 1 h.

Radiochemical purity of radioisotopes was tested with ITLC-SG using three mobile phases: 0.9% NaCl, 0.4 M methanol/sodium-acetate (1:1) and 0.1 M acetic buffer.

The stability of radioimmunoconjugates was tested in 0.9% NaCl (^177^Lu) and 0.4 M methanol/sodium-acetate (1:1) (^90^Y), after incubation at room temperature for 1, 24, 48 and 72 h.

**Results:** After choosing the most suitable mobile phase for determination of radiochemical purity by ITLC-SG of conjugates labeled with ^90^Y (99.87%) we used 0.4 M methanol/sodium acetate (1:1), and those with ^177^Lu (100%) with 0.9%. NaCl. Examination of radiochemical yield of radioimmunoconjugates showed the presence of radioactivity only at the start of the strip, due to the high Mw of Tr. The absence of radiolabeled fragments of the antibody, as well as radiolabeled chelators and free radioisotopes, proved that the stable radioimmunoconjugates were formulated. The highest yield of labeling with ^90^Y (96%) is achieved in 0.4 M methanol/sodium-acetate (1:1), while with ^177^Lu 99%) in 0.9% NaCl.

Test stability after 24 h showed the highest stability of ^90^Y-DOTA-Tr (92.40%) and ^177^Lu-DOTA-Tr (99.14%), with minimum released ^90^Y^3+^ (7.60%) and ^177^Lu^3+^ (84.90%) and ^177^Lu-DOTA-Tr (98.52%), with minimum released ^90^Y^3+^ (15.10%) and ^177^Lu^3+^ (1.48%).

**Conclusion:** After obtaining the final ready to use kit formulation, the results of the determination of radiochemical purity using ITLC-SG show a high radiolabeling efficiency (95%), using both isotopes. However, radioactive yield with ^177^Lu (99%) was higher compared with ^90^Y (96%). This method was used to monitor the stability of radiolabeled conjugates and after 72 h of incubation, a small amount of free radioisotopes was released from radioimmunoconjugates (5% of ^177^Lu and 25% of ^90^Y).

The next planed step includes in vivo examinations in healthy mice and in a mouse model of HER2 positive breast tumor after the i.v. injection of radiolabeled trastuzumab radioimmunoconjugates in order to monitor and determine their pharmacokinetics and biodistribution in the whole body and critical organs/tumor.

**References**

1. Bhusari P, Vatsa R, Singh G, Parmar M, Bal A, Dhawan DK, Mittal BR, Shukla J. Development of Lu-177-trastuzumab for radioimmunotherapy of HER2 expressing breast cancer and its feasibility assessment in breast cancer patients. Int J Cancer. 2017;140(4):938–47.
2. Blend MJ, Stastny JJ, Swanson SM, Breshbiel MW. Labeling anti-HER2/neu monoclonal antibodies with ^111^In and ^90^Y using a bifunctional DTPA chelating agent. Cancer Biother Radiopharm. 2003;18(3):355–63.

## PP28

### Surface and equipment maintenance in an hospital radiopharmacy

#### C. Bouvry, V. Ardisson N. Lepareur, C. Bertrand

##### Centre de Lutte Contre le Cancer Eugène Marquis, 35042 Rennes, France

*EJNMMI Radiopharmacy and Chemistry* 2023; 8(Suppl1): PP28

**Aim:** In radiopharmacy, use of isopropanol is common for disinfection of septa and some surfaces like leaded glass windows (1). Few days after installation of a new high-energy hotcell within our radiopharmacy unit, some cracks appeared on the plexiglas inside shield of the hotcell. First investigations demonstrated use of an inappropriate cleaning product (isopropanol) on that surface could be the cause for that. Our objective is to validate cleaning products and procedures for the safe maintenance of our equipment.

**Materials and methods:** We have collected and analysed the various technical sheets of the referenced maintenance products. We have sent the products to the hotcell manufacturer for them to evaluate their compatibility with surfaces. We have looked for recommendations extended to the materials available within our radiopharmacy unit: syringe shields, activimeter’s sock and ladle, gloves, etc.

**Results:** Cleaning products have varied compositions: detergents-disinfectants with or without quaternary ammonium salts, alcohol-containing disinfectants… Our cleaning procedures have been reevaluated and a compatibility table has been realised, correlating cleaning products, surfaces and materials. Our maintenance procedure for hotcells has been validated by the hotcell manufacturer.

**Conclusion:** This incident enabled us to reevaluate our practice regarding the maintenance of our equipment and hotcells. Our table will be updated each time referenced maintenance products will change.

**Reference**

1. Gillings N, Hjelstuen O, Ballinger J, et al. Guideline on current good radiopharmacy practice (cGRPP) for the small-scale preparation of radiopharmaceuticals. EJNMMI Radiopharm Chem. 2021;6:8.

## PP29

### Improved radio-synthesis of [^68^Ga]Ga-DOTA-TATE using freeze-dried kit

#### Marcin Radzik, Barbara Janota, Wioletta Wojdowska, Piotr Garnuszek

##### National Centre for Nuclear Research Radioisotope Centre POLATOM, Otwock, Poland

*EJNMMI Radiopharmacy and Chemistry* 2023; 8(Suppl1): PP29

**Aim:** Several ^68^Ga-labeled somatostatin analogs, such as [DOTA0, Tyr3]-octreotide (DOTA-TOC), [DOTA0, 1-NaI3]-octreotide (DOTA-NOC) and [DOTA0, Tyr3, Thr8]-octreotide (DOTA-TATE) have been successfully applied for the identification of a variety of tumors, particularly neuroendocrine tumors [1, 2]. Due to the excellent clinical data of ^68^Ga-labeled somatostatin analogs the interest for [^68^Ga]Ga-DOTA-TATE is steadily increasing. However, there are some unresolved issues with impurities affecting product quality.

The aim of this study was to improve a radio-synthesis of [^68^Ga]Ga-DOTA-TATE to obtain a high radiochemical purity of the final product, which meets the requirements of the European Pharmacopoeia monograph [3].

**Materials and methods:** All reagents were Ph.Eur or Pharma grade. DOTA-TATE was obtained from POLATOM (Poland), ^68^GaCl3 was obtained from IRE Elit Galli Eo ^68^Ge/^68^Ga generator (Belgium) using 0.1 M hydrochloric acid (0.1 M HCl) for elution, sodium acetate and acetic acid were obtained from Sigma-Aldrich and AppliChem, ascorbic acid was obtained from AppliChem. Vials, stoppers and caps were obtained from West Pharmaceutical. 0.2 µm sterile filters was obtained from Whatman. For radiolabeling 40 µg of DOTA-TATE and specific amount of excipients was incubated at 95 °C for 15 min. Radiochemical purity of radiolabeled [^68^Ga]Ga-DOTA-TATE was assessed by iTLC and HPLC methods.

**Results:** Kit composition was based on sodium acetate, which is widely used as a buffer for radiolabeling [4]. Influence of different amounts of sodium acetate on radiochemical purity was tested. According to literature [5, 6] addition of ascorbic acid and/or ethanol to reaction mixture improves radiochemical purity of [^68^Ga]Ga-DOTA-TATE. This case also was verified. In preliminary studies, the composition of the kit was optimized. Kit with the optimal composition was then lyophilized in sterile Class-A conditions and tested for compliance with Ph. Eur monograph.

**Conclusion:** In the course of the studies performed, main problem was a high amount of ^68^Ga in colloidal form after radiolabeling of the kit, which was visible in TLC analysis. The high amount of ^68^Ga-colloid was maintained regardless of the composition of the kit. Post radiolabeling filtration through a 0.2 µm sterile filter was introduced to remove the ^68^Ga-colloid. Significant improvement on the radiochemical purity of [^68^Ga]Ga-DOTA-TATE was achieved by the addition of ascorbic acid to the reaction mixture.

**References**

1. Łapińska G, Bryszewska M, Fijołek-Warszewska A, Kozłowicz-Gudzińska I, Ochman P, Sackiewicz-Słaby A. The diagnostic role of ^68^Ga-DOTATATE PET/CT in the detection of neuroendocrine tumours. Nucl Med Rev. 2011;14(1):16–20.
2. Hofman MS, Eddie Lau WF, Hicks RJ. Somatostatin receptor imaging with ^68^Ga DOTATATE PET/CT: clinical utility, normal patterns, pearls, and pitfalls in interpretation. RadioGraphics. 35(2).
3. Gallium (^68^Ga) Edotreotide injection monograph 01/2022:2482.
4. Bauwens M, Chekol R, Vanbilloen H, Verbruggen A, Bormans G. Optimal buffer choice of the radiosynthesis of ^68^Ga-Dotatoc for clinical application. Nucl Med Commun. 31(8):753–8.
5. Sriwiang W, Phumkhem S, Thida S. Formulation of DOTATATE cold kit for preparation of ^68^Ga radiopharmaceutical. In: 2019—The 7th Burapha University international conference on interdisciplinary research.
6. Mu L, Hesselmann R, Oezdemir U, Bertschi L, Blanc A, Dragic M, Loffler D, Smuda C, Johayem A, Schibli R. Identification, characterization and suppression of side-products formed during the synthesis of high dose ^68^Ga-DOTA-TATE. Appl Radiat Isot. 2013;76:63–9.

## PP30

### Status and development of radiopharmacy/nuclear medicine services in Africa and the formation of the African Association of Radiopharmacy (AfrAR)

#### Yohannes Jorge Lagebo^1^ Naoual Bentaleb^2^

##### ^1^School of Medicine-Cholege of Health Sciences,Addis Ababa University and Currently President of the African Association of Radiopharmacy(AfrAR), Addis Ababa, Ethiopia. ^2^National Centre for Nuclear Energy, Sciences and Technologies (CNESTEN) Rabat, Morocco

*EJNMMI Radiopharmacy and Chemistry* 2023; 8(Suppl1): PP30

**Aim:** The objectives of this survey is to identify the existing status and the developmental prospects of Radiopharmacy/Nuclear Medicine Services in Africa and the possible impacting roles of the formation of the African Association of Radiopharmacy (AfrAR) in enhancing these developments.

**Materials and methods:** The survey was conducted through literature review, administering the semi-structured questionnaires, review of the African countries reports on radiopharmacy services/practices via their Consecutive Annual Progress Reports on the IAEA AFRA Regional Radiopharmacy Projetcs "Strengthening and Improving Radiopharmacy Services in Africa" such as RAF2008, RAF6049, and RAF6054 ; one to one interview administered to professionals and via critical personal observations of the experienced radiopharmacy/nuclear medicine professionals as well as using the AfrAR establishment documents.

**Results:** The survey based studies indicated that unlike most countries in the other continental regions of the globe, there are still several African countries where the radiopharmacy/nuclear medicine services have not even been initiated. Among the 54 African countries only 27 countries (50%) have started the radiopharmacy/nuclear medicine services. Even the started services in some of these countries are not satisfactory and not in pace with the accepted scope and standard level of patient services. However, since the starting of the first AFRA Regional Radiopharmacy Project (RAF2008) in 2008 the project participating countries started increasing in the region.The continuation of this IAEA regional radiopharmacy project via their consecutively continuing projects like RAF6049 & RAF6054 and the formation of the AfrAR in March 2015 have stared being positively impacting in enhancing the development of radiopharmacy/nuclear medicine services within the African continent especially since the last 5–7 years.The number of MSc trained radiopharmacists within the continent has also stared increasing significantly since this period due to the two additional initiated MSc programs(1 in Anglophone and 1 in francophone) besides the earlier existed one in English making the total MSc programs three in Radiopharmacy. The PET-Radiopharmacy and molecualr nuclear medicine imaging patient services were limited only to few African countries in the Northern part of the continent and South Africa. But now since the last 3 years several countries in the East, West and the Center have already started working towards materializing these services. The continuation of the IAEA AFRA Radiopharmacy Regional project and the establishment as well as the formal launching of the AfrAR has started being instrumental to the currently observed visible development of radiopharmacy/nuclear medicine services of the continent. The AfrAR plays the role in creating the professional, scientific and ethical awareness 'among the practicing radiopharmacy professionals within the continent in faithfully serving patients with high level dedication.

**Conclusion:** The Radiopharmacy/Nuclear Medicine services being rendered within the African continent is still low compared to countries within the other continents of the our Globe. However, some improvements are being observed since the last 5–7 years and this is also being more pounced since the last 3 years which is mainly attributed to the continuation of the IAEA AFRA Regional Radiopharmacy projects, the formation of the AfrAR and the increased awareness/sensitivities of the the respective African member states towards the decisive usefulness of the radiopharmacy/nuclear medicine services for their respective patients. If these radiopharmacy IAEA-AFRA Regional project are allowed to continue in the more strengthened and organized manner without being disrupted; the AfrAR is strengthened to the level to fully resume its professional and scientific responsibilities as per its stipulated objectives and if the awareness/sensitivities of the African member countries on the vitality of the radiopharmacy/NM services is made enhanced using different approaches, the indicated African services can be improved significantly without taking longer time as used to be.

**References**

1. Lagebo YJ, Marie AA. Survey on arduous challenges and possible tracks of heightening the radiopharmacy and nuclear medicine services in Africa (Poster Presentation). 28 October–1 November 2019. International Symposium on Trends in radiopharmaceuticals (ISTR-2019).Vienna, Austria. Programme & Abstacts, page 228.
2. The Consecutive Annual Progress Reports on the IAEA-AFRA Regional Radiopharmacy Projetcs "Strengthening and Improving Radiopharmacy Services in Africa", RAF2008, RAF6049, and RAF6054.
3. The IAEA Report of the Final Regional Coordination Meeting of RAF6054 ‘Strengthening and Improving Radiopharmacy Services (AFRA)’ 28 February–4 March 2022, Rabat, Morocco.
4. The IAEA Report of the Final Regional Coordination Meeting of “RAF6049-Strengthening and Improving Radiopharmacy Services (AFRA)”, 23–27 April, 2018, Kampala, Uganda.
5. The IAEA Report on the Final Coordination Meeting (18–20 November 2013) and the First Symposium on Radiopharmacy in Africa (21–22 November 2013), Addis Ababa-Ethiopia.
6. The IAEA Report on the First African Combined Coordination Meeting for Project Coordinators of Hospital Radiopharmacists and Regulators of Radiopharmaceuticals under the Regional Radiopharmacy Project(RAF6049) which was organized by the IAEA in Vienna-Austria from 23–27 March 2015.
7. The IAEA Report on the AFRA-Mid-Term Coordination Meeting of RAF6054 conducted in Pretoria, South Africa, from 4–8 November 2019.
8. The AfrAR Establishing documents: the AfrAR Consitution and the AfrAR Bylaws.
9. Marie AA, Lagebo YJ, Apostolova P, Tripunoski T, Ivanovska EJ. Technetium-99m labelled Human immunoglobulin G polyclonal antibody—different approach for better results(Poster Presentation). 28 October–1 November 2019. International Symposium on Trends in radiopharmaceuticals (ISTR-2019), Vienna, Austria. Programme & Abstacts,Page 186.

## PP31

### Quantitative assessment of radioactivity losses in administration of therapeutic doses of radiopharmaceuticals based on the example of [^177^Lu]Lu-DOTA-TATE

#### Izabela Cieszykowska, Paweł Rybak, Małgorzata Żółtowska, Tomasz Janiak, Renata Mikołajczak, Piotr Garnuszek

##### National Centre for Nuclear Research, Radioisotope Centre POLATOM, Andrzej Sołtan 705-400 Otwock, Poland

*EJNMMI Radiopharmacy and Chemistry* 2023; 8(Suppl1): PP31

**Aim:** Within DuoNen, the phase 3, multicenter, non-commercial clinical study (EudraCT No. 2020-006068-99) therapeutic doses of mixed [^177^Lu]Lu-DOTA-TATE/[^90^Y]Y-DOTA-TATE are prepared in our lab. DuoNEN clinical trial protocol is designed with the aim to develop the optimal algorithm of PRRT for patients with disseminated NET based on personalized dosimetry.

The investigational medicinal product (IMP) DuoNEN consists of two components with varying radioactivities: component No. 1—[^177^Lu]Lu-DOTA-TATE with radioactivity of 0.93–7.4 GBq and component No. —[^90^Y]Y-DOTA-TATE with radioactivity of 0–1.85 GBq.

In personalized therapy based on dosimetry, it is extremely important that the dose administered to the patient is prepared according to the calculated radiation dose estimates. While there are procedures in place for the precise radioactivity dose measurements, the loss related to the administration procedure may also influence the radiation dose to the patient. The aim of this work was to evaluate the loss of radioactivity when administered to the patient using two methods: direct intravenous injection and gravity method.

**Materials and methods:** In order to assess the loss of radiopharmaceutical during administration to the patient, the test were performed in conditions simulating the direct intravenous injection and the gravity administration. The solutions of [^177^Lu]Lu-DOTA-TATE in ascorbic acid buffer with radioactivity of 7.4 GBq and radioactive concentration of 1.0 GBq/mL ± 10% were used in each experiment. We used only ^177^Lu-labelled formulation because the accuracy of the measurement of ^177^Lu radioactivity is less geometry-dependent when compared to ^90^Y. The radioactivity of ^177^Lu at the level of 7.4 GBq is the highest radioactivity of [^177^Lu]Lu-DOTA-TATE component in DuoNEN, thus representing the worst case scenario.

In the simulation of radiopharmaceutical administration using the direct method, the solution of [^177^Lu]Lu-DOTA-TATE was withdrawn quantitatively from the vial into a 10 mL syringe, fitted with a tungsten shield, and the solution was then transferred to an empty vial as a single shot. The syringe was rinsed with 5 mL of saline and the rinsing solution was injected into another empty vial. At each step the radioactivity in the vials and syringes was measured.

In the simulation of radiopharmaceutical administration by gravity method, two needles with tubing were inserted through the rubber septum into the vial containing [^177^Lu]Lu-DOTA-TATE, one connected to 100 mL saline bag to flush out radiopharmaceutical and the other to collect the radiopharmaceutical solution after flushing the vial with saline. The saline bag was hung above the level of the vial, which caused the gravity flow of saline and washing out of radiopharmaceutical solution from the vial. To collect the solution in the vials there were 11 vials needed in total. At each step the radioactivity in the vials with collected solution as well as the residual radioactivity in the product vial was measured in a dose calibrator. Experiments for each method were repeated in 7 independent experiments for statistical evaluation.

All measurements were performed in CRC®-55tR Dose Calibrator (Capintec) with 137Cs as a control source. The extended uncertainty of activity measurement was 2.5% and precision of activity measurement was 0.1%.

**Results:** About 0.71 ± 0.18% (n = 7) of radioactivity of [^177^Lu]Lu-DOTA-TATE remained in the vial after solution withdrawal with the syringe and another 1.51 ± 0.35% (n = 7) remained in the syringe (non-rinsed). Rinsing the vial and the syringe with 5 mL of saline resulted in the reduction of ^177^Lu residues 0.12 ± 0.08% (n = 7) in the vial and 0.10 ± 0.08% (n = 7) in the syringe. In gravity method the total loss of ^177^Lu when [^177^Lu]Lu-DOTA-TATE was washed out from the vial with 100 mL of saline was 0.50 ± 0.24% (n = 7). About 80% of [^177^Lu]Lu-DOTA-TATE was collected in the first 10 mL of saline and the next 15% with another 10 mL. It took 15 min to empty the saline bag; thus, the gravity flow rate of the solution was 400 mL/h.

**Conclusion:** In the conditions simulating the procedure of radiopharmaceutical administration to the patient we observed that loss of ^177^Lu arising from the direct administration of [^177^Lu]Lu-DOTA-TATE to the patient was at the level of 0.2%, while for the gravity method it was at the level of 0.5%. For the radiopharmaceutical dose of 7.40 GBq, it would mean administration with 7.38 GBq and 7.36 GBq, respectively. Bearing in mind the error of measurement, these losses can be considered negligible. One may expect, that the losses of ^90^Y during administration of mixed doses of [^177^Lu]/[^90^Y]Y-DOTA-TATE are at the similar level, since the chemical composition of both is essentially the same.

**Acknowledgments**

The study was partly financed by the Medical Research Agency (Project number 2019/ABM/01/00077-00).

## PP32

### Establishing a basis for therapeutic efficacy studies for targeted Auger electron therapy by standardized comparison of radiation response in 2D and 3D cell culture models of different human cancer cell lines

#### Julia Raitanen^1,2,3,4^, Bernadette Barta^1^, Marcus Hacker^2^, Dietmar Georg^5^, Theresa Balber^1,2^, Markus Mitterhauser^1,2,4^

##### ^1^Ludwig Boltzmann Institute Applied Diagnostics, Vienna, Austria. ^2^Department of Biomedical Imaging and Image-guided Therapy, Division of Nuclear Medicine, Medical University of Vienna, Vienna, Austria. ^3^University of Vienna, Vienna Doctoral School of Chemistry (DoSChem), Vienna, Austria. ^4^University of Vienna, Faculty of Chemistry, Institute of Inorganic Chemistry, Vienna, Austria. ^5^Department of Radiation Oncology, Division of Medical Radiation Physics, Medical University of Vienna, Vienna, Austria

*EJNMMI Radiopharmacy and Chemistry* 2023; 8(Suppl1): PP32

**Aim:** Radiation therapy is one of the most effective tools in cancer therapy, contributing towards 40% of cancer cure [1]. However, success varies individually, which necessitates improved understanding of radiobiology. Three-dimensional (3D) tumor spheroids are recently gaining attention being a superior in vitro cancer model compared to 2D cell culture [2]. In contrast to monolayer culture, multicellular tumor spheroids mimic in vivo tumors more accurately, due to similar growth behavior as well as cellular heterogeneity within the spheroid and formation of molecular gradients, among others [3].

**Materials and methods:** This in vitro study aimed at establishing a basis for therapeutic efficacy studies for targeted Auger Electron therapy by standardized comparison of radiation response in 2D and 3D cell culture models of different human cancer cell lines (PC-3, LNCaP and T-47D), irradiated with varying doses (0, 2, 4, 8 and 20 Gy) of external beam using an YXLON Maxishot X-ray unit. Radiation response was analyzed by growth observation, various cell viability assays (e.g., clonogenic assay, Alamar Blue assay) and amount of DNA damage (γH2AX Western Blot).

**Results:** Results showed decreasing cell proliferation with increasing radiation dose for all cell lines in monolayers and spheroids of LNCaP and T-47D. However, significantly lower radiosensitivity was shown in spheroids, most pronounced in PC-3, suggesting radioresistance of PC-3 spheroids up to 8 Gy and significant growth inhibition only by dose escalation to 20 Gy. Comparison of cell lines showed highest radiosensitivity in LNCaP, followed by T-47D and PC-3.

**Conclusion:** In conclusion, the results indicate that the use of higher dimensionality cell culture models is indispensable to more accurately predict treatment outcome in vivo. Furthermore, this study created the basis for future therapeutic efficacy studies for targeted Auger electron therapy.

**References**

1. Baskar R, Lee KA, Yeo R, Yeoh KW. Cancer and radiation therapy: current advances and future directions. Int J Med Sci. 2012;9(3):193–9.
2. Brüningk SC, Rivens I, Box C, Oelfke U, ter Haar G. 3D tumour spheroids for the prediction of the effects of radiation and hyperthermia treatments. Sci Rep. 2020;10(1):1–13.
3. Jensen C, Teng Y. Is it time to start transitioning from 2D to 3D cell culture?. Front Mol Biosci. 20207:1–15.

